# Supplementary figures and images for: Quantifying impacts of stony coral tissue loss disease on corals in Southeast Florida through surveys and 3D photogrammetry
Source: PLoS One. 2021 Jun 25;16(6):e0252593. doi: 10.1371/journal.pone.0252593 (PMC8232449; doi:10.1371/journal.pone.0252593)

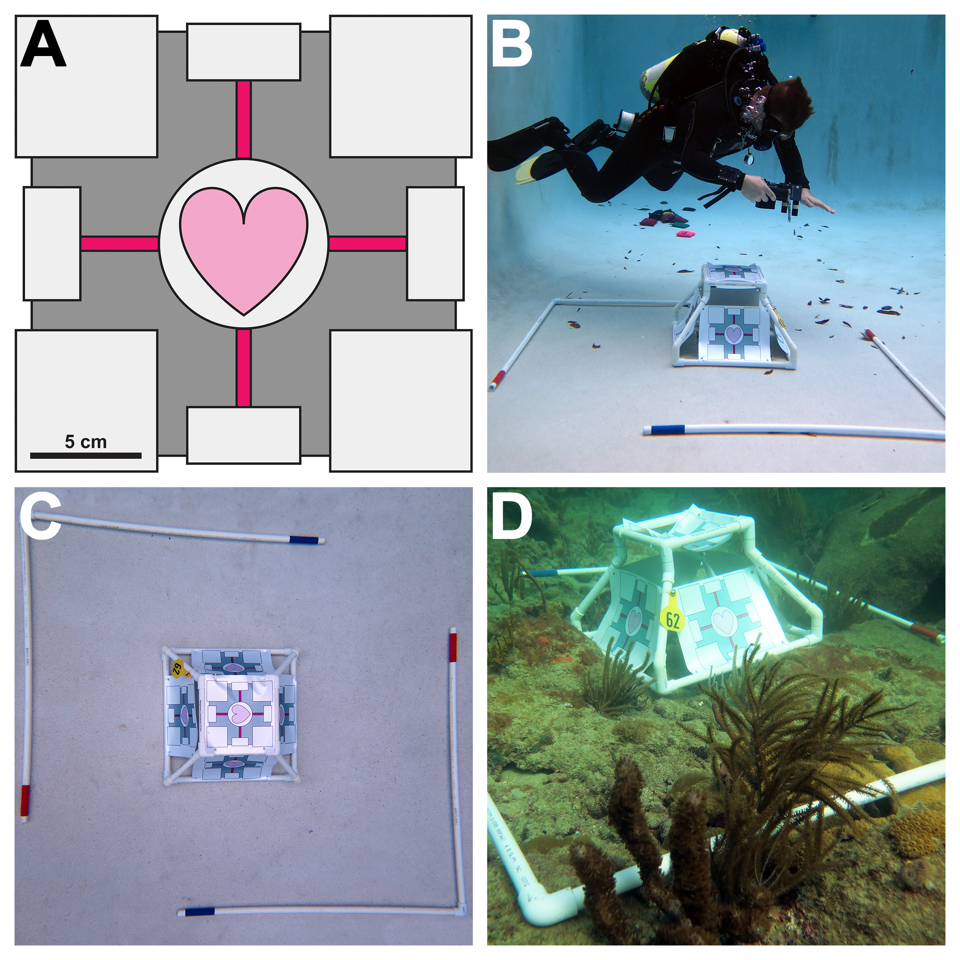

Supplement: S1 Fig — A) Template used for five sides of prism, scaled to produce replicates of three standardized shapes: square (40.32 cm2), rectangle (12.90 cm2), and circle (45.60 cm2) simulating surface area measurements on a coral colony. B) Deployment of prism and scaling frames with diver recording continuous video in a lawnmower-pattern at an approximate distance of 1 m. C) Overhead view of prism and scaling frames, with 10 cm banded tape for scaling. D) Deployment of prism and scaling frames on a reef environment at Lauderdale-by-the-Sea, FL. (TIF) [file pone.0252593.s001.tif]

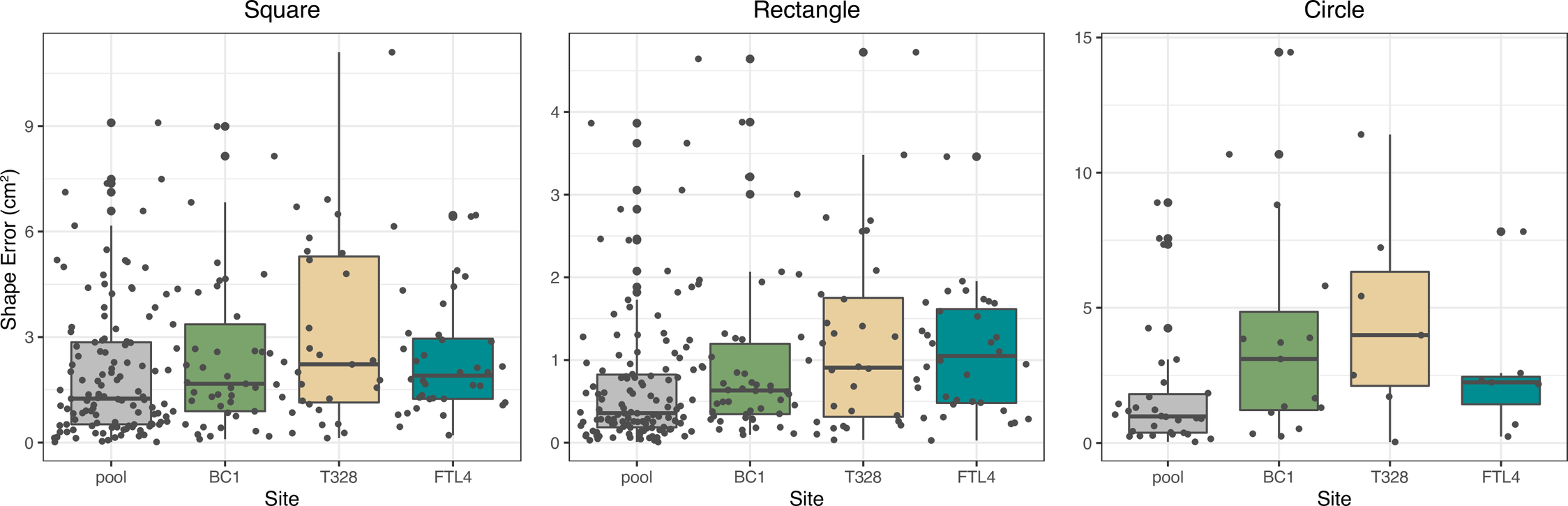

Supplement: S2 Fig — (TIF) [file pone.0252593.s002.tif]
