# Supplementary material for: Quantifying impacts of stony coral tissue loss disease on corals in Southeast Florida through surveys and 3D photogrammetry
Source: PLoS One. 2021 Jun 25;16(6):e0252593. doi: 10.1371/journal.pone.0252593 (PMC8232449; doi:10.1371/journal.pone.0252593)
Supplement: S1 Table — Non-significant p values are listed as “ns”. (DOCX) [file pone.0252593.s003.docx]

| **Data** | **Test** | **Comparison** | **Test Statistic** | ***p*-value** |
| --- | --- | --- | --- | --- |
| Rate of Tissue Loss | Kruskal-Wallis | T1 v T2 | 3.07 | ns |
|  |  | T2 v T3 | 1.97 | ns |
|  |  | T3 v T4 | 2.65 | ns |
