# Supplementary material for: Quantifying impacts of stony coral tissue loss disease on corals in Southeast Florida through surveys and 3D photogrammetry
Source: PLoS One. 2021 Jun 25;16(6):e0252593. doi: 10.1371/journal.pone.0252593 (PMC8232449; doi:10.1371/journal.pone.0252593)
Supplement: Attachment — Submitted filename: PONE-D-20-25422_reviewer.pdf [file pone.0252593.s007.pdf]

PLOS ONE

Quantifying impacts of stony coral tissue loss disease on corals in Southeast Florida through surveys and 3D photogrammetry

--Manuscript Draft--

**Manuscript Number:**	PONE-D-20-25422
**Article Type:**	Research Article
**Full Title:**	Quantifying impacts of stony coral tissue loss disease on corals in Southeast Florida through surveys and 3D photogrammetry
**Short Title:**	Impacts of stony coral tissue loss disease on corals through surveys and 3D photogrammetry
**Corresponding Author:**	Ian Robert Combs, M.Sc<br>Harbor Branch Oceanographic Institute<br>Fort Pierce, FL UNITED STATES
**Keywords:**	stony coral tissue loss disease; 3D photogrammetry; photogrammetry; coral health and disease; coral reef ecology; coral; Florida Reef Tract; coral reef ecosystems; Southeast Florida; 3D modeling; SCTLD; Caribbean coral reefs; Tropical Western Atlantic
**Abstract:**	Since 2014, stony coral tissue loss disease (SCTLD) has contributed to substantial declines of reef-building corals in Florida. The emergence of this disease, with its ability to impact more than 20 scleractinian coral species, has generated a need for both widespread reef monitoring and the implementation of novel survey and disease mitigation strategies. This study paired SCTLD prevalence assessments with colony-level monitoring to help improve understanding of disease dynamics on both individual coral colonies and reef-wide scales. Benthic surveys were conducted throughout the northern Florida Reef Tract to monitor the presence/absence of disease, disease prevalence, and coral species affected by SCTLD. Observed SCTLD prevalence was lower in Palm Beach than in Broward or Martin Counties, but there were no significant changes in prevalence over time. To assess colony-level impacts of the disease, we optimized a low-cost, rapid 3D photogrammetry technique in order to fate-track infected Montastraea cavernosa coral colonies over four time points spanning over three months. Total colony area and healthy tissue area on fate-tracked colonies decreased significantly over time, but disease lesion area did not, and was not correlated with total colony size. These results suggest that SCTLD affects colonies regardless of size, therefore larger colonies should be prioritized for colony-level intervention efforts to maximize success. Traditional coral surveys combined with 3D photogrammetry can provide greater insights into the spatial/temporal dynamics and impacts of coral diseases on individual colonies and coral communities than surveys or visual estimates of disease progression alone.
**Order of Authors:**	Ian Robert Combs, M.Sc
	Michael S Studivan
	Ryan J Eckert
	Joshua D Voss
**Additional Information:**	
**Question**	**Response**
**Financial Disclosure**<br><br>Enter a financial disclosure statement that describes the sources of funding for the work included in this submission. Review the submission guidelines for detailed requirements. View published research articles from *PLOS ONE* for specific	Funding for this research was awarded to J. Voss from the Florida Department of Environmental Protection, Environmental Protection Agency (South Florida Geographic Initiative award X7 00D667-17 with J. Beal as co-investigator, https://floridadep.gov/), and the NOAA Coral Reef Conservation Program (award NA16NOS4820052, https://coralreef.noaa.gov/). Additional funding was awarded to I. Combs by the Harbor Branch Oceanographic Institute Foundation through the Indian River Lagoon Graduate Research Fellowship (https://hboifoundation.org/).

Powered by Editorial Manager® and ProduXion Manager® from Aries Systems Corporation

examples.<br><br>This statement is required for submission and **will appear in the published article** if the submission is accepted. Please make sure it is accurate.<br><br>**Unfunded studies**<br>Enter: *The author(s) received no specific funding for this work.*<br><br>**Funded studies**<br>Enter a statement with the following details:<br>• Initials of the authors who received each award<br>• Grant numbers awarded to each author<br>• The full name of each funder<br>• URL of each funder website<br>• Did the sponsors or funders play any role in the study design, data collection and analysis, decision to publish, or preparation of the manuscript?<br>• **NO** - Include this sentence at the end of your statement: *The funders had no role in study design, data collection and analysis, decision to publish, or preparation of the manuscript.*<br>• **YES** - Specify the role(s) played.<br><br>* typeset	
**Competing Interests**<br><br>Use the instructions below to enter a competing interest statement for this submission. On behalf of all authors, disclose any competing interests that could be perceived to bias this work—acknowledging all financial support and any other relevant financial or non-financial competing interests.<br><br>This statement **will appear in the published article** if the submission is accepted. Please make sure it is accurate. View published research articles from *PLOS ONE* for specific examples.	The authors have declared that no competing interests exist.

**NO authors have competing interests**<br><br>Enter: *The authors have declared that no competing interests exist.*<br><br>**Authors with competing interests**<br><br>Enter competing interest details beginning with this statement:<br><br>*I have read the journal's policy and the authors of this manuscript have the following competing interests: [insert competing interests here]*<br><br>* typeset	
**Ethics Statement**<br><br>Enter an ethics statement for this submission. This statement is required if the study involved:<br><br>• Human participants<br>• Human specimens or tissue<br>• Vertebrate animals or cephalopods<br>• Vertebrate embryos or tissues<br>• Field research<br><br>Write "N/A" if the submission does not require an ethics statement.<br><br>General guidance is provided below. Consult the submission guidelines for detailed instructions. **Make sure that all information entered here is included in the Methods section of the manuscript.**	St. Lucie Inlet State Park Permit 06261715<br><br>FWC SAL-17-1960-SCRP

Powered by Editorial Manager® and ProduXion Manager® from Aries Systems Corporation

**Format for specific study types**<br><br>**Human Subject Research (involving human participants and/or tissue)**<br>• Give the name of the institutional review board or ethics committee that approved the study<br>• Include the approval number and/or a statement indicating approval of this research<br>• Indicate the form of consent obtained (written/oral) or the reason that consent was not obtained (e.g. the data were analyzed anonymously)<br><br>**Animal Research (involving vertebrate animals, embryos or tissues)**<br>• Provide the name of the Institutional Animal Care and Use Committee (IACUC) or other relevant ethics board that reviewed the study protocol, and indicate whether they approved this research or granted a formal waiver of ethical approval<br>• Include an approval number if one was obtained<br>• If the study involved *non-human primates*, add *additional details* about animal welfare and steps taken to ameliorate suffering<br>• If anesthesia, euthanasia, or any kind of animal sacrifice is part of the study, include briefly which substances and/or methods were applied<br><br>**Field Research**<br><br>Include the following details if this study involves the collection of plant, animal, or other materials from a natural setting:<br>• Field permit number<br>• Name of the institution or relevant body that granted permission	
**Data Availability**<br><br>Authors are required to make all data underlying the findings described fully available, without restriction, and from the time of publication. PLOS allows rare exceptions to address legal and ethical concerns. See the PLOS Data Policy and FAQ for detailed information.	Yes - all data are fully available without restriction

Powered by Editorial Manager® and ProduXion Manager® from Aries Systems Corporation

A Data Availability Statement describing where the data can be found is required at submission. Your answers to this question constitute the Data Availability Statement and **will be published in the article**, if accepted.<br><br>**Important:** Stating 'data available on request from the author' is not sufficient. If your data are only available upon request, select 'No' for the first question and explain your exceptional situation in the text box.<br><br>Do the authors confirm that all data underlying the findings described in their manuscript are fully available without restriction?	
**Describe where the data may be found in full sentences. If you are copying our sample text, replace any instances of XXX with the appropriate details.**<br><br>• If the data are **held or will be held in a public repository**, include URLs, accession numbers or DOIs. If this information will only be available after acceptance, indicate this by ticking the box below. For example: *All XXX files are available from the XXX database (accession number(s) XXX, XXX.)*.<br>• If the data are all contained **within the manuscript and/or Supporting Information files**, enter the following: *All relevant data are within the manuscript and its Supporting Information files.*<br>• If neither of these applies but you are able to provide **details of access elsewhere**, with or without limitations, please do so. For example:<br><br>*Data cannot be shared publicly because of [XXX]. Data are available from the XXX Institutional Data Access / Ethics Committee (contact via XXX) for researchers who meet the criteria for access to confidential data.*<br><br>*The data underlying the results presented in the study are available from (include the name of the third party*	All relevant data are within the manuscript and its Supporting Information files.<br><br>All data files, protocols, analyses pipelines and scripts can be found in our GitHub repository (https://github.com/icombs2017/analysisOf3dModels)

and contact information or URL).<br>• This text is appropriate if the data are owned by a third party and authors do not have permission to share the data.<br><br>* typeset	
Additional data availability information:	Tick here if the URLs/accession numbers/DOIs will be available only after acceptance of the manuscript for publication so that we can ensure their inclusion before publication.

Powered by Editorial Manager® and ProduXion Manager® from Aries Systems Corporation

Manuscript Click here to access/download;Manuscript;Manuscript.docx

Quantifying impacts of stony coral tissue loss disease on corals in Southeast Florida through surveys and 3D photogrammetry

Ian R Combs[1,2*], Michael S Studivan[1,3], Ryan J Eckert[1], Joshua D Voss[1]

[1] Department of Biological Sciences, Harbor Branch Oceanographic Institute, Florida Atlantic University, Fort Pierce, Florida, United States of America

[2] Current address: Elizabeth Moore International Center for Coral Reef Research & Restoration, Mote Marine Laboratory, Summerland Key, Florida, United States of America

[3] Current address: Cooperative Institute for Marine and Atmospheric Studies, University of Miami, Miami, Florida, United States of America

* combsi9892@gmail.com

Abstract

Since 2014, stony coral tissue loss disease (SCTLD) has contributed to substantial declines of reef-building corals in Florida. The emergence of this disease, with its ability to impact more than 20 scleractinian coral species, has generated a need for both widespread reef monitoring and the implementation of novel survey and disease mitigation strategies. This study paired SCTLD prevalence assessments with colony-level monitoring to help improve understanding of disease dynamics on both individual coral colonies and reef-wide scales. Benthic surveys were conducted throughout the northern Florida Reef Tract to monitor the presence/absence of disease, disease prevalence, and coral species affected by SCTLD. Observed SCTLD prevalence was lower in Palm Beach than in Broward or Martin Counties, but there were no significant changes ~~in prevalence~~ over time. To assess colony-level impacts of the disease, we optimized a low-cost, rapid 3D photogrammetry technique in order to fate-track infected *Montastraea cavernosa* coral colonies over four time points spanning over three months. Total colony area and healthy tissue area on fate-tracked colonies decreased significantly over time, but disease lesion area did not, and was not correlated with total colony size. These results suggest that SCTLD affects colonies regardless of size, therefore larger colonies should be prioritized for colony-level intervention efforts to maximize success. Traditional coral surveys combined with 3D photogrammetry can provide greater insights into the spatial/temporal dynamics and impacts of coral diseases on individual colonies and coral communities than surveys or visual estimates of disease progression alone.

Introduction

Coral cover in the Tropical Western Atlantic (TWA) has declined over the last four decades [1,2], and coral diseases have been identified as one major driver of widespread coral decline throughout the region [3]. In the 1990s, white band disease dramatically reduced coral cover of *Acropora cervicornis* and *Acropora palmata* by 95% [4]. In the Florida Keys, white pox was responsible for up to a 70% reduction in *A. palmata* cover in the late 1990s [5]. ~~Likewise,~~ increased disease prevalence following a coral bleaching event in 2005 caused a 60% decline in coral cover throughout the U.S. Virgin Islands [6].

Coral diseases, regardless of their host specificity, have contributed to the decline of many integral reef-building scleractinian species. While white pox, white band, and acute *Montipora* white syndrome are genus-specific, other disease have a broad, even pan-oceanic host range [6–8]. For example, white plague type II affects 17 species of scleractinian coral across multiple genera [9]. Widespread decreases in coral cover from diseases result in an ecological shift from diverse, coral-dominated communities to more homogenous, algae-dominated communities [10,11]. Such shifts have the potential to create long-term changes in fish assemblages and fishery yields [12] as well as the loss of key ecosystem services such as fisheries habitat [13], coastal wave protection [14] and nutrient cycling [15] that can persist long after a coral disease event subsides.

Since 2014, the Florida Reef Tract (FRT) has experienced an ongoing outbreak of a newly-described coral disease responsible for widespread coral mortality throughout ~~the FRT and now other regions of~~ the TWA. Stony coral tissue loss disease (SCTLD) is characterized as a highly virulent disease that affects over 20 species of scleractinian corals in the TWA [16]. SCTLD first appeared in the summer of 2014 following the dredging of Government Cut by the

Army Corps of Engineers in Miami-Dade County [17,18]. In subsequent years, reports of SCTLD infections have increased and spread from Miami-Dade County along the Florida Reef Tract (FRT) and into the wider TWA. To date, SCTLD has spread north to the northern terminus of the FRT in Martin County and south past the Marquesas Keys in Monroe County, with additional outbreaks observed in at least twelve territories throughout the TWA [19,20].

SCTLD manifests as lesions of necrotic tissue that spread across a colony, leaving behind denuded coral skeleton (Fig 1) [16]. SCTLD is histologically distinct from white plague, exhibiting a fast-acting, liquefactive necrosis after lesions developing deep within coral tissue and progress to the colony surface [21]. Across multiple host coral species, SCTLD affected colonies demonstrate altered microbial communities relative to their apparently healthy counterparts [22–24]. While both culture-based efforts and sequencing studies are ongoing, no pathogen for SCTLD has been identified to date.

Fig 1. SCTLD lesions on colony of *Montastraea cavernosa* (a) ~~Fate-tracked,~~ SCTLD-infected *Montastraea cavernosa* with (b) rendered 3D model, (c) characteristic disease lesion, and (d) necrotic tissue.

Traditionally, coral disease monitoring has relied on a combination of benthic survey methods and individual colony monitoring, the latter often requiring estimations of disease area, *in situ* linear measurements, or two-dimensional (2D) photographic assessments [25,26]. However underwater estimates and even 2D photographs can incorporate diver bias and inaccuracies [27]. Under water ~~three-dimensional (3D)~~ photogrammetry is an emerging technique that offers the potential to enhance both the accuracy and speed of data collection

when assessing coral disease impacts. Photogrammetry has been used in a number of marine applications including cetacean and elasmobranch identification and morphology [28,29], fish assemblage and habitat characterization [30,31], and growth measurements in coral [32,33]. 3D imaging approaches have principally relied on aerial and close-range digital photogrammetry, 3D scanners, and soft-copy (digital) photogrammetry [34–36]. However, these methods require known 3D locations and positions from which camera positions and orientation are derived [37]. Recently, structure-from-motion (SfM) photogrammetry has emerged as a powerful new approach that derives 3D structure from a series of overlapping images, similar to established stereoscopic methods. Rather than requiring known *a priori* positions, SfM orientation is resolved based on common features extracted from overlapping images [37]. Conveniently, this can be done using a moving sensor, or from still images generated by an individual with a camera. The emergence of SfM photogrammetry coupled with the decreasing costs of related software and image-capture equipment has made SfM photogrammetry an increasingly popular tool among biologists. On coral reefs, SfM photogrammetry has been used mainly for large scale habitat complexity, fractal dimension, and rugosity characterization [38,39], but few studies in coral reef systems have utilized SfM photogrammetry at the organismal level [40].

In this study we optimized and implemented a 3D photogrammetry technique designed for rapid data collection and analysis to understand the colony-level dynamics of SCTLD on fate-tracked *Montastraea cavernosa* colonies in Southeast Florida. The study was designed to provide insight into colony- and community-level dynamics of this poorly understood disease by combining traditional surveys with colony fate-tracking using 3D photogrammetry. The ultimate goal is application of this new method to improve the design, implementation, and success of intervention, mitigation, and management strategies.

Methods

Disease prevalence surveys

Four locations across the northern Florida Reef Tract (NFRT) were selected for disease surveys: St. Lucie Reef, Jupiter, Palm Beach, and Lauderdale-by-the-Sea (Fig 2), ~~these were areas of known, vulnerable coral communities with the goal of finding sufficient SCTLD incidence for subsequent colony fate-tracking analyses.~~ St. Lucie Reef is located in Martin County, Jupiter and Palm Beach are both located in Palm Beach County, and Lauderdale-by-the-Sea is located in Broward County. Following Hurricane Irma in September 2017, a rapid-response damage and disease survey effort was completed throughout Southeast Florida [41]. The ~~resulting~~ data from these initial surveys were used to ~~inform decisions of which sites within the NFRT to target for continued monitoring and fate-tracking~~ (Fig 2).

Fig 2. Map of study locations throughout the Northern Florida Reef Tract Florida
Red circles indicate roving diver survey sites and red triangles indicate sites where both roving diver surveys and coral fate-tracking occurred.

Roving diver disease surveys were conducted approximately monthly November 2017 to June 2019. These surveys were designed to assess the greatest reef area possible, quantifying disease prevalence over an estimated range of 100–2000 m^2 per survey based on ~~conditions, principally~~ underwater visibility. SCUBA divers swam for 20 min and recorded the species and disease status of every living coral colony ≥ 10 cm in diameter. Paling, partial bleaching, and bleaching were also noted ~~within surveys~~. From those data, SCTLD abundance and prevalence, species diversity, and species richness were calculated. Statistical tests were run in the R statistical environment [42]. Datasets were non-normal and normal distributions could not be

achieved through transformation; therefore, non-parametric tests were implemented for all analyses unless otherwise noted. Permutational analysis of variance (PERMANOVA; 9999 permutations) was used in the package vegan [43] to assess variation in disease prevalence among sites and survey times, with Bonferroni corrected pairwise comparisons using the package *pairwiseAdonis* [44].

Fate-Tracking of SCTLD affected *M. cavernosa*

Benthic survey data indicated that disease incidence was too low at Jupiter and Palm Beach sites, and that coral abundance was too low at sites in St. Lucie Reef, for statistically robust fate-tracking study in these locations. Three fate-tracking sites (T328, BC1, and FTL4) were established in Lauderdale-by-the-Sea ~~where sufficient disease incidence and coral abundance were present~~. These three sites are ~12 km from the nearby Hillsboro Inlet, less than 500 m from shore, and have been previously used for benthic and coral monitoring and surveys [45–47] ~~within the NFRT~~ (Fig 2). *Montastraea cavernosa* was selected for colony fate-tracking due to the abundance of infected colonies within the study sites. This coral species is considered intermediately susceptible to SCTLD, with onset of tissue loss occurring weeks to months later than highly susceptible species (e.g. *Dendrogyra cylindrus*, *Meandrina meandrites*, *Colpophyllia natans*). Lesions on infected *M. cavernosa* generally progress slower than highly susceptible species, with mortality occurring within months to years [16]. *Montastraea cavernosa* constituted approximately 10% of the reported cases of SCTLD in the NFRT ~~during post-Hurricane Irma disease and breakage assessments~~ [41].

Twenty-four colonies of *M. cavernosa* affected with SCTLD were tagged with individually numbered cattle tags across the three sites ~~(T328, BC1, FTL4; Fig 2). Sites were~~

visited four times in 2018: 24-Aug-2018 (T_1), 11-Sep-2018 (T_2), 8-Nov-2018 (T_3), and 17-Dec-2018 (T_4). *In situ* observations made for each colony included the presence/absence of disease, the number of disease lesions, and diver-based *in situ* estimates of percent mortality. Photographs were taken of each colony along with continuous video for 3D model generation to quantitatively measure total colony surface area and disease lesion area for each timepoint.

Fate-tracked colonies were filmed using methods outlined in Young et al. [39], with the following modifications: Canon G16 cameras in Fantasea underwater housings were set on "Underwater mode," 1080p and 60 frames per second (fps), and exposure was adjusted as needed based on ambient light conditions. One-meter, L-shaped PVC scale bars marked at 10 cm increments were placed at opposing right angles to frame the designated colony. A SCUBA diver maintained approximately 1 m altitude above the highest point of each coral colony and recorded continuous video while swimming repeated linear, parallel, adjacent passes in a lawnmower pattern with the camera pointed directly downward. The number of adjacent passes varied depending on colony size, with 60–70% overlap between passes to aid in downstream model generation. The camera was rotated 90° at the end of the first set of adjacent passes, then another set of passes was completed perpendicular to the first set. The two complete sets of passes for a single colony required between 1–3 mi[illegible]epending on colony size. Each sampling event produced an average of 14 GB of .mp4 video files, or approximately 425 MB of video files per coral colony.

Video processing and 3D model generation protocols are described in full in our GitHub repository (https://github.com/icombs2017/analysisOf3dModels). In summary, the free software package, *FFmpeg* (www.ffmpeg.org),was used to extract still frame images from videos of the fate-tracked colonies at a rate of 3 fps. Still images were then imported into AgiSoft Metashape

(AgiSoft LLC) software, which uses a proprietary algorithm that incorporates SfM and Brown's lens distortion model to generate 3D models from 2D images [37,48]. Model generation in Metashape was conducted according to the manufacturer's protocol in four general steps: 1) camera alignment, 2) dense point cloud generation, 3) mesh generation, and 4) texture overlay. Models were rendered on a 2018 Apple MacBook Pro with a 2.9 GHz processor, 16GB of RAM and a Radeon Pro Vega 16 4GB graphics card. A single model took approximately 40 min to render depending on the number of still images generated. Generated models were then exported as an .obj file and imported into the software Rhinoceros 3D (Robert McNeel & Associates) for analysis; the mean model file size was 64 MB.

Models were scaled using the PVC scale bars, then total colony surface area and disease lesion surface area were measured ~~with Rhinoceros 3D [39], where total colony area included both healthy and diseased coral tissue.~~ SCTLD disease lesion area was considered as the stark white coral tissue, and both total colony area and disease lesion area were generated directly within Rhinoceros 3D, while healthy tissue area was calculated by subtracting disease lesion area from total colony area. Rate of tissue loss per week and change in disease tissue area per week were calculated for each pair of timepoints. Additionally, lesion count was derived from the number of polygons created when tracing the disease lesion area. To identify significant effects of time on healthy area, diseased area, total area, rate of tissue loss, and change in disease tissue area, Friedman's rank sum tests were conducted using the package *PMCMRplus* [50]. Pairwise comparisons were made with Nemenyi tests in the package *PMCMR* [51]. Additionally, Spearman's rank correlation analyses were used to compare diseased area and total colony area, lesion count and disease area, lesion count and total colony area, and rate of tissue loss and total colony area.

3D model validation

Accuracy of model-generated surface area metrics was assessed by a validation experiment using a standardized mock coral colony. A square cupola prism with equal dimensions and surface features was constructed to represent a coral colony using ½" PVC and internal steel rebar to achieve negative buoyancy. The prism was constructed using a top square of 25.4 x 25.4 cm, a bottom square of 47.6 x 47.6 cm, and height of 30.5 cm (S1 Fig). Angled sides were achieved using 45° PVC tees to better represent a mounding coral colony. Additionally, polygons of known surface areas (square: 40.32 cm^2; rectangle: 12.90 cm^2; circle: 45.60 cm^2) were designed using graphics editing software, printed on waterproof paper, and affixed to the top and sloped sides of the weighted prism.

The prism was 3D modeled in a pool and at two reef sites (St. Lucie Reef and Lauderdale-by-the-Sea; Fig 2) to emulate variable water quality and benthic community parameters that may influence model generation. Nine replicate videos were captured of the prism and boundary frames in each of the three locations. The pool depth was 2.75 m, and reef locations ranged from 3.96–10.05 m at St. Lucie Reef, and 5.18–8.84 m at Lauderdale-by-the-Sea. Site locations were selected based on proximity to existing monitoring and fate-tracking efforts and represented two different bottom types characteristic of reef habitats in the NFRT (wormrock at St. Lucie Reef, and reef carbonate at Lauderdale-by-the-Sea). Additionally, benthic communities varied between the two reef locations from sparsely-populated scleractinian and turf algae communities, to gorgonian and scleractinian dominated communities, respectively.

3D models were generated and analyzed using the same protocols described earlier. Differences in overall model areas among locations were assessed using a single-factor ANOVA.

Surface area and error measurements were found to be highly skewed, so a square-root transformation was applied prior to statistical analyses. Non-parametric Kruskal-Wallis tests were run for each prism template shape (square, rectangle, circle) to determine whether surface area measurements differed by location. Pairwise comparisons were made using Dunn's tests for each of the prism shapes using the R package *FSA* [52]. Multivariate homogeneity of variance was first tested using the *betadisper* function of the package *vegan*, then a three-factor nested PERMANOVA was conducted across location, model, and prism face using the *adonis* function of the package *vegan*. Pairwise PERMANOVA tested for significant differences among pairwise comparisons of each factor using the package *pairwiseAdonis*.

To assess ~~variation in shape surface area measurements both within and among models~~, shape error was calculated as the absolute difference between measured shape surface areas and the corresponding template area. The error dataset was then analyzed using the same statistical tests as described above, except that a subset dataset was created which did not include the template values. Lastly, correlation analyses were conducted to determine if overall model area was significantly related to each of the three shape's error measurements for all models. Correlation analyses utilized Spearman's rank correlation coefficients and were conducted for each of the three locations within each prism shape.

Additionally, a subset of four models from the colony fate-tracking dataset were extracted at three different frame rates (3–5 fps) across their respective timepoints to determine if frame rate affected model accuracy. A non-parametric Kruskal-Wallis test was used to determine if model size differed across the different extraction frequencies.

Results

Disease prevalence surveys

SCTLD prevalence varied significantly across county (~~PERMANOVA; $F_{2, 42}$ = 12.24, p < 0.001, Table 1~~), but not through time, (Table 1, Fig 3). Pairwise comparisons indicated that disease prevalence in Palm Beach County was lower than ~~both~~ Martin and Broward counties (~~pairwise PERMANOVA; Martin: F = 14.87, p < 0.002, Broward: F = 53.61, p < 0.001;~~ Table 1, Fig 3). Relatively lower levels of disease prevalence were observed in Palm Beach County from December 2017–December 2018, with increasing disease prevalence at nearly all sites in March 2019. Disease monitoring did not begin in Broward County until November 2018; however, an increase in disease prevalence was also observed in March 2019 (Fig 3). Additionally, the highest reported disease prevalence of 43% at site SLR North in Martin County was affected by low sample size; only 7 living corals were observed, 3 of which were diseased. The species with the highest disease prevalence with more than 10 observations were *Pseudodiploria clivosa* (36%, n = 47), *Agaricia agaricites* (10%, n = 108), *Dichocoenia stokesii* (9%, n =23), *Orbicella faveolata* (6%, n = 49) and *Montastraea cavernosa* (5%, n = 3241).

Table 1. Results from univariate-permutational analysis of variance (PERMANOVA) comparing SCTLD prevalence from the roving diver surveys across location and time. Pairwise comparisons from results of the univariate PERMANOVA across all three counties (Martin, Palm Beach, and Broward County).

Test	Comparison	*F*	*p* value*
PERMANOVA	County	12.24	< 0.001
	Time	1.34	ns

Test	Comparison	*F*	*p* value*
	County:Time	0.97	ns
Pairwise	Broward – Martin	0.11	ns
	Broward – Palm Beach	53.61	< 0.001
	Martin – Palm Beach	14.87	0.002

*Non-significant *p* values listed as "ns".

Fig 3. Mean SCTLD prevalence across all three counties (Broward, Martin, and Palm Beach Counties). Points represent means; bars represent standard error.

Fate-Tracking of SCTLD affected *M. cavernosa*

Friedman's rank sum tests in[illegible]ated a significant decrease in total colony area (m^2) and healthy tissue area over time (total: $df_{3,94}$, Friedman $\chi^2 = 48.56$, $p < 0.001$; healthy: $df_{3,94}$, Friedman $\chi^2 = 41.29$, $p < 0.001$; Table 2, Fig 4). The number of disease lesions per colony differed by site (Kruskal-Wallis, H = 17.08 p < 0.001, S1 Table), with T328 exhibiting more disease lesions per colony (9.57 ± 1.07) than both BC1 (4.31 ± 0.51) and FTL4 (4.65 ± 0.87; Dunn's test, both $p < 0.05$; S1 Table). Correlation analyses between surface area and lesion metrics identified no significant correlation between colony size and disease lesion area (Spearman's rank correlation $r_s = 0.15$, $p = 0.154$, Fig a in S3 Fig). There was, however, a positive correlation between colony lesion number and disease lesion area (Spearman's rank correlation, $r = 0.67$, $p < 0.001$, Fig c in S2 Fig), but no correlation between total colony size and the number of disease lesions (Spearman's rank correlation, $r_s = 0.18$, $p = 0.076$, Fig b in S2 Fig).

Table 2. Results of Friedman's rank sum tests comparing total colony area, disease tissue area and healthy tissue area across time, with pairwise comparisons made using Nemenyi tests.

**Test**	**Comparison**	**Test Statistic**	***p* value***
Total Colony Size	Friedman's	48.55	< 0.001
	$T_1 – T_2$	4.27	ns
	$T_1 – T_3$	7.59	< 0.001
	$T_1 – T_4$	9.01	< 0.001
	$T_2 – T_3$	3.32	ns
	$T_2 – T_4$	4.74	0.026
	$T_3 – T_4$	1.42	ns
Healthy Tissue Area	Friedman's	41.29	< 0.001
	$T_1 – T_2$	4.27	0.026
	$T_1 – T_3$	7.59	< 0.001
	$T_1 – T_4$	9.01	< 0.001
	$T_2 – T_3$	3.32	ns
	$T_2 – T_4$	4.74	ns
	$T_3 – T_4$	1.42	ns
Disease Lesion Area	Friedman's	14.02	0.003
	$T_1 – T_2$	4.27	ns
	$T_1 – T_3$	7.59	ns
	$T_1 – T_4$	9.01	ns
	$T_2 – T_3$	3.32	ns
	$T_2 – T_4$	4.74	0.012
	$T_3 – T_4$	1.42	ns

T_1, 24-Aug-2018; T_2, 11-Sep-2018; T_3, 8-Nov-2018; T_4, 17-Dec-2018.

*Non-significant p-values listed as "ns".

Fig 4. Mean tissue areas through time(a) mean total colony area (cm^2) (b) mean healthy tissue area (cm^2), and (c) mean disease lesion area each site and through time for fate-tracked *M. cavernosa* colonies. T_1, 24-Aug-2018; T_2, 11-Sep-2018; T_3, 8-Nov-2018; T_4, 17-Dec-2018.

The rate of change in disease area did not significantly differ over time (Friedman's rank sum te[illegible]t, $df_{2, 71}$, Friedman $\chi^2 = 2.58$, $p = 0.275$). Average tissue loss between T_1–T_2 was -98.17 ± 40.74 cm^2 wk^{-1}, -24.99 ± 7.70 cm^2 wk^{-1} between T_2–T_3, and -45.10 ± 19.64 cm^2 wk^{-1} between T_3–T_4 (Fig S4a). A Friedman's rank sum test revealed significant differences between rates of tissue loss through time ($df_{2,71}$, Friedman $\chi^2 = 6.25$, $p = 0.044$). Rate of tissue loss was not significantly different among sites between any timepoint (S2 Table), and the rate of tissue loss was never correlated with colony size between any timepoint (S3 Table, Fig b in S4 Fig).

3D model accuracy assessment

Overall model area differed significantly among locations, with Lauderdale-by-the-Sea and St. Lucie Reef models being larger than those generated in the pool ANOVA: $F_{2,18} = 18.21$, $p < 0.001$). Kruskal-Wallis tests determined that only rectangle areas varied among locations (S4 Table; S4 Fig; $p < 0.001$), which was driven by pairwise differences between pool models and both Lauderdale-by-the-Sea and St. Lucie Reef locations (all $p < 0.001$). Assessment of multivariate variance identified heterogeneous variance among locations, with the highest variance attributed to the location with the lowest sample size (St. Lucie Reef). The three-factor PERMANOVA identified that shape surface area did not vary across locations or face, but that model was a significant factor explaining 27.71% of the observed variance (S5 Table; $F_{16,309} = 8.09$, $p = 0.043$). Pairwise PERMANOVA results for shape area can be found in S5 Table.

Statistical analyses of shape errors, on the other hand, showed that location was a significant factor for all three shapes (S4 Table; S4 Fig; square: $p < 0.001$, rectangle: $p < 0.001$, circle: $p = 0.0063$). Results of the pairwise comparisons among locations can be found in S4

Table. Multivariate variance was heterogeneous among locations as with the shape area dataset. PERMANOVA found all main model and interaction terms to be significant factors (S5 Table; PERMANOVA; location: $F_{16,309} = 29.59$, $p < 0.001$; model: $F_{16,309} = 8.42$, $p < 0.001$; face: $F_{16,309} = 4.01$, $p = 0.0067$), with main terms explaining 39.08% of the model variance. Pairwise PERMANOVA results for shape error can be found in S5 Table. Since there was a significant difference in overall model area among locations, nonparametric correlation analyses were run to determine whether shape error increased with increasing model area. For each of the three prism shapes, only St. Lucie Reef models showed significant correlations (S4 Fig; square: $r_s = 0.53$, $p < 0.001$; rectangle: $r_s = 0.58$, $p < 0.001$; circle: $r_s = 0.64$, $p = 0.0056$), where shape error was higher with larger models.

To assess the effects of different frame rates used during model generation on colony-scale surface area measurements, a single-factor Kruskal-Wallis test indicated that frame rate had no significant impact on total colony area (Kruskal-Wallis, H = 0.12, $p = 0.94$). This finding confirmed that higher frame rates from the initial video can be used to rectify poorly constructed models without affecting downstream spatial analyses. To keep processing time as low as possible, however, stills were extracted at 3fps unless otherwise necessary.

Discussion

Disease prevalence surveys

SCTLD is a unique coral disease due to i[illegible]xtent, number of species affected, and rapid progression [16,17,53,54]. It has spread over 400 km along the FRT, and now has been observed in at least 12 other territories throughout the TWA [20]. In contrast, one of the most widespread

outbreaks of acute *Monitpora* white syndrome to affect the Hawaiian archipelago only spanned ~7.5 km throughout Kāne’ohe Bay [55]. Other diseases, such as the species-generalist white syndrome, have been observed over broader spatial extents (~1500 km) in the Great Barrier Reef [56], yet the full extent of SCTLD is likely not fully realized as new observations are being reported throughout the Caribbean [19,20]. It is also important to note that throughout the NFRT, SCTLD prevalence among the four monitoring sites was higher than typically observed background disease prevalence values (~1–3%) for the TWA [57]. Many sites had no observed disease at discrete time points, but disease prevalence observed in this study was as high as 45% at some sites. This is in conjunction with previously-reported prevalence values of more than 60% disease prevalence among other sites throughout the FRT [17], suggesting that SCTLD can be uniquely well-distributed yet locally infectious compared to other coral diseases. The lower prevalence values reported in this versus the latter study may be in part due to differences in species composition between the NFRT and southern regions of the FRT. The most highly abundant and susceptible species in previous studies [17,53] were comparatively absent within our NFRT surveys likely due to relative rarity and previous SCTLD impacts before our monitoring sites were established [53,58].

Disease prevalence significantly varied across site, which is consistent with survey data generated from post-Hurricane Irma disease assessments [41] and likely due to highly variable coral abundances across the monitoring locations. Sites with the highest disease prevalence in Martin County were characterized by relatively low overall coral abundance and species richness as compared to sites in Palm Beach and Broward Counties to the south. The global mean disease prevalence for the NFRT observed in this study was 6%, which is similar to prevalence after Hurricane Irma (Walker 2018) and in previous SCTLD survey efforts [53].

We observed an increase in disease prevalence during the spring of 2018 which was unexpected, as prevalence for other described coral diseases often increases during the summer months as water temperatures increase [59–62]. SCTLD prevalence does not appear to have a positive correlation with temperature [17,53] as has been observed for other coral diseases [60,63,64], but potential environmental cofactors that may drive SCTLD prevalence need to be examined further.

Fate-Tracking of SCTLD Affected *M. cavernosa*

At the colony level, disease progression was highly variable across fate-tracked *M. cavernosa* colonies near Lauderdale-by-the-Sea. As expected, total colony size and healthy tissue area decreased significantly over time, demonstrating the impact SCTLD can have on living coral cover over just a few months. Disease lesion area did not correlate with total colony size suggesting that larger colonies do not exhibit a higher proportion of diseased tissue as compared to smaller colonies. As SCTLD progresses on coral colonies, loss of healthy tissue appears to be a more important indicator of disease virulence, as opposed to change in disease lesion area. Importantly, however, rates of tissue loss did correlate with total colony size. Therefore, larger corals may have a more favorable time horizon for potential intervention actions that could prevent total colony mortality. Considering the level of effort required for SCTLD interventions [65], prioritizing interventions on larger colonies with sufficient areas of uninfected tissue remaining may result in a great preservation of overall coral cover. Moreover, since smaller infected colonies are more likely to succumb to SCTLD, it may be appropriate to cull affected colonies, removing them from the environment to prevent further SCTLD transmission. Targeting ~~relatively~~ larger coral colony size classes and conducting intervention during early

stages of infection ~~when possible~~ may both improve the likelihood of successfully preserving coral cover.

3D model generation as a fate-tracking method

This study tested a relatively inexpensive and accurate 3D model generation pipeline as a means to fate-track individual coral colonies without substantial increases in expense, time, or computational requirements compared to observational and photographic techniques. Using simple computational methods, surface areas of complex coral morphologies were calculated to generate more accurate data than previously established methods, such as two-dimensional surface area estimation using photo processing software [66] or *in situ* linear extension measurements [25,67,68]. Additionally, this 3D modeling technique was validated through the quantification of model error using a mock coral deployed in various underwater environments. While shape area did not vary across locations or position on the prism, there was significant variation among iterations of models within locations (and with respect to shape error), emphasizing the need for sufficient replication for both statistical robustness and model troubleshooting. There was 2.17 ± 0.11 cm^2 of error in measurements of shapes with known surface area affixed to the sloping sides and top of the prism, which is relatively minor at a colony-level scale as the measured error represents <0.1% colony size on average.

In this study colony size did not affect model accuracy, however, overall model size may be affected if water quality parameters or oceanographic conditions are a limiting factor to 3D model quality (i.e. in nearshore reef environments; S2 Fig). There were some instances where model generation was poor due to anomalies in the Metashape algorithm, inconsistent underwater filming, or most common, reduced water quality. Normally this could be rectified by extracting still images from the video at an increased frame rate to ensure higher overlap among

images. Notably, extracting at a higher fps improved poor models, but did not ~~disproportionately~~ affect accuracy of models that were sufficiently captured using three frames per minute.

Due to Metashape's proprietary algorithm, adjustments within the model generation process to rectify poor models are limited. Stable, high-resolution image collection is therefore integral to successful downstream model generation. Alternatives to the lawn-mower video path used in this study may be more effective but also more time-intensive, particularly for tall (>1 m), highly-rugose coral colonies. Discrete photographs could be taken instead of continuous video [40] or video could be taken while swimming a circular pattern around the coral at varying angles [69]. While the technique presented here was optimized for individual coral colonies, stereoscopic camera setups, with one wide lens and one zoom lens, may be more appropriate for reef-scale photogrammetry [70]. Critically, while disease monitoring via linear extension measurements may be ab[illegible]etermine potential differences among colonies or treatments over time [16,25,71], linear extension does not accurately quantify tissue loss and may underestimate the impacts of disease lesions on coral colonies. Therefore, 3D approaches, such as the method presented here, should be employed to accurately quantify lost coral surface area and to identify colonies for which intervention efforts may be successful.

Conclusions

SCTLD is particularly non-discriminant compared to other coral diseases with regard to its host specificity and seasonal distribution. Given this study's observations relative to tissue loss and coral colony sizes, interventions on smaller colonies with numerous active lesions may be inefficient and ultimately unsuccessful. Instead, attempts to mitigate the impacts of SCTLD at the colony level should consider 1) culling small colonies to prevent disease transmission and/or

2) shifting intervention focus toward larger, longer-lived colonies with more tissue remaining. Targeted and prioritized interventions may be particularly important in instances where time, personnel, weather opportunities, or boating and diving resources are limited. Similarly, since SCTLD is a progressive, necrotic infection, area of tissue loss may represent an alternative metric to quantify the severity of infection and disease progression, and therefore may be more informative than disease lesion area or percent of affected tissue. However, additional studies incorporating longer timescales, multiple species, and similar quantitative 3D metrics are warranted to confirm these patterns of disease progression across all susceptible species. Rapid, cost-effective, and accurate methods using 3D models for quantifying coral surface area are quite feasible so long as the appropriate redundancies and precautions are followed. It is recommended that managers and intervention specialists—particularly those focusing on SCTLD—adopt photogrammetric methods to ensure that data is accurate and comparable across future studies.

Data Availability

Model generation protocol, analysis scripts, and documentation are available on GitHub (https://github.com/icombs2017/analysisOf3dModels). Disease prevalence observations can be found in the S1 Dataset. Surface area measurements from the fate-tracked coral colonies can be found in the S2 Dataset. Surface area and error measurements for the three prism shapes can be found in S3 Dataset.

Acknowledgements

We thank J. Nelson and M. Roy for assistance with boating and diving operations; J. Beal, E. Shilling, and A. Sturm for assistance with data collection. K. Kerrigan, J. Walczak, B. Walker, and K. Neely provided valuable feedback on the study design.

References

1. Gardner TA, Côté IM, Gill JA, Grant A, Watkinson AR. Long-term region-wide declines in Caribbean corals. Science (80-). 2003;301: 958–960. doi:10.1126/science.1086050

2. Mumby PJ. The Impact of Exploiting Grazers (Scaridae) on the Dynamics of Caribbean Coral Reefs Author (s): Peter J . Mumby Published by : Wiley Stable URL : http://www.jstor.org/stable/40061693 Accessed : 05-04-2016 08 : 06 UTC Your use of the JSTOR archive ind. 2016;16: 747–769.

3. Richardson LL. Coral diseases: What is really known? Trends Ecol Evol. 1998;13: 438–443. doi:10.1016/S0169-5347(98)01460-8

4. Aronson RB, Precht WF. White-band disease and the changing face of Caribbean coral reefs. Hydrobiologia. 2001;460: 25–38. doi:10.1023/A:1013103928980

5. Patterson KL, Porter JW, Ritchie KB, Polson SW, Mueller E, Peters EC, et al. The etiology of white pox, a lethal disease of the Caribbean elkhorn coral, Acropora palmata. Proc Natl Acad Sci U S A. 2002;99: 8725–8730. doi:10.1073/pnas.092260099

6. Miller J, Muller E, Rogers C, Waara R, Atkinson A, Whelan KRT, et al. Coral disease following massive bleaching in 2005 causes 60% decline in coral cover on reefs in the US Virgin Islands. Coral Reefs. 2009;28: 925–937. doi:10.1007/s00338-009-0531-7

7. Nugues MM. Impact of a coral disease outbreak on coral communities in St Lucia. 2002;229: 61–71.

8. Miller MW, Williams DE. Coral disease outbreak at Navassa, a remote Caribbean island. Coral Reefs. 2007;26: 97–101. doi:10.1007/s00338-006-0165-y

9. Richardson LL, Goldberg WM, Carlton RG, Halas JC. Coral disease outbreak in the Florida Keys: Plague Type II. Rev Biol Trop. 1998;46: 187–198. Available:

http://www.amlc-carib.org/meetings/procs/1997AMLC_Proceedings/richardson.pdf

10. McClanahan TR, Muthiga NA. An ecological shift in a remote coral atoll of Belize over 25 years. Environ Conserv. 1998;25: 122–130. doi:10.1017/S0376892998000174

11. McManus JW, Polsenberg JF. Coral-algal phase shifts on coral reefs: Ecological and environmental aspects. Prog Oceanogr. 2004;60: 263–279. doi:10.1016/j.pocean.2004.02.014

12. Graham NAJ, Wilson SK, Jennings S, Polunin NVC, Robinson J, Bijoux JP, et al. Lag effects in the impacts of mass coral bleaching on coral reef fish, fisheries, and ecosystems. Conserv Biol. 2007;21: 1291–1300. doi:10.1111/j.1523-1739.2007.00754.x

13. Smith S V. Coral-reef area and the contributions of reefs to processes and resources of the world's oceans. Nature. 1978;273: 225–226. doi:10.1038/273225a0

14. Sheppard C, Dixon DJ, Gourlay M, Sheppard A, Payet R. Coral mortality increases wave energy reaching shores protected by reef flats: Examples from the Seychelles. Estuar Coast Shelf Sci. 2005;64: 223–234. doi:10.1016/j.ecss.2005.02.016

15. Yamamuro M, Kayanne H, Minagawao M. Carbon and nitrogen stable isotopes of primary producers in coral reef ecosystems. Limnol Oceanogr. 1995;40: 617–621. doi:10.4319/lo.1995.40.3.0617

16. NOAA. Stony Coral Tissue Loss Disease Case Definition. Florida Keys Natl Mar Sanctuary. Silver Spring, MD; 2018. Available: https://nmsfloridakeys.blob.core.windows.net/floridakeys-prod/media/docs/20181002-stony-coral-tissue-loss-disease-case-definition.pdf

17. Precht WF, Gintert BE, Robbart ML, Fura R, Van Woesik R. Unprecedented Disease-Related Coral Mortality in Southeastern Florida. Sci Rep. 2016;6: 31374.

doi:10.1038/srep31374

18. Cunning R, Silverstein RN, Barnes BB, Baker AC. Extensive coral mortality and critical habitat loss following dredging and their association with remotely-sensed sediment plumes. Mar Pollut Bull. 2019;145: 185–199. doi:10.1016/j.marpolbul.2019.05.027

19. Alvarez-Filip L, Estrada-Saldívar N, Pérez-Cervantes E, Molina-Hernández A, González-Barrios FJ. A rapid spread of the stony coral tissue loss disease outbreak in the Mexican Caribbean. PeerJ. 2019;2019. doi:10.7717/peerj.8069

20. Roth L, Kramer PR, Doyle E, O'Sullivan C. Caribbean SCTLD Dashboard. 2020 [cited 6 Apr 2020]. Available: www.agrra.org

21. Landsberg JH, Kiryu Y, Gray C, Wilson P, Perry N, Waters Y. MCAV disease lab analyses update. 2018.

22. Meyer JL, Castellanos-Gell J, Aeby GS, Häse CC, Ushijima B, Paul VJ. Microbial Community Shifts Associated With the Ongoing Stony Coral Tissue Loss Disease Outbreak on the Florida Reef Tract. Front Microbiol. 2019;10: 2244. doi:10.3389/fmicb.2019.02244

23. Rosales SM, Clark AS, Huebner LK, Ruzicka RR, Muller EM. Rhodobacterales and Rhizobiales Are Associated With Stony Coral Tissue Loss Disease and Its Suspected Sources of Transmission. Front Microbiol. 2020;11. doi:10.3389/fmicb.2020.00681

24. Aeby GS, Ushijima B, Campbell JE, Jones S, Williams GJ, Meyer JL, et al. Pathogenesis of a Tissue Loss Disease Affecting Multiple Species of Corals Along the Florida Reef Tract. Front Mar Sci. 2019;6: 678. doi:10.3389/fmars.2019.00678

25. Voss JD, Richardson LL. Nutrient enrichment enhances black band disease progression in corals. Coral Reefs. 2006;25: 569–576. doi:10.1007/s00338-006-0131-8

26. Dalton SJ, Smith SDA. Coral disease dynamics at a subtropical location, Solitary Islands Marine Park, eastern Australia. Coral Reefs. 2006;25: 37–45. doi:10.1007/s00338-005-0039-8

27. Courtney LA, Fisher WS, Raimondo S, Oliver LM, Davis WP. Estimating 3-dimensional colony surface area of field corals. J Exp Mar Bio Ecol. 2007;351: 234–242. doi:10.1016/j.jembe.2007.06.021

28. Webster T, Dawson S, Slooten E. A simple laser photogrammetry technique for measuring Hector's dolphins (Cephalorhynchus hectori) in the field. Mar Mammal Sci. 2010;26: 296–308. doi:10.1111/j.1748-7692.2009.00326.x

29. Rohner CA, Richardson AJ, Marshall AD, Weeks SJ, Pierce SJ. How large is the world's largest fish? Measuring whale sharks Rhincodon typus with laser photogrammetry. J Fish Biol. 2011;78: 378–385. doi:10.1111/j.1095-8649.2010.02861.x

30. Harborne AR, Mumby PJ, Ferrari R. The effectiveness of different meso-scale rugosity metrics for predicting intra-habitat variation in coral-reef fish assemblages. Environ Biol Fishes. 2012;94: 431–442. doi:10.1007/s10641-011-9956-2

31. Burns JHR, Delparte D, Gates RD, Takabayashi M. Integrating structure-from-motion photogrammetry with geospatial software as a novel technique for quantifying 3D ecological characteristics of coral reefs. PeerJ. 2015;2015: 1–19. doi:10.7717/peerj.1077

32. Schutter M, Crocker J, Paijmans A, Janse M, Osinga R, Verreth AJ, et al. The effect of different flow regimes on the growth and metabolic rates of the scleractinian coral Galaxea fascicularis. Coral Reefs. 2010;29: 737–748. doi:10.1007/s00338-010-0617-2

33. dela Cruz DW, Rinkevich B, Gomez ED, Yap HT. Assessing an abridged nursery phase for slow growing corals used in coral restoration. Ecol Eng. 2015;84: 408–415.

doi:10.1016/j.ecoleng.2015.09.042

34. Lane SN, James TD, Crowell MD. Application of digital photogrammetry to complex topography for geomorphological research. Photogramm Rec. 2000;16: 793–821. doi:10.1111/0031-868X.00152

35. Lohani B, Mason DC. Application of airborne scanning laser altimetry to the study of tidal channel geomorphology. ISPRS J Photogramm Remote Sens. 2001;56: 100–120. doi:10.1016/S0924-2716(01)00041-7

36. Klingler JH, Krüger MT, Kogias E, Brendecke SM, Hubbe U, Scheiwe C. Minimally invasive resection and vertebroplasty for an osteolytic C-1 metastasis of malignant meningioma: Case report. J Neurosurg Spine. 2015;23: 602–606. doi:10.3171/2015.1.SPINE141041

37. Westoby MJ, Brasington J, Glasser NF, Hambrey MJ, Reynolds JM. "Structure-from-Motion" photogrammetry: A low-cost, effective tool for geoscience applications. Geomorphology. 2012;179: 300–314. doi:10.1016/j.geomorph.2012.08.021

38. Leon JX, Roelfsema CM, Saunders MI, Phinn SR. Measuring coral reef terrain roughness using "Structure-from-Motion" close-range photogrammetry. Geomorphology. 2015;242: 21–28. doi:10.1016/j.geomorph.2015.01.030

39. Young GC, Dey S, Rogers AD, Exton D. Correction: Cost and time-effective method for multi-scale measures of rugosity, fractal dimension, and vector dispersion from coral reef 3D models (PLoS ONE (2018)12: 4 (e0175341) DOI : 10.1371/journal.pone.0175341). Metze K, editor. PLoS One. 2018;13: 1–18. doi:10.1371/journal.pone.0201847

40. Ferrari R, Figueira WF, Pratchett MS, Boube T, Adam A, Kobelkowsky-Vidrio T, et al. 3D photogrammetry quantifies growth and external erosion of individual coral colonies

and skeletons. Sci Rep. 2017;7: 1–9. doi:10.1038/s41598-017-16408-z

41. Walker BK. Southeast Florida reef-wide Post-Irma coral disease surveys. 2018. Available: https://floridadep.gov/sites/default/files/Southeast-Florida-Reef-Wide-Post-Irma-Coral-Disease-Surveys_0.pdf

42. 3.5.1. RDCT. A Language and Environment for Statistical Computing. R Foundation for Statistical Computing. Vienna, Austria; 2018. p. https://www.R-project.org. Available: http://www.r-project.org

43. Oksanen AJ, Blanchet FG, Kindt R, Legen- P, Minchin PR, Hara RBO, et al. Community Ecology Package. … Ecology Package …. 2012. p. 263. doi:10.4135/9781412971874.n145

44. Martinez Arbizu P. pairwiseAdonis: Pairwise multilevel comparison using adonis. R package version 0.4. 2020.

45. Gilliam DS, Walton CJ, Brinkhuis V, Ruzicka R, Colella M. Southeast Florida Coral Reef Evaluation and Monitoring Project 2014, Year 12. Florida DEP Report #RM085. 2015; 43. Available: https://floridadep.gov/sites/default/files/SECREMP_2014.pdf

46. Jones NP. Spatiotemporal change in the benthic community of southeast Florida. 2018; 1–60. Available: https://nsuworks.nova.edu/occ_stuetd

47. Walker BK, Brunelle A. Southeast Florida large (> 2 meter) diseased coral colony intervention summary report Florida Department of Environmental Protection Coral Reef Conservation Program Florida Fish and Wildlife Conservation Commission Southeast Florida large (> 2 meter). 2018.

48. Brown DC. Close-range camera calibration. Symp Close-Range Photogramm. 1971;37: 855–866. Available: https://www.asprs.org/wp-

content/uploads/pers/1971journal/aug/1971_aug_855-866.pdf

49. Agisoft LCC St.Peterburg - Russia. Agisoft Metashape User Manual Standrd Edition. 2019. p. 139. Available: https://www.agisoft.com/pdf/metashape-pro_1_5_en.pdf

50. Pohlert T. PMCMRplus: Calculate pairwise comparisons of mean rank sums. 2020. Available: https://cran.r-project.org/package=PMCMRplus

51. Pohlert T. The Pairwise Multiple Comparison of Mean Ranks Package (PMCMR). R package. 2014. p. 27. Available: http://cran.r-project.org/package=PMCMR

52. Ogle D, Wheeler P, Dinno A. FSA: Fisheries Stock Analysis. R package version 0.8.30. 2020. Available: https://github.com/droglenc/FSA.

53. Walton CJ, Hayes NK, Gilliam DS. Impacts of a regional, multi-year, multi-species coral disease outbreak in Southeast Florida. Front Mar Sci. 2018;5: 323. doi:10.3389/fmars.2018.00323

54. Nathan AJ, Scobell A. How China sees America. Foreign Aff. 2012. doi:10.1017/CBO9781107415324.004

55. Aeby GS, Callahan S, Cox EF, Runyon C, Smith A, Stanton FG, et al. Emerging coral diseases in Kāne’ohe Bay, O’ahu, Hawai’i (USA): Two major disease outbreaks of acute Montipora white syndrome. Dis Aquat Organ. 2016;119: 189–198. doi:10.3354/dao02996

56. Selig ER, Drew Harvell C, Bruno JF, Willis BL, Page CA, Casey KS, et al. Analyzing the relationship between ocean temperature anomalies and coral disease outbreaks at broad spatial scales. 2011; 111–128. doi:10.1029/61ce07

57. Weil E, Cróquer A. Spatial variability in distribution and prevalence of Caribbean scleractinian coral and octocoral diseases. I. Community-level analysis. Dis Aquat Organ. 2009;83: 195–208. doi:10.3354/dao02011

58. Beal J, Voss J, Edge S, Cohen L. Assessment of coral stressors on St. Lucie Reef: Florida's northernmost coral reef. Final Rep State Wildl Grant USFWS T-91-1. 2012. Available: http://fau.digital.flvc.org/islandora/object/fau%3A2737/datastream/OBJ/view/Assessment_of_coral_stressors_on_St__Lucie_reef__Florida___s_northernmost_coral_reef.pdf

59. Ben-Haim Y, Zicherman-Keren M, Rosenberg E. Temperature-regulated bleaching and lysis of the coral Pocillopora damicomis by the novel pathogen Vibrio coralliilyticus. Appl Environ Microbiol. 2003;69: 4236–4242. doi:10.1128/AEM.69.7.4236-4242.2003

60. Bruno JF, Selig ER, Casey KS, Page CA, Willis BL, Harvell CD, et al. Thermal stress and coral cover as drivers of coral disease outbreaks. Roberts C, editor. PLoS Biol. 2007;5: 1220–1227. doi:10.1371/journal.pbio.0050124

61. Voss JD, Richardson LL. Coral diseases near Lee Stocking Island, Bahamas: Patterns and potential drivers. Dis Aquat Organ. 2006;69: 33–40. doi:10.3354/dao069033

62. Zvuloni A, Artzy-Randrup Y, Katriel G, Loya Y, Stone L. Modeling the Impact of White-Plague Coral Disease in Climate Change Scenarios. PLoS Comput Biol. 2015;11: 1–22. doi:10.1371/journal.pcbi.1004151

63. Harvell DC, Jordán-Dahlgren E, Merkel S, Rosenberg E, Raymundo LJ, Smith GW, et al. Coral disease, environmental drivers, and the balance between coral and microbial associates. Oceanography. 2007;20: 172–195. doi:10.5670/oceanog.2007.91

64. Roberts KE, Hadfield JD, Sharma MD, Longdon B. Changes in temperature alter the potential outcomes of virus host shifts. PLoS Pathog. 2018;14: 1–16. doi:10.1371/journal.ppat.1007185

65. Neely K. Florida Keys Coral Disease STrike Team: FY 2019/2020 Final Report. Miami,

FL; 2020.

66. Schneider CA, Rasband WS, Eliceiri KW. NIH Image to ImageJ: 25 years of image analysis. Nat Methods. 2012;9: 671–675. doi:10.1038/nmeth.2089

67. Williams DE, Miller MW. Coral disease outbreak: Pattern, prevalence and transmission in Acropora cervicornis. Mar Ecol Prog Ser. 2005;301: 119–128. doi:10.3354/meps301119

68. Neely KL, Macaulay KA, Hower EK, Dobler MA. Effectiveness of topical antibiotics in treating corals affected by Stony Coral Tissue Loss Disease. PeerJ. 2020;8: e9289. doi:10.7717/peerj.9289

69. Figueira W, Ferrari R, Weatherby E, Porter A, Hawes S, Byrne M. Accuracy and precision of habitat structural complexity metrics derived from underwater photogrammetry. Remote Sens. 2015;7: 16883–16900. doi:10.3390/rs71215859

70. Sandin S, Zgliczynski B, Bonito L, Edwards C, Pedersen N, Sullivan C, et al. Large area imagery collection & processing standar operating procedures Version 2.0. 2019; 1–63.

71. Brandt ME, Smith TB, Correa AMS, Vega-Thurber R. Disturbance Driven Colony Fragmentation as a Driver of a Coral Disease Outbreak. Harder T, editor. PLoS One. 2013;8: 1–10. doi:10.1371/journal.pone.0057164

Supporting Information

S1 Table. Statistical outputs for single-factor Kruskal-Wallis and pairwise Dunn's tests for all three sites: BC1, T328, and FTL4. Non-significant *p* values listed as "ns".

S2 Table. Kruskal-Wallis test comparing rate of tissue loss and site. Non-significant *p* values are listed as "ns".

S3 Table. Spearman's rank correlation comparing rates of tissue loss with total colony size. Non-significant *p* values area listed as "ns".

S4 Table. Statistical outputs for single-factor Kruskal-Wallis and pairwise Dunn's tests for each prism shape (square, rectangle, circle) conducted for surface area and error datasets. Non-significant *p* values represented as ns.

S5 Table. Statistical outputs for multivariate main and pairwise PERMANOVAs across prism shapes (square, rectangle, circle) conducted for surface area and error datasets. Non-significant *p* values represented as ns, asterisks denote significant pairwise comparisons where main model terms were not significant, and NAs indicate where no pairwise comparisons were conducted.

S1 Fig. Panel of prism template and deployment for underwater 3D photogrammetry. A) Template used for five sides of prism, scaled to produce replicates of three standardized shapes: square (40.32 cm^2), rectangle (12.90 cm^2), and circle (45.60 cm^2) simulating surface area measurements on a coral colony. B) Deployment of prism and scaling frames with diver recording continuous video in a lawnmower-pattern at an approximate altitude of 1 m. C) Overhead view of prism and scaling frames, with 10 cm banded tape for scaling. D) Deployment of prism and scaling frames on a reef environment at Lauderdale-by-the-Sea, FL.

S2 Fig. Plot of mean change in tissue area on fate-tracked *M. cavernosa* colonies from pairwise time comparisons (T_1–T_2, T_2–T_3, T_3–T_4).

S3 Fig. Spearman's rank correlations between (a) disease lesion area and total colony area, (b) number of disease lesions and total colony area, and (c) number of disease lesions and disease lesion area. Significant correlation shown as trendline with 95% CI for (c).

S4 Fig. Panel of surface area and error measurements for each of the three prism shapes (square, rectangle, circle; columns). For shape surface areas (top row), the black lines indicate the template shape areas. Shape errors (middle row) were calculated as the absolute difference between measured shape surface areas and template areas. Overall *p* values represent results of single-factor Kruskal-Wallis tests comparing shape measurements across sites, and different letters denote significant pairwise comparisons between sites as generated by Dunn's tests. Shape errors were also correlated to overall model area (bottom row) using correlation analyses, with Spearman's rho (r_s) and *p* values given for each site.

S1 Dataset. Dataset containing disease prevalence data from roving diver disease surveys.

S2 Dataset. Dataset containing tissue area measurements from rendered 3D models of fate-tracked *Montastraea cavernosa* colonies.

S3 Dataset. Dataset containing model area, shape surface area, and shape error measurements from prism deployment across three sites (pool, Lauderdale-by-the-Sea, St. Lucie Reef). Error was calculated as the absolute difference between measured surface areas and actual area of the template shapes.

Fig 1

Click here to access/download;Figure;Fig1.tif

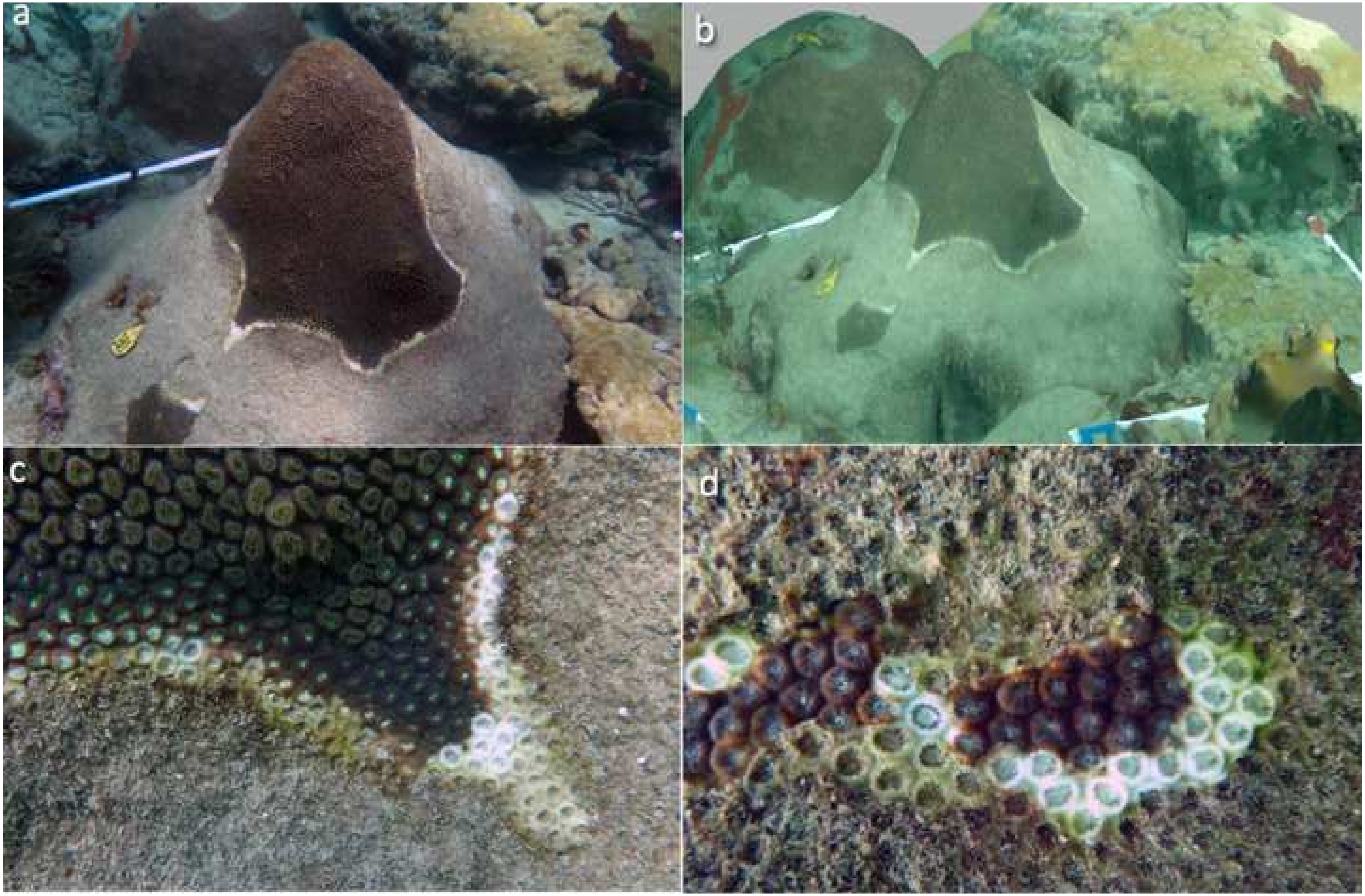

Fig2

Click here to access/download;Figure;Fig2.eps

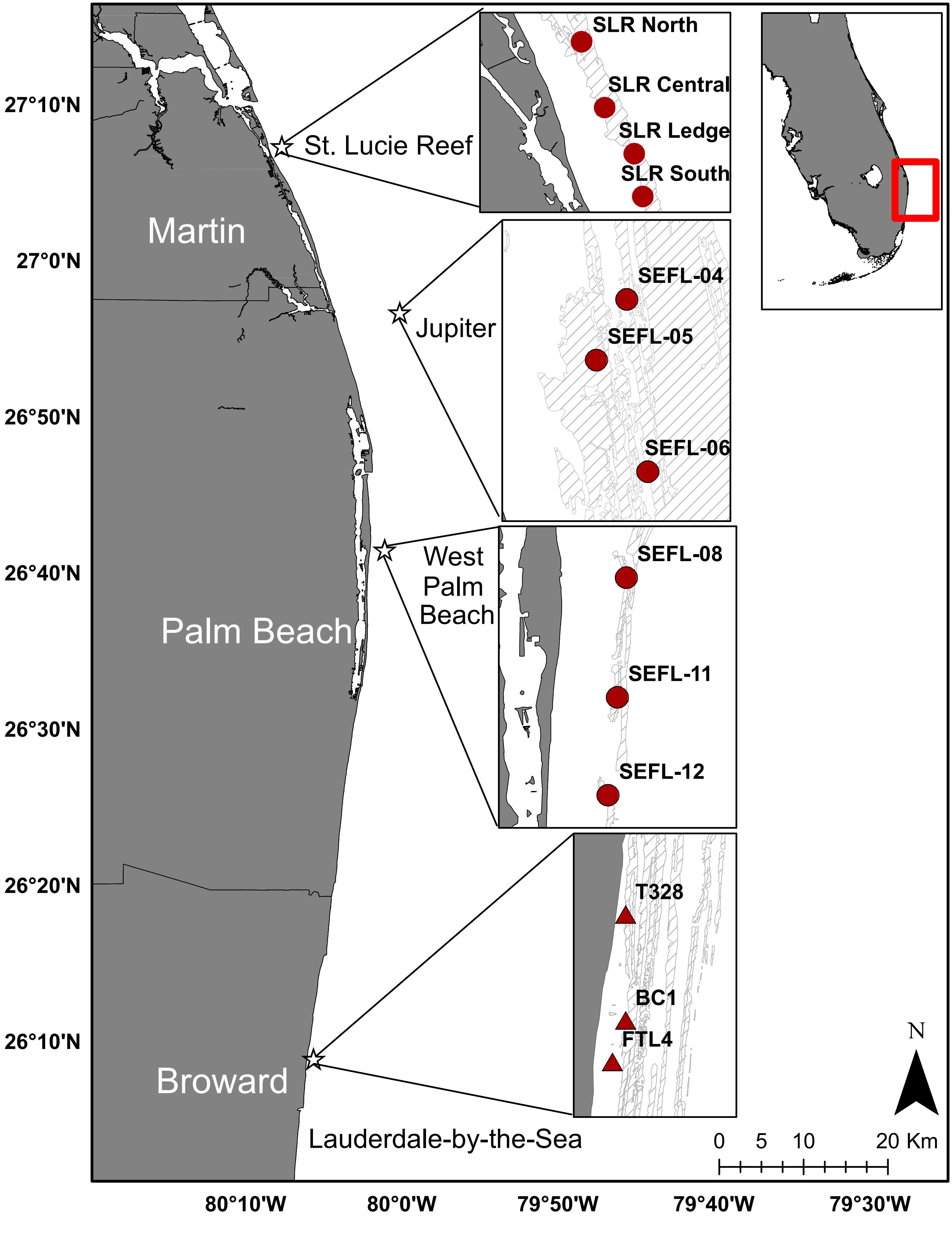

Click here to access/download;Figure;Fig3.png

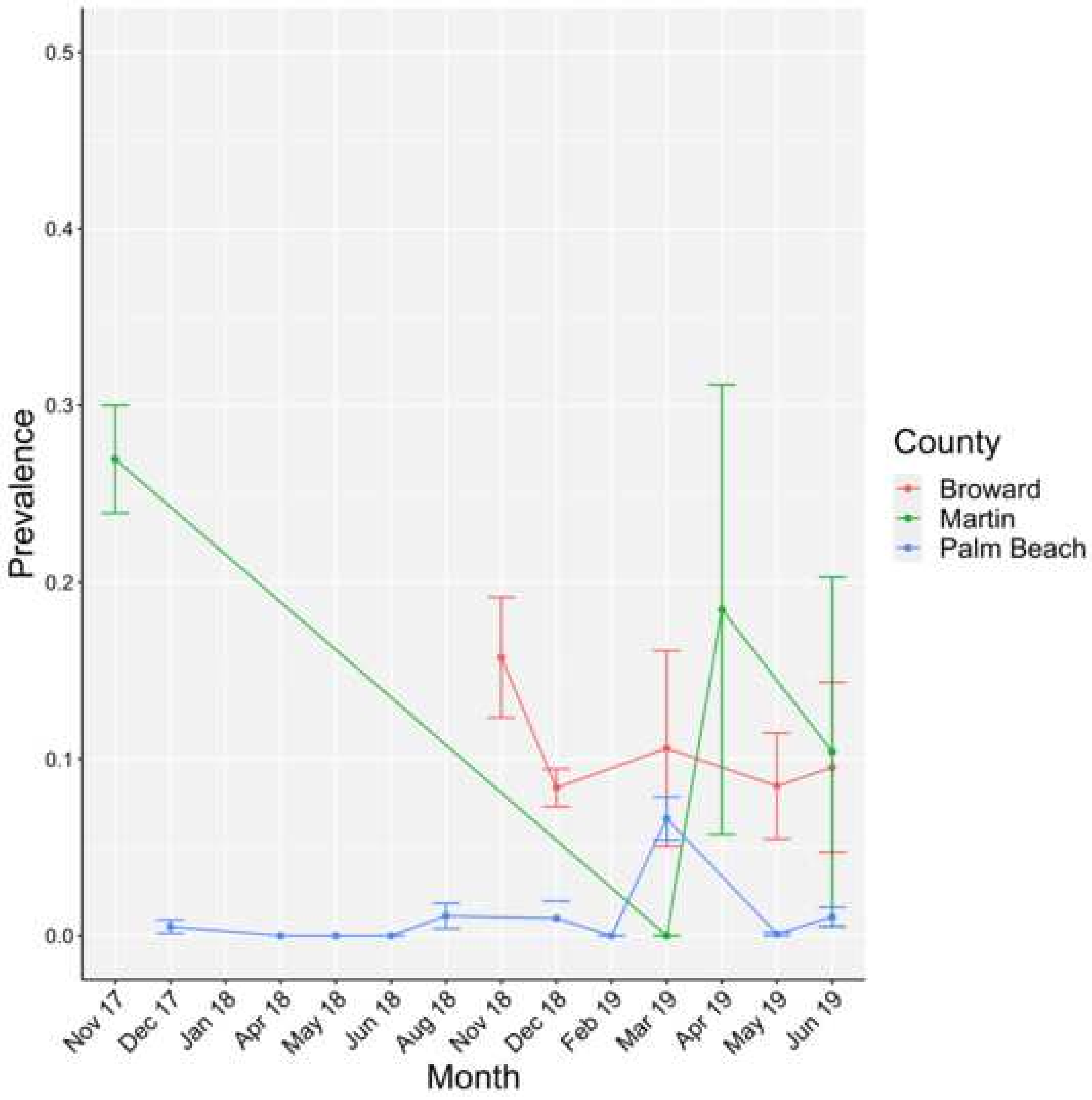

Fig4

Click here to access/download;Figure;Fig4.png

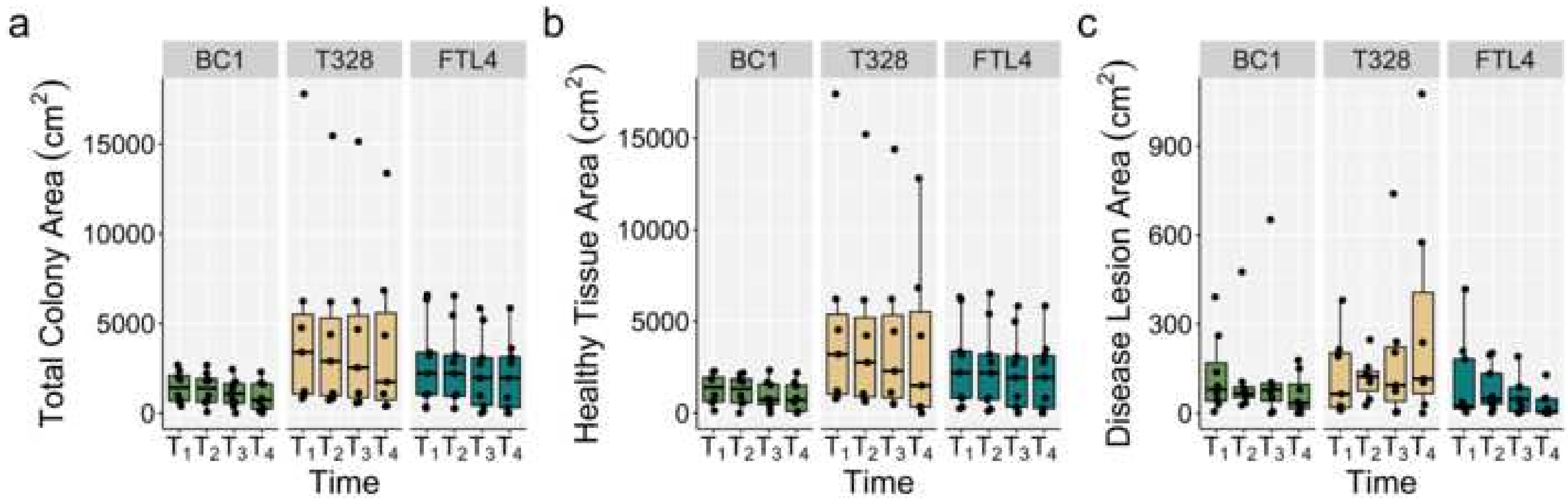

S1 Table

Click here to access/download
Supporting Information
S1 Table.docx

S2 Table

Click here to access/download
Supporting Information
S2Table.docx

S3 Table

Click here to access/download
Supporting Information
S3Table.docx

S4 Table

Click here to access/download
Supporting Information
S4 Table.docx

S5 Table

Click here to access/download
Supporting Information
S5 Table.docx

S1 Fig

Click here to access/download
Supporting Information
S1 Fig.tif

S2 Fig

Click here to access/download
Supporting Information
S2 Fig.png

S3 Fig

Click here to access/download
Supporting Information
S3 Fig.png

S4 Fig

Click here to access/download

Supporting Information

S4 Fig.pdf

S1 Dataset

Click here to access/download
Supporting Information
S1 Dataset.xlsx

Click here to access/download
Supporting Information
S2 Dataset.csv

S3 Dataset

Click here to access/download
Supporting Information
S3 Dataset.csv
