# Supplementary material for: Quantifying impacts of stony coral tissue loss disease on corals in Southeast Florida through surveys and 3D photogrammetry
Source: PLoS One. 2021 Jun 25;16(6):e0252593. doi: 10.1371/journal.pone.0252593 (PMC8232449; doi:10.1371/journal.pone.0252593)
Supplement: Attachment — Submitted filename: PONE-D-20-25422_R1_V2.pdf [file pone.0252593.s009.pdf]

PLOS ONE

Quantifying impacts of stony coral tissue loss disease on corals in Southeast Florida through surveys and 3D photogrammetry

--Manuscript Draft--

**Manuscript Number:**	PONE-D-20-25422R1
**Article Type:**	Research Article
**Full Title:**	Quantifying impacts of stony coral tissue loss disease on corals in Southeast Florida through surveys and 3D photogrammetry
**Short Title:**	Impacts of stony coral tissue loss disease on corals through surveys and 3D photogrammetry
**Corresponding Author:**	Ian Robert Combs, M.Sc<br>Harbor Branch Oceanographic Institute<br>Fort Pierce, FL UNITED STATES
**Keywords:**	stony coral tissue loss disease; 3D photogrammetry; photogrammetry; coral health and disease; coral reef ecology; coral; Florida Reef Tract; coral reef ecosystems; Southeast Florida; 3D modeling; SCTLD; Caribbean coral reefs; Tropical Western Atlantic
**Abstract:**	Since 2014, stony coral tissue loss disease (SCTLD) has contributed to substantial declines of reef-building corals in Florida. The emergence of this disease, with its ability to impact more than 20 scleractinian coral species, has generated a need for widespread reef monitoring and the implementation of novel survey and disease mitigation strategies. This study paired SCTLD prevalence assessments with colony-level monitoring to help improve understanding of disease dynamics on both individual coral colonies and reef-wide scales. Benthic surveys were conducted throughout the northern Florida Reef Tract to monitor the presence/absence of disease, disease prevalence, and coral species affected by SCTLD. Observed SCTLD prevalence was lower in Jupiter and Palm Beach than in Lauderdale-by-the-Sea or St. Lucie Reef, but there were no significant changes in prevalence over time. To assess colony-level impacts of the disease, we optimized a low-cost, rapid 3D photogrammetry technique to fate-track infected *Montastraea cavernosa* coral colonies over four time points spanning over three months. Total colony area and healthy tissue area on fate-tracked colonies decreased significantly over time, but disease lesion area did not, and was not correlated with total colony area. These results suggest that SCTLD affects colonies regardless of size, therefore larger colonies, when present, should be prioritized for colony-level intervention efforts to maximize impact. Traditional coral surveys combined with 3D photogrammetry can provide greater insights into the spatiotemporal dynamics and impacts of coral diseases on individual colonies and coral communities than surveys or visual estimates of disease progression alone.
**Order of Authors:**	Ian Robert Combs, M.Sc
	Michael S Studivan
	Ryan J Eckert
	Joshua D Voss
**Response to Reviewers:**	Editor:<br>Reviewer 1 in particular notes that there is a significant literature on photogrammetry techniques on coral reefs, and further attention to this would improve both the introduction and discussion, rather than relying on citations to other taxa. They also require increased clarity in the methods regarding validation of the accuracy of the model, including what site-specific community parameters would impact the 3D model generation. Reviewer 2 also brings up a salient point here, which is that there was no direct comparison to 2D methods, so claims of increased accuracy are not demonstrated. They do recommend that a relatively rapid post-hoc statistical comparison with 2D screen grabs could be considered, or more cautious language. I would suggest the former to increase the rigor of your study, but either would be sufficient. Reviewer 1 also questions the clarity of statistical analysis, and believes that further investigation into why shape area and error varies with location is either

Powered by Editorial Manager® and ProduXion Manager® from Aries Systems Corporation

required, or explained in the text.
¬-We took only non-scaled reference photographs and thus cannot conduct a 2D comparison with our 3D data, as such we chose to use more cautious language throughout the manuscript.
Both reviewer 1 and I are curious as to why the grouping of disease prevalence is at the county level rather than the location/site scale, particularly since you do not discuss different management, water quality or land use differences between counties (See Fig 3). By working at the location scale, this would allow comparison of more similar replicates, and would also be more accessible to a person not familiar with the geography of Florida. If you feel that county is an important part of the puzzle that makes it a more appropriate unit of replicate than location (more than just indicating a north - south gradient for example), it would be useful to explain this decision.
-We agree with the editor and reviewers comments and reanalyzed the survey data to group at the Location level.
Both reviewers identify the recommendation of 'culling' as unjustified, or presented with too high a level of certainty, particularly since the effort, efficacy and description of what other interventions are available are not given (if there is only one colony in an area infected, but it is large, why not remove that if it leads to a better outcome than other interventions?). Without evidence presented here, or citations to small colonies dying more quickly or being disease vectors, this management recommendation goes far beyond the work. Indeed, in Fig S2 It could be argued that there is some evidence that some larger colonies might lose tissue at a faster rate than some small colonies. In the discussion you also state that "disease lesion area did not correlate with total colony size suggesting that larger colonies do not exhibit a higher proportion of diseased tissue" - this could imply they show the same proportional loss or lower. I strongly suggest you present more data on proportional loss of colony tissue (not just absolute loss) if this is an argument you wish to make. Even so, suggesting culling small colonies as a management measure would still be extreme and would require a stronger argument with references to small colonies as vectors or disease reservoirs. The argument also begs the question - what is a small colony? Nor are what interventions that might be possible shy of culling the entire colony described.
-We removed the suggestion of culling from the manuscript.

Finally, there is a significant amount of relevant context and data in the SI. It would be good to see some of the tables and figures into the main text, particularly concerning the disease incidence, colony size and disease spread data.
-We revised our results section to remove unnecessary metrics and focused on the more compelling outcomes from this study. We also included additional figures focusing on correlation between colony size and disease area as well as rate of tissue loss.
Reviewer 1:
Reviewer #1: Reviewer comments:

1) from L88: I think applications on cetacean and elasmobranch research are way out of the scope of this work. Better concentrate on referencing all the exciting research on coral reefs.
- We have removed the unnecessary references to cetacean and elasmobranch research and greatly increased our literature review concerning photogrammetry on corals and coral reefs. We appreciate the suggested journal articles.

2) Also, in this section, I don’t think you need to cite aerial and scanner methods to prove your point, and the two studies you cite for coral growth did not use photogrammetry at all. Use Ferreira et al. 2017 and Lange & Perry 2020 if you want to keep this sentence. Alternatively you could explain photogrammetry and SfM first and then give examples of studies on reefs. Also see annotations in the PDF.
- Reference to aerial and scanner methods have been excluded and the focus was shifted to in-water photogrammetry methods, we also corrected the two citations for Ferreira et al. 2017 and Lange & Perry 2020.

3) L. 104: optimized from what basis? You have not developed the photogrammetry technique, but a new application for it. Maybe say: “In this study we used photogrammetry techniques as described in [Young et al.] in order to develop a new application, i.e to track SCTLD disease progression in M. cavernosa colonies.” or

something similar?
-We revised the wording of this to better represent our work (L109).

Methods:

1) see annotations in PDF to improve clarity of the text.
-The PDF annotations were applied, they were very helpful and the authors thank the reviewer for their efforts to improve the clarity and writing throughout this manuscript.

2) L181: state version of Agisoft Metashape that you used as some versions seem to have issues and it will help for repeatability.
-This has been included (L181).

3) L. 215: I do not understand why site-specific “community parameters” (what is this anyways?) or even water quality should affect 3D model generation. This would mean that the method is not accurate and should not be used.
- Our intention was to compare surface area error from models constructed in varying environments (pristine pool versus nearshore coral reef with low visibility and high wave action). To make the model accuracy validation section more clear and applicable to the fate-tracking results, we have focused on comparisons of shape error between the pool models and models made at the fate-tracking sites in Lauderdale-by-the-Sea. We have rephrased this section to be more explicit starting at L214.

Related to this it does not make sense to compare the area of shapes among locations to evaluate the 3D models. So I am wondering why you did not just photograph the mock colony at the sites where you did the fate-tracking in order to calculate the accuracy of your methodology.
- Models with the mock coral colony were constructed at the same fate-tracking sites in the original version of the manuscript, but were not represented as separate sites in the original analysis. To improve the comparability of the model accuracy analyses to the fate-tracking portion, we have focused on the fate-tracking sites in Lauderdale-by-the-Sea only. Likewise, we have removed the analyses of overall shape areas and focused on the shape error associated with each model.
Concerning this there is two parameters you want to check:

A) accuracy, meaning how close to reality is your 3D model. Test this by comparing measured shape areas on your mock corals to known areas and calculate the mean error. (This is potentially how you got your 2.17 cm2?) If you notice that the error is bigger at a more turbid reef site, then you could conclude that the visibility affects the accuracy of model building/measurement. But direct comparison of shape areas among sites does not make sense.

B) precision, meaning how good is the reproducibility of your measurements. For this you should measure the surface of the same shape, or better the same coral colony, several times, including all the hand tracing around colonies etc. Then calculate the error, which will show you if you introduce considerable variability using your workflow.

You should calculate the coefficient of variation (error/average) in order to compare your errors to other studies doing surface area measurements. You cannot compare the error from small shapes (2 cm2) directly to the surface area of the colony.
- We have made substantial efforts in the data analysis and writing to make this section of the manuscript clearer and more applicable to the coral fate-tracking portion. First, we focused on comparison of model error across the pool and the three coral fate-tracking sites in Lauderdale-by-the-Sea. The statistical analyses were revised to a comparison of shape error (i.e. the difference between 3D modeled shape areas and the template) among sites (L214-222, L225-230, L308-3111). Replicate measurements of all the prism shapes were made within the original analyses, but this was not clear in the original version. The prism had five faces with the template shapes (top and four sides), each of which had multiple shapes (four squares and rectangles, one circle per face) (L222-225). Shape error is also represented in two ways, as 1) the absolute difference between measured shape areas and template area, and 2) percent error relative to template area (similar to previous studies, including those you suggested in the original review). The former is used for the statistical tests and for visual

assessment of shape error in S4 Fig, and the latter is reported in the text (L311-314). These calculations result in mean errors of 1.70 sq. cm and 6.13%, respectively. The coefficient of variation was not calculated for these analyses, as this is typically used for comparisons of replicate 3D models or measurements. Each of the measurements in our prism dataset are statistically independent, and we feel provide for a robust assessment of model accuracy.

L 240: I do not understand why these correlations were done and what they would tell us about the accuracy of the method.

- This analysis has been removed, and the section has been rewritten according to the revised analysis of accuracy across sites.

Results:

1) I am wondering why did you choose to compare counties instead of locations as shown on the map? The latter would have the advantage that replications are more similar.
-The disease prevalence survey analyses were shifted from county level to location level as shown on the map.

2) L 255-260: I don't think the figure supports these statements. I would say something along the line of “in Broward, disease prevalence stayed quite constant at around 10%, while in Palm Beach prevalence was usually very low, but peaked in March 2019 when ... of colonies were affected. Disease prevalence at Martin was very variable due to low numbers of live coral colonies...”
-This section has been edited to improve clarity and better reflect our results and figures (L238-255).

3) L 273: Has it been taken into account that colonies were measured repeatedly? Considering that the areas of your colonies are very different you might either have do more fancy statistics using repeated measures GLM/GAM (maybe using colony ID as fixed factor and site and time as explaining variables? sorry, not an expert myself) or you might have to calculate “loss of area” or “loss % of area” in order to make meaningful comparisons over time.

I know you calculated rate of change in healthy and diseased tissue, which I think might make more sense than comparing actual area. In general it is getting a bit confusing looking at so many parameters. I would suggest to think carefully which parameters are most useful in showing what you are interested in and rather use fewer but explain better what they mean.

E.g., I think the most interesting results and the best order in the section L277-309 would be (add numbers and statistics): “The rate of tissue loss did not differ among sites but was variable over time, with highest loss in the first observation interval. The diseased area however did stay constant over time, indicating that the lesion moves with the infected tissue.” Then add the correlations you think are useful. Not all are I think.
-The Friedman’s test and subsequent post hoc analysis, the Nemenyi test, are appropriate since our data is not normally distributed; it is a non-parametric equivalent to a repeated measures analysis of variance. The section has been reduced in the number of metrics and tests run to increase overall clarity and focus on important, compelling results. (L289-297). Proportion of loss data was added, and the only correlation that was retained, as the authors thought this was the most compelling, was the correlation between disease lesion area and total colony size.

4) L287 and 288: S2 Fig should be S3 Fig?
- These were removed.

5) L 312-L334: This whole section does not make sense to me. I do not understand why the areas of shapes should be different depending on location. This definitely does not increase trust in the method! It might in part be a relic of your different sample sizes and tests you do. Why do you run ANOVA then Kruskal Wallis and then 3-way PERMANOVA? I fail to understand what any of these significances mean and it is not

explained in the results or discussion sections.
- In the revised version of the manuscript, we have streamlined our assessments of model accuracy according to previous suggestions. We hope that the new sections present a better description of the analyses that were conducted. Also, following careful review of the statistical tests and 3D models, we identified a few outliers in the prism dataset that were the result of holes in the mesh leading to inaccurate surface area measurements, which appeared to be driving the significant differences presented in the original manuscript.

I would suggest to think carefully about what parameters actually tell you something about the accuracy/precision of the analysis (see comments above) and rewrite this section completely after the improved analysis.

- Thank you for your constructive comments, we hope that the revised sections address your concerns and suggestions.

Discussion:

This section could be improved by being very clear how the results compare to other studies and what can be said about the implications. See annotations in the PDF

1) L353: It is not clear what you are trying to say here. Also please make clear what you mean with L356-357. Next section can be shortened as suggested in PDF.
- This section, along with the section below, were combined and rewritten to improve clarity (L324-350).

2) L363: This paragraph seems to be repeating discussions from the previous paragraph. Maybe you could combine those better without repeating yourself?

I think the order of these two paragraphs could be improved. E.g. “In the FRT, disease prevalence is typically higher (...%) than elsewhere in the TWA (~1-3%) [57]. Sites in Palm Beach in the present study showed relatively low background disease (6%), similar to prevalence across the NFRT after Hurricane Irma in Sept 2017 (6%???) (Walker 2018, 53). Highest values observed in this study were 20-45% at sites in Martin, but did not reach levels of up to 60% as reported in [17], likely due to low abundance of susceptible species after ongoing SCTLD impacts [53,58]”
-The following section was changed using your suggestions to increase clarity (L332-338).

3) L384: I thought Fig S2 showed that it does NOT correlate? Otherwise your next sentence does not make sense
- This line was edited to correct the typo, the sentence should have read “rates of tissue loss did not correlate” as stated in the reviewer comment.

4) I think the suggestion to cull small colonies is a bit drastic and not supported by your study. If there is other research supporting this approach please cite here.
- The suggestion of culling has been removed from the manuscript

5) L384-393 could be condensed down to 1-2 sentences saying management should target larger colonies.
-This section was revised to omit culling and express the need to target larger colonies.

6) From L 403: This whole sections would have to be revised after analysis of accuracy/precision.
- Following the revised analysis, we have rewritten this section to emphasize the error rate in the prism measurements (L379-382), the potential causes (flexible shape templates and water movement/poor visibility; L382), and suggestions for improving model construction and underwater filming/photography (L384-391).

7) L406: It is not possible to compare the 2.17 cm2 shape error directly to the much larger colonies. Actually, this error seems very high considering that the shape on your mock coral is not very big?! Does that relate to about 10%? Calculate CV and compare to other studies. Also, if you have an error of about 10% you might want to check if the

difference of total area/healthy area between time points would still be significant.
- Based on previous studies, we have opted to calculate error rate (%) for shape measurements relative to the corresponding template area. As a result, the global error rate is ~6%.

8) L409: you did not test the effect of colony size on model accuracy. Also, it does not make sense that water quality (do you mean turbidity/visibility?) affects the size of colonies. You are probably trying to say that lower visibility could result in lower model quality affecting measurements of surface area? Be precise in your wording.

- We have revised this section to be more specific regarding surface area measurements and potential effects of low visibility on model construction and error (L389).

Conclusions and general:

1) Different kinds of interventions should be mentioned in the introduction or discussion if it is the main discussion point in the conclusion.
-There has been a wide variety of intervention methods proposed, and refinement and evaluation is still ongoing, so we did not go into detail on the specific methods, but did include them in the introduction (L77-82).

2) L435: Why would interventions be unsuccessful in small colonies? They should work the same, just preserve less total area, right? So I understand prioritizing big ones, but in sites were prevalence is low why not treat small ones too.
-The reference to interventions being unsuccessful was removed and clarification was added to imply that larger colonies should be prioritized if present, but smaller colonies should not be ignored all together.

3) I think the discussion and conclusion sections will benefit from a second round of review after the improved model evaluation. Try to be very clear what the novelty of this work is (new application of photogrammetry method to accurately quantify tissue loss/disease progression over time) and what can be concluded from it (improve survey protocols, evaluate success of intervention methods... I don't think culling of half the colonies is a good outcome here).
-The discussion and conclusions have been revised to focus more on the novelty of this work, comparable photogrammetry and SCTLD studies, and improving the management and intervention suggestions throughout.

4) If you want people to take up this method, make the workflow easier to access. I know it is all in the GitHub repository, which is awesome, but I did not easily find the step-by-step guide and what you actually did to measure the areas. Maybe you could prepare a one-pager which is easy to print, stating the steps of image acquisition, model building and analysis to go into Suppl Methods. Or put a link in the methods which leads straight to the guide instead of the whole repository.
- A reference to the protocol within our GitHub repository was added to the methods section. (L178)

5) Also, it would be great if you could make the models (as .obj?) available in the repository.
-Unfortunately, the models are too large to be housed on the GitHub repository, they will be available upon request.

Reviewer 2:
General comments
- One of the major findings of this study is that the 3D model method presented is "more accurate data than previously established methods such as two-dimensional surface area estimation (ln 398-399)." I thought the assessment of the accuracy of the 3D method presented here was excellent and rigorous, but there was not a estimate of the disease lesions or the calibration templates/mock coral made from the same two-dimensional photographs to make a robust and unbiased statistical comparison of the two techniques. While I agree that this 3D model pipeline is achievable, it does still require ~40 min per colony of rendering, plus fairly expensive software and hardware

for modelling and storage of large video files compared with the much simpler 2D method, so a quantitative comparison of the two methods would strengthen the argument and add justification for using the 3D model method presented here.
-We took 2D reference photographs in order to double check possible abnormalities in the model (eg: if that is a Christmas tree worm or a small disease lesion) they are not scaled and therefore cannot be analyzed and used in a comparison of the two techniques. However, we have incorporated references to previous studies that have compared 3D photogrammetry to 2D and physical methods (tin foil method) that have shown 3D photogrammetry to be a more robust method.

- The inclusion of more data from the disease surveys in the main text could be useful, and would balance the disease ecology with the methodological aspects of the study a bit better. Including which species were (and were not) affected at each site, and how disease prevalence changed over time by taxa within each site would be a nice addition to the main text. Line 365-7 mentions the "low abundance and species richness in Marin County sites compared with PB and Broward Counties", but what is the density and species richness within each site?

-We feel that adding broader ecological surveys of these reefs outside of SCTLD prevalence is outside the scope of this study. We included more species abundance data, mainly to highlight the lack of 'highly susceptible' species within our surveys is possibly due to SCTLD already and how different the coral communities are at SLR relative to the rest of our sites.

- The manuscript is relatively sparse wrt figures and tables, with the vast majority of the information presented as supplementary. I would suggest including a few of these in the main text, particularly Fig S2 and possibly S4, again to balance out the ecology/methodology aspects of the manuscript.
We included additional figures focusing on correlation between colony size and disease area as well as rate of tissue loss.
Several Specific comments
- Line 193 – it is quite tricky to determine whether the stark white area of the lesion is still alive or newly dead, especially in an image/video. Would it be more appropriate to define the lesion area as the 'stark white coral tissue or very newly dead white skeletal area'? If not, how was this distinction made? It is very difficult to tell in Figure 1, but the white lesion areas look like there is no live tissue to me.
- We revised this to improve clarity (L193)

- Line 282 – consider rounding the estimates of # of lesions per colony to the nearst whole number as that makes the most conceptual sense (or at least to the 10th which is probably a more appropriate significant digit)
-These were removed from the analysis .

- Line 360 – consider stating what the most susceptible species are.
-This has been included (L340)

- Line 384 – was the correlation between rates of tissue loss and total colony size positive or negative? Please specify.
-This was a typo, it should have read "did not" correlate, it has been adjusted (L361)

- Line 388 – typo – suggest 'greater preservation'
- This section has been rewritten

- Line 389 – is there justification for the statement that smaller colonies are more likely to succumb to SCTLD? It may take less time to succumb due to the rate of tissue loss, but is the likelihood of dying from the disease actually higher? Has much recovery been observed? I would suggest editing to "smaller infected colonies may succumb more quickly to SCTLD"
-This section was heavily revised as suggested and the statement regarding small colonies has been omitted.

- Line 406 – perhaps you can expand on what you mean by 'sufficient replication'? Does this mean replicate videos of each colony to render multiple models per colony?
-This section has been heavily revised and this line has been removed.

Powered by Editorial Manager® and ProduXion Manager® from Aries Systems Corporation

	- Line 413 – suggest 'most commonly'<br>-This section has been heavily revised and this line has been removed.<br><br>- Line 434 – suggest 'regarding' rather than 'relative to'<br>-This has been changed as suggested (L406)<br><br>- I wonder if it is worth commenting on how this model approach may work for other coral growth morphologies. Mcavernosa is a fairly simple mounding shape, but encrusting, foliose or branching corals may be much more difficult to accurately model?<br><br>- I have strong reservations about the mitigation technique of culling small colonies that is suggested in line 438 (and 389, see comment above). I understand that they may die more quickly than larger colonies and could act as sources of the disease. But the data presented here do not indicate that they are more susceptible (are there size-frequency distributions of healthy vs/ diseased corals in a population?), and the small colonies are often offspring of the few remaining colonies that have survived multiple selection events already and may be the best hope of the recovery of resilient communities, especially if there is any evidence that colonies can recover from this disease. Thus I much prefer the second option of focusing intervention resources on larger colonies and would urge caution when considering culling of smaller colonies.<br>-The suggestion of culling has been removed throughout the manuscript.<br><br>- Fig 1. Typo in fig legend – add 'a' before 'colony'<br>-This has been changed as suggested.
**Additional Information:**	
**Question**	**Response**
**Financial Disclosure**<br><br>Enter a financial disclosure statement that describes the sources of funding for the work included in this submission. Review the submission guidelines for detailed requirements. View published research articles from *PLOS ONE* for specific examples.<br><br>This statement is required for submission and **will appear in the published article** if the submission is accepted. Please make sure it is accurate.	Funding for this research was awarded to J. Voss from the Florida Department of Environmental Protection, Environmental Protection Agency (South Florida Geographic Initiative award X7 00D667-17 with J. Beal as co-investigator, https://floridadep.gov/), and the NOAA Coral Reef Conservation Program (award NA16NOS4820052, https://coralreef.noaa.gov/). Additional funding was awarded to I. Combs by the Harbor Branch Oceanographic Institute Foundation through the Indian River Lagoon Graduate Research Fellowship (https://hboifoundation.org/).

**Unfunded studies**<br>Enter: *The author(s) received no specific funding for this work.*<br><br>**Funded studies**<br>Enter a statement with the following details:<br>• Initials of the authors who received each award<br>• Grant numbers awarded to each author<br>• The full name of each funder<br>• URL of each funder website<br>• Did the sponsors or funders play any role in the study design, data collection and analysis, decision to publish, or preparation of the manuscript?<br>• **NO** - Include this sentence at the end of your statement: *The funders had no role in study design, data collection and analysis, decision to publish, or preparation of the manuscript.*<br>• **YES** - Specify the role(s) played.<br><br>* typeset	
**Competing Interests**<br><br>Use the instructions below to enter a competing interest statement for this submission. On behalf of all authors, disclose any competing interests that could be perceived to bias this work—acknowledging all financial support and any other relevant financial or non-financial competing interests.<br><br>This statement **will appear in the published article** if the submission is accepted. Please make sure it is accurate. View published research articles from *PLOS ONE* for specific examples.	The authors have declared that no competing interests exist.

**NO authors have competing interests**<br><br>Enter: *The authors have declared that no competing interests exist.*<br><br>**Authors with competing interests**<br><br>Enter competing interest details beginning with this statement:<br><br>*I have read the journal's policy and the authors of this manuscript have the following competing interests: [insert competing interests here]*<br><br>* typeset	
**Ethics Statement**<br><br>Enter an ethics statement for this submission. This statement is required if the study involved:<br><br>• Human participants<br>• Human specimens or tissue<br>• Vertebrate animals or cephalopods<br>• Vertebrate embryos or tissues<br>• Field research<br><br>Write "N/A" if the submission does not require an ethics statement.<br><br>General guidance is provided below. Consult the submission guidelines for detailed instructions. **Make sure that all information entered here is included in the Methods section of the manuscript.**	For work conducted at St. Lucie Reef (SLR): St. Lucie Inlet State Park Permit 06261715, issued by St. Lucie Inlet State Park and FWC SAL-17-1960-SCRP, issued by Florida Fish and Wildlife Conservation Commission<br><br>For work conducted in Jupiter (JUP), Palm Beach (PB) and Lauderdale-by-the-Sea (LBTS): FWC SAL-17-1960-SCRP, issued by Florida Fish and Wildlife Conservation Commission

Powered by Editorial Manager® and ProduXion Manager® from Aries Systems Corporation

**Format for specific study types**<br><br>**Human Subject Research (involving human participants and/or tissue)**<br>• Give the name of the institutional review board or ethics committee that approved the study<br>• Include the approval number and/or a statement indicating approval of this research<br>• Indicate the form of consent obtained (written/oral) or the reason that consent was not obtained (e.g. the data were analyzed anonymously)<br><br>**Animal Research (involving vertebrate animals, embryos or tissues)**<br>• Provide the name of the Institutional Animal Care and Use Committee (IACUC) or other relevant ethics board that reviewed the study protocol, and indicate whether they approved this research or granted a formal waiver of ethical approval<br>• Include an approval number if one was obtained<br>• If the study involved *non-human primates*, add *additional details* about animal welfare and steps taken to ameliorate suffering<br>• If anesthesia, euthanasia, or any kind of animal sacrifice is part of the study, include briefly which substances and/or methods were applied<br><br>**Field Research**<br><br>Include the following details if this study involves the collection of plant, animal, or other materials from a natural setting:<br>• Field permit number<br>• Name of the institution or relevant body that granted permission	
**Data Availability**<br><br>Authors are required to make all data underlying the findings described fully available, without restriction, and from the time of publication. PLOS allows rare exceptions to address legal and ethical concerns. See the PLOS Data Policy and FAQ for detailed information.	Yes - all data are fully available without restriction

A Data Availability Statement describing where the data can be found is required at submission. Your answers to this question constitute the Data Availability Statement and **will be published in the article**, if accepted.<br><br>**Important:** Stating 'data available on request from the author' is not sufficient. If your data are only available upon request, select 'No' for the first question and explain your exceptional situation in the text box.<br><br>Do the authors confirm that all data underlying the findings described in their manuscript are fully available without restriction?	
**Describe where the data may be found in full sentences. If you are copying our sample text, replace any instances of XXX with the appropriate details.**<br><br>• If the data are **held or will be held in a public repository**, include URLs, accession numbers or DOIs. If this information will only be available after acceptance, indicate this by ticking the box below. For example: *All XXX files are available from the XXX database (accession number(s) XXX, XXX.*).<br>• If the data are all contained **within the manuscript and/or Supporting Information files**, enter the following: *All relevant data are within the manuscript and its Supporting Information files.*<br>• If neither of these applies but you are able to provide **details of access elsewhere**, with or without limitations, please do so. For example:<br><br>*Data cannot be shared publicly because of [XXX]. Data are available from the XXX Institutional Data Access / Ethics Committee (contact via XXX) for researchers who meet the criteria for access to confidential data.*<br><br>*The data underlying the results presented in the study are available from (include the name of the third party*	All relevant data are within the manuscript and its Supporting Information files.<br><br>All data files, protocols, analyses pipelines and scripts can be found in our GitHub repository (https://github.com/icombs2017/analysisOf3dModels)

Powered by Editorial Manager® and ProduXion Manager® from Aries Systems Corporation

and contact information or URL).<br>• This text is appropriate if the data are owned by a third party and authors do not have permission to share the data.<br><br>* typeset	
Additional data availability information:	Tick here if the URLs/accession numbers/DOIs will be available only after acceptance of the manuscript for publication so that we can ensure their inclusion before publication.

Clean Version of Manuscript
Click here to access/download;Manuscript;Manuscript_Clean.docx

Quantifying impacts of stony coral tissue loss disease on corals in Southeast Florida through surveys and 3D photogrammetry

Ian R Combs[1,2*], Michael S Studivan[1,3], Ryan J Eckert[1], Joshua D Voss[1]

[1] Department of Biological Sciences, Harbor Branch Oceanographic Institute, Florida Atlantic University, Fort Pierce, Florida, United States of America

[2] Current address: Elizabeth Moore International Center for Coral Reef Research & Restoration, Mote Marine Laboratory, Summerland Key, Florida, United States of America

[3] Current address: Cooperative Institute for Marine and Atmospheric Studies, University of Miami, Miami, Florida, United States of America

* combsi9892@gmail.com

Abstract

Since 2014, stony coral tissue loss disease (SCTLD) has contributed to substantial declines of reef-building corals in Florida. The emergence of this disease, with its ability to impact more than 20 scleractinian coral species, has generated a need for widespread reef monitoring and the implementation of novel survey and disease mitigation strategies. This study paired SCTLD prevalence assessments with colony-level monitoring to help improve understanding of disease dynamics on both individual coral colonies and reef-wide scales. Benthic surveys were conducted throughout the northern Florida Reef Tract to monitor the presence/absence of disease, disease prevalence, and coral species affected by SCTLD. Observed SCTLD prevalence was lower in Jupiter and Palm Beach than in Lauderdale-by-the-Sea or St. Lucie Reef, but there were no significant changes in prevalence over time. To assess colony-level impacts of the disease, we optimized a low-cost, rapid 3D photogrammetry technique to fate-track infected *Montastraea cavernosa* coral colonies over four time points spanning over three months. Total colony area and healthy tissue area on fate-tracked colonies decreased significantly over time, but disease lesion area did not, and was not correlated with total colony area. These results suggest that SCTLD affects colonies regardless of size, therefore larger colonies, when present, should be prioritized for colony-level intervention efforts to maximize impact. Traditional coral surveys combined with 3D photogrammetry can provide greater insights into the spatiotemporal dynamics and impacts of coral diseases on individual colonies and coral communities than surveys or visual estimates of disease progression alone.

Introduction

Coral cover in the Tropical Western Atlantic (TWA) has declined over the last four decades [1,2], and coral diseases have been identified as one major driver of widespread coral decline throughout the region [3]. In the 1990s, white band disease dramatically reduced coral cover of *Acropora cervicornis* and *Acropora palmata* by 95% in the TWA [4]. At the same time, in the Florida Keys, white pox was responsible for up to a 70% reduction in *A. palmata* cover [5]. Additionally, increased disease prevalence following a coral bleaching event in 2005 caused a 60% decline in coral cover throughout the U.S. Virgin Islands [6].

Coral diseases, regardless of their host specificity, have contributed to the decline of many integral reef-building scleractinian species. While white pox, white band, and acute *Montipora* white syndrome are genus-specific, other diseases have a broad, even pan-oceanic host range [6–8]. For example, white plague type II affects 17 species of scleractinian coral across multiple genera [9]. Widespread decreases in coral cover from diseases result in an ecological shift from diverse, coral-dominated communities to more homogenous, ~~algae-~~dominated communities [10,11]. Such shifts have the potential to create long-term changes in fish assemblages and fishery yields [12] as well as the loss of key ecosystem services such as fisheries habitat [13], coastal wave protection [14] and nutrient cycling [15] that can persist long after a coral disease event subsides.

Since 2014, the Florida Reef Tract (FRT) has experienced an ongoing outbreak of a newly-described coral disease responsible for widespread coral mortality throughout the TWA. Stony coral tissue loss disease (SCTLD) is characterized as a highly virulent disease that affects over 20 species of scleractinian corals in the TWA [16]. SCTLD first appeared in the summer of 2014 following the dredging of Government Cut in Miami-Dade County [17,18]. In subsequent

years, reports of SCTLD infections have increased and spread from Miami-Dade County along the Florida Reef Tract (FRT) and into the wider TWA. To date, SCTLD has spread north to the northern terminus of the FRT in Martin County and south past the Marquesas Keys in Monroe County, with additional outbreaks observed in at least twelve territories throughout the TWA [19,20].

SCTLD manifests as lesions of necrotic tissue that spread across a colony, leaving behind denuded coral skeleton (Fig 1) [16]. SCTLD is histologically distinct from white plague, exhibiting a fast-acting, liquefactive necrosis after lesions develop deep within coral tissue and progress to the colony surface [21]. Across multiple host coral species, SCTLD-affected colonies demonstrate altered microbial communities relative to their apparently healthy counterparts [22–24]. While both microbial culture-based and sequencing studies are ongoing, no pathogen for SCTLD has been identified to date. Various intervention methods have been proposed and trialed including probiotic treatments, physical interventions, and various topical treatments [25]. The most popular has been a combination of physical intervention by trenching a firebreak around the infected tissue and covering the infected tissue with a topical antibiotic application [26]. Regardless of the method, the various proposed techniques require individual evaluation and continued monitoring to determine success.

Fig 1. SCTLD lesions on a colony of *Montastraea cavernosa*

(a) Fate-tracked, SCTLD-infected *Montastraea cavernosa* with (b) rendered 3D model, (c) characteristic disease lesion, and (d) necrotic tissue.

Traditionally, coral disease monitoring has relied on a combination of benthic survey methods and individual colony monitoring, the latter often based on estimations of disease area, *in situ* linear measurements, or planar photography [27,28]. However, these techniques may incorporate a substantial observer bias or uncertainties associated with 2D estimations of 3D surfaces [29]. Under water photogrammetry and 3D modeling of coral colonies is an emerging technique that offers the potential to enhance both the accuracy and speed of data collection. Structure-from-motion (SfM) photogrammetry derives 3D structure from a series of overlapping images, similar to established stereoscopic methods. Rather than requiring known *a priori* positions, SfM orientation is resolved based on common features extracted from overlapping images [30]. Conveniently, this can be done using a moving sensor, or from still images generated by an individual with a camera. Photogrammetry has been used in a number of coral reef applications including habitat characterizations [31–33], surface and volume measurements [34,35], and growth measurements [36,37]. SfM photogrammetry was first implemented on coral colonies by Bythell et al. [38] which used 10–20 overlapping photographs to generate 3D models. As technology has progressed and computational power has become increasingly affordable, SfM photogrammetry techniques for the purpose of assessing various coral metrics have been refined and improved upon [34,37]. Changes in image capture and model reconstruction have improved measurement accuracy and precision, especially in regards to more established surface area methods such as the foil-wrapping method [29,34,37,39,40]. As such, SfM photogrammetry has emerged as a powerful and commonly-used tool for coral reef researchers.

In this study, we adapted a photogrammetry technique described in Young et al. [40] to track disease progression in fate-tracked *Montastraea cavernosa* colonies in Southeast Florida to

gain insight into the colony-level dynamics of SCTLD. The study was designed to provide insight into colony- and community-level dynamics of this poorly understood disease by combining traditional surveys with colony fate-tracking using SfM photogrammetry. The ultimate goal is to increase widespread application of this and similar techniques to improve the design, implementation, and success of coral disease intervention, mitigation, and management strategies.

Methods

Disease prevalence surveys

Four locations across the northern Florida Reef Tract (NFRT) were selected for disease prevalence surveys: St. Lucie Reef (SLR), Jupiter (JUP), Palm Beach (PB), and Lauderdale-by-the-Sea (LBTS, Fig 2). The work conducted at SLR was done so under the St. Lucie Inlet State Park Permit 06261715, issued by St. Lucie Inlet State Park and FWC SAL-17-1960-SCRP, issued by Florida Fish and Wildlife Conservation Commission. The work conducted at JUP, PB, and LBTS was conducted under FWC SAL-17-1960-SCRP, issued by Florida Fish and Wildlife Conservation Commission. Following Hurricane Irma in September 2017, a rapid-response damage and disease survey effort was conducted throughout Southeast Florida [41]. The data from these initial surveys were used to select the sites for the present study based on occurrence of coral communities and prevalence of SCTLD (Fig 2).

Fig 2. Map of study locations throughout the Northern Florida Reef Tract Florida Red circles indicate roving diver survey sites and red triangles indicate sites where both roving diver surveys and coral fate-tracking occurred (Florida boundary and benthic hard bottom shapefile source: Florida Fish and Wildlife Conservation Commission-Fish and Wildlife Research Institute).

Roving diver disease surveys were conducted approximately monthly from November 2017 to June 2019. These surveys were designed to assess the greatest reef area possible, quantifying disease prevalence over an estimated range of 100–2000 m^2 per survey dependent on underwater visibility. SCUBA divers swam for 20 min and recorded the species and disease status of every living coral colony ≥10 cm in diameter. Paling, partial bleaching, and bleaching were also noted. SCTLD abundance and prevalence were calculated from these data. Statistical tests were run in the R statistical environment [42]. Non-parametric tests were implemented for all analyses as datasets were non-normal and normal distributions could not be achieved through transformation. Permutational analysis of variance (PERMANOVA; 9999 permutations) was used in the package *vegan* [43] to assess variation in disease prevalence among sites and survey times, with Bonferroni corrected pairwise comparisons using the package *pairwiseAdonis* [44].

Fate-tracking of SCTLD-affected *M. cavernosa*

Benthic survey data indicated that disease incidence was too low at sites in Jupiter and Palm Beach, and that coral abundance was too low at sites in St. Lucie Reef, to conduct statistically robust fate-tracking studies in these locations. Consequently, only three fate-tracking sites were established in Lauderdale-by-the-Sea (T328, BC1, and FTL4). These three sites are ~12 km from the nearby Hillsboro Inlet, less than 500 m from shore, and have been previously used for benthic and coral monitoring [45–47] within the NFRT (Fig 2). *Montastraea cavernosa* was selected for colony fate-tracking due to the abundance of infected colonies within the study sites. This coral species is considered intermediately susceptible to SCTLD, with onset of tissue loss occurring weeks to months later than what has been reported for highly susceptible species (e.g. *Dendrogyra cylindrus, Meandrina meandrites, Colpophyllia natans*). Lesions on infected

M. cavernosa generally progress slower as compared to highly susceptible coral species, with total mortality occurring within months to years [16]. *Montastraea cavernosa* constituted 11.6% of the reported cases of SCTLD in the NFRT in late 2017 [41].

Twenty-four colonies of *M. cavernosa* affected with SCTLD were tagged with uniquely numbered cattle tags across the three sites on 24-August-2018 (T_1). Sites were revisited on 11-Sep-2018 (T_2), 8-Nov-2018 (T_3), and 17-Dec-2018 (T_4). Continuous video was taken for 3D model generation to quantify total colony surface area and disease lesion area for each time point.

Fate-tracked colonies were filmed using methods outlined in Young et al. [40], with the following modifications: Canon G16 cameras in Fantasea underwater housings were set on "Underwater mode," 1080p and 60 frames per second (fps), and exposure was adjusted as needed based on ambient light conditions. One-meter, L-shaped PVC scale bars marked at 10 cm increments were placed at opposing right angles to frame the designated colony. A SCUBA diver maintained approximately 1 m above the highest point of each coral colony and recorded continuous video while swimming repeated passes in a lawnmower pattern with the camera pointed directly downward. The number of adjacent passes varied depending on colony size, allowing for 60–70% overlap. The camera was rotated 90° at the end of the first set of adjacent passes and another set of passes was completed perpendicular to the first set. The two complete sets of passes for a single colony required between 1–3 min of dive time, depending on colony size. Each sampling event produced an average of 14 GB of .mp4 video files, or approximately 425 MB of video file size per coral colony.

Video processing and 3D model generation protocols are described in full in our GitHub repository

(https://github.com/icombs2017/analysisOf3dModels/tree/master/3D%20Modeling/protocols). In summary, the free software package, *FFmpeg* (www.ffmpeg.org), was used to extract still frame images from videos of the fate-tracked colonies at a rate of 3 fps. Still images were then imported into AgiSoft Metashape Standard Edition (Version 1.5.2, AgiSoft LLC) software, which uses a proprietary algorithm that incorporates SfM and Brown's lens distortion model to generate 3D models from 2D images [30,48]. Model generation in Metashape was conducted according to the manufacturer's protocol in four general steps: 1) camera alignment, 2) dense point cloud generation, 3) mesh generation, and 4) texture overlay. Models were rendered on a 2018 Apple MacBook Pro with a 2.9 GHz processor, 16GB of RAM and a Radeon Pro Vega 16 4GB graphics card. A single model took approximately 40 min to render depending on the number of still images generated. Generated models were then exported as an .obj file and imported into the software Rhinoceros 3D (Robert McNeel & Associates) for analysis; the mean model file size was 64 MB.

Models were scaled using the PVC scale bars, then total colony surface area and disease lesion surface area were measured by hand tracing polygons around coral tissue. SCTLD disease lesion area was defined as the stark white newly-dead coral tissue or skeleton with sloughing tissue that is characteristic of the disease margin. Both total colony area and disease lesion area were generated directly within Rhinoceros 3D, while healthy tissue area was calculated by subtracting disease lesion area from total colony area. Proportion of loss and rate of tissue loss per week were calculated for each pair of time points. To identify significant effects of time on healthy area, diseased area, total area, the proportion of tissue loss and the rate of tissue loss, Friedman's rank sum tests were conducted using the package *PMCMRplus* [49]. Pairwise comparisons were made with Nemenyi tests in the package *PMCMR* [50]. Additionally,

Spearman's rank correlation analyses were used to correlate diseased area and total colony area and rate of tissue loss and total colony area.

3D model validation

Accuracy of model-generated surface area metrics was assessed by a validation experiment using a standardized mock coral colony. A square cupola prism with equal dimensions and surface features was constructed to represent a coral colony using ½" PVC and internal steel rebar to achieve negative buoyancy. The prism was constructed using a top square of 25.4 x 25.4 cm, a bottom square of 47.6 x 47.6 cm, and height of 30.5 cm (S1 Fig). Angled sides were achieved using 45° PVC tees to better represent a mounding coral colony. Additionally, polygons of known surface areas (square: 40.32 cm^2; rectangle: 12.90 cm^2; circle: 45.60 cm^2) were designed using graphics editing software, printed to scale on waterproof paper, and affixed to the top and sloped sides of the weighted prism.

The prism was filmed in a pool and at the three coral fate-tracking sites in Lauderdale-by-the-Sea (Fig 2) to quantify model error in pristine (pool) versus reef conditions (e.g. variable turbidity, currents, surge). Eight videos were recorded for the prism and boundary frames across the three reef sites (2–3 replicates per site), while six prism videos were captured in the pool. The prism was filmed at a depth of 2.75 m in the pool, 8.84 m at BC1, 5.18 m at T328, and 6.71 m at FTL4; filming distance remained the same for all videos. To assess accuracy of surface area measurements, 3D models were generated and analyzed using the same protocols described earlier, with polygons traced for each of the prism shapes based on the four corners for squares and rectangles, and the entire circumference for circles. Replicate surface area measurements of shapes were made for each model according to face of the prism (top and four sides), and

number of shapes on each face (four for square and rectangle, one for circle), for a total $n = 229$ measurements for square, $n = 232$ for rectangle, and $n = 59$ for circle. Shape error was calculated as the absolute difference between measured shape surface areas and the corresponding template area. Shape error measurements were found to be highly right-skewed, therefore a square-root transformation was applied prior to statistical analyses. Multivariate homogeneity of variance was first tested using the *betadisper* function of the package *vegan*, then a single-factor PERMANOVA was conducted across sites using the *adonis* function of the package *vegan*.

Additionally, a subset of four models from the colony fate-tracking dataset were extracted at three different frame rates (3–5 fps) across their respective time points to determine if frame rate affected model accuracy. A Kruskal-Wallis test was used to determine if model size differed across the different extraction frequencies.

Results

Disease prevalence surveys

SCTLD prevalence varied significantly across location (Table 1), but not through time (Table 1, Fig 3). Pairwise comparisons indicated that disease prevalence was lower in JUP and PB than in SLR and LBTS (Table 1, Fig 3). In LBTS, disease prevalence stayed relatively constant (10.9%) while in JUP and PB prevalence was consistently low, but peaked in March 2019 with SCTLD prevalence at 8.1% and 4.3%, respectively. Prevalence data from SLR was highly varied due to generally low coral cover observed during the survey period. For example, the highest reported disease prevalence of 43% at site SLR North was due to low coral cover; only 7 living corals were observed, 3 of which were diseased. The most abundant species throughout our surveys were *M. cavernosa* (63%, $n = 3241$), *Porites astreoides* (17%, $n = 885$),

Siderastrea siderea (4%, *n* = 199), *Stephanocoenia intersepta* (2.3%, *n* = 118), and *Agaricia agaricites* (2.1%, *n* = 108). Species ~~abundance~~ varied among the four locations (Kruskal-Wallis, *H* = 10.308, *p* = 0.016) with species abundance varying at SLR relative to the other three locations (Dunn's Test, all *p* < 0.05). All locations had *M. cavernosa* as the most abundant species, except for SLR which had *P. astreoides* as the most abundant species (65%, *n* = 304). The species with ~~the highest disease prevalence with more than 10 observations~~ were *Pseudodiploria clivosa* (36%, *n* = 47), *A. agaricites* (10%, *n* = 108), *Dichocoenia stokesii* (9%, *n* =23), *Orbicella faveolata* (6%, *n* = 49) and *M. cavernosa* (5%, *n* = 3241).

Table 1. Results from univariate-permutational analysis of variance (PERMANOVA) comparing SCTLD prevalence from the roving diver surveys across location and time. Pairwise comparisons from results of the univariate PERMANOVA across all four locations: St. Lucie Reef (SLR), Jupiter (JUP), Palm Beach (PB), and Lauderdale-by-the-Sea (LBTS).

Test	Comparison	df	Psuedo-*F*	*p*-value*
PERMANOVA	Location	3	7.44	< 0.001
	Date	12	1.23	ns
	Location:Date	7	0.52	ns
Pairwise	LBTS – SLR		0.11	ns
	LBTS – JUP		26.76	< 0.001
	LBTS – PB		35.91	< 0.001
	SLR – JUP		7.12	0.0492
	SLR – PB		7.88	0.045
	JUP – PB		0.63	ns

*Non-significant *p* values listed as "ns".

Fig 3. Mean SCTLD prevalence across all four locations (St. Lucie Reef, Jupiter, Palm Beach and Lauderdale-by-the-Sea). Points represent means; bars represent standard error.

Fate-tracking of SCTLD-affected *M. cavernosa*

Friedman's rank sum tests indicated a significant decrease in total colony area (m^2) and healthy tissue area over time (Table 2, Fig 4). Disease lesion area significantly varied through time, however pairwise comparisons revealed that one time point comparison (T_2–T_4) was driving the variation (Table 2).

Table 2. Results of Friedman's rank sum tests comparing total colony area, disease tissue area and healthy tissue area across time, with pairwise comparisons made using Nemenyi tests.

Test	Comparison	Test Statistic	*p* value*
Total Colony Area	Friedman's	48.55	< 0.001
	$T_1 - T_2$	4.27	ns
	$T_1 - T_3$	7.59	< 0.001
	$T_1 - T_4$	9.01	< 0.001
	$T_2 - T_3$	3.32	ns
	$T_2 - T_4$	4.74	0.026
	$T_3 - T_4$	1.42	ns
Healthy Tissue Area	Friedman's	41.29	< 0.001
	$T_1 - T_2$	4.27	0.026
	$T_1 - T_3$	7.59	< 0.001
	$T_1 - T_4$	9.01	< 0.001
	$T_2 - T_3$	3.32	ns
	$T_2 - T_4$	4.74	ns
	$T_3 - T_4$	1.42	ns
Disease Lesion Area	Friedman's	14.02	0.003

Test	Comparison	Test Statistic	*p* value*
	$T_1 – T_2$	4.27	ns
	$T_1 – T_3$	7.59	ns
	$T_1 – T_4$	9.01	ns
	$T_2 – T_3$	3.32	ns
	$T_2 – T_4$	4.74	0.012
	$T_3 – T_4$	1.42	ns

T_1, 24-Aug-2018; T_2, 11-Sep-2018; T_3, 8-Nov-2018; T_4, 17-Dec-2018.

*Non-significant *p* values listed as “ns”.

Fig 4. Comparison of tissue areas through time. Mean tissue areas through time (a) mean total colony area (cm^2) (b) mean healthy tissue area (cm^2), and (c) mean disease lesion area across all sites and through time for fate-tracked *M. cavernosa* colonies (n = 24). T_1, 24-Aug-2018; T_2, 11-Sep-2018; T_3, 8-Nov-2018; T_4, 17-Dec-2018.

A Friedman’s rank sum test revealed significant differences between rates of tissue loss through time ($df_{2,71}$, Friedman $\chi^2 = 6.25$, $p = 0.044$). Average tissue loss between T_1–T_2 was -98.17 ± 40.74 cm^2 wk^{-1}, -24.99 ± 7.70 cm^2 wk^{-1} between T_2–T_3, and -45.10 ± 19.64 cm^2 wk^{-1} between T_3–T_4 (Fig 5a). Rate of tissue loss was not significantly different among sites between any time point (S1 Table). On average, the proportion of tissue lost was 37.1 ± 7.2% over the course of this study, with three colonies experiencing complete mortality. There was no correlation between total colony area and disease lesion area (Spearman’s rank correlation r_s = 0.15, p = 0.154, Fig 6), or between rates of tissue loss and total colony area (Spearman’s rank correlation r_s = -0.046, p = 0.704, Fig 5b).

Fig 5. Comparison of rates of tissue loss. (a) Mean change in tissue area on fate-tracked *M. cavernosa* colonies and (b) Spearman's rank correlation between rate of tissue loss ($cm^2 wk^{-1}$) and total colony area (cm^2).

Fig 6. Correlations between total colony area and disease lesion area. Spearman's rank correlation between disease lesion area (cm^2) and total colony area (cm^2) for fate-tracked *M. cavernosa* colonies (n = 24).

3D model validation

Assessment of multivariate variance identified heterogeneous variance in shape surface area error among sites due primarily to lower variance at site FTL4. A PERMANOVA determined that shape error did not vary across the pool and three coral fate-tracking sites in Lauderdale-by-the-Sea ($F_{3,279} = 0.733$, $p = 0.584$). The mean shape error across all sites was 1.70 ± 0.09 cm^2 (pool: 1.37 ± 0.10 cm^2; BC1: 1.97 ± 0.23 cm^2; T328: 2.38 ± 0.30 cm^2; FTL4: 1.82 ± 0.18 cm^2), which corresponded to an error rate of 6.13 ± 0.27% (pool: 4.87 ± 0.32%; BC1: 7.02 ± 0.67%; T328: 8.72 ± 0.92%; FTL4: 6.87 ± 0.57%).

To assess potential effects of different frame rates used during model generation on colony-scale surface area measurements, a single-factor Kruskal-Wallis test indicated that frame rate had no significant impact on total colony area (Kruskal-Wallis, H = 0.12, $p = 0.94$). This suggests that higher frame rates can be used from the initial video to improve poorly constructed models without affecting downstream spatial analyses. To keep processing time as low as possible, however, stills were extracted at 3fps unless otherwise necessary.

Discussion

Disease prevalence surveys

SCTLD is a unique coral disease due to its geographic extent, the number of coral species affected, and rapid rates of disease progression and spread [16,17,51,52]. SCTLD has been reported across ~400 km of the FRT, and now has been observed in at least 12 other territories throughout the TWA [20]. Other coral diseases, such as white syndrome, have been observed over broader spatial extents (~1500 km) in the Great Barrier Reef [53]. Likewise, black band disease has been observed on coral reefs across multiple oceans [54]. The geographic extent of SCTLD continues to expand with new observations throughout the Caribbean [19,20]. SCTLD has been reported as far west as Belize and as far southeast as Martinique which are 1300 and 2500 km respectively from the northern terminus of the NFRT at St. Lucie Reef, FL [20]. The overall mean disease prevalence for the NFRT observed in this study was 6%, which is lower than recorded in post Hurricane Irma surveys in 2017 [41] and previous SCTLD surveys in SE Florida [51]. Sites in Jupiter and Palm Beach had relatively low disease prevalence (1.7% and 1.0%) consistent with background disease levels (1–3%) within the TWA [55]. The highest values observed in this study were between 21–43% at SLR, but no sites reached the highest reported disease prevalence values of 60% observed near Miami [17]. The lower prevalence values reported in this study may be due in part to differences in species composition between the NFRT and southern regions of the FRT. The most abundant and susceptible species such as *M. meandrites*, *D, stokesi*, and *Pseudodiploria strigosa* [17,51] were comparatively absent within our NFRT surveys. However, previous SCTLD impacts before our monitoring sites were established may also have affected the relative abundance of susceptible species [51,56].

We observed an increase in disease prevalence during the spring of 2018 which was unexpected, as prevalence for other described coral diseases often increases during the summer months as water temperatures increase [57–60]. SCTLD prevalence does not appear to have a positive correlation with temperature [17,51] as has been observed for other coral diseases [58,61,62], but potential environmental cofactors that may drive SCTLD prevalence need to be examined further.

Fate-tracking of SCTLD-affected *M. cavernosa*

At the colony level, disease progression was highly variable across fate-tracked *M. cavernosa* colonies near Lauderdale-by-the-Sea. As expected, total colony area and healthy tissue area decreased significantly over time, demonstrating the impact SCTLD can have on living coral cover over just a few months. Over the course of fate-tracking period (115 days), colonies lost on average 37.1 ± 7.24% of their tissue surface area. In contrast, Aeby et al. [24] noted subacute tissue loss in *M. cavernosa* was 34 ± 8.7% over 1 year using a semi-quantitative method to estimate tissue. Additionally, using a similar 3D photogrammetry method, Meiling et al. [63] saw proportional losses of 2.0 ± 0.11% day^{-1} of six *M. cavernosa* colonies in the US Virgin Islands.

Disease lesion area did not correlate with total colony area, suggesting that larger colonies do not exhibit a higher proportion of diseased tissue compared to smaller colonies. As SCTLD progresses on coral colonies, loss of healthy tissue appears to be a more important indicator of disease virulence, as opposed to change in disease lesion area. Importantly, rates of tissue loss did not correlate with total colony area. Therefore, larger corals may have a more favorable time horizon for potential intervention actions that could prevent total colony

mortality. Conversely, smaller infected colonies may succumb more quickly to SCTLD. Considering the level of effort required for SCTLD interventions [64], prioritizing interventions on larger colonies with sufficient areas of uninfected tissue remaining may result in more successful disease mitigation.

3D model generation as a fate-tracking method

This study tested a relatively inexpensive and rapid 3D model generation methodology described in Young et al. [40] as a means to track disease progression in entire coral colonies without substantial increases in expense, time, or computational requirements compared to observational and photographic techniques. Surface areas of complex coral morphologies were calculated to generate data on live versus dead coral tissue over time. Additionally, this 3D modeling technique was validated through the quantification of model error using a mock coral colony with standardized surface shapes. Shape error (i.e. variation between measured surface areas and template shapes) did not vary across a pool and the three coral fate-tracking sites in Lauderdale-by-the-Sea (S2 Fig). Global shape error across all sites was 1.70 ± 0.09 cm^2, which corresponded to an error rate of 6.13 ± 0.27%. This is likely an inflated estimate of model error, as the prism shapes used for accuracy assessments were printed on waterproof paper that could move with currents, rather than fixed in place as are scleractinian corals. There were some instances, however, where model generation was poor due to anomalies in the Metashape algorithm, inconsistent underwater filming, or most ~~common~~, high turbidity, all of which may impact surface area measurements and therefore model error. Typically, these issues could be rectified by extracting still images from the video at an increased frame rate to ensure higher overlap among images, or by collecting replicate videos for each target. Notably, extracting at a

higher fps improved poor models, but did not disproportionately affect surface area measurements from models that were constructed well using images extracted at 3 fps.

Due to Metashape's proprietary algorithm, further adjustments within the model generation process to rectify poor models are limited. Stable, high-resolution image collection is therefore integral to successful downstream model generation. Alternatives to the lawn-mower video path used in this study may be more effective but also more time-intensive, particularly for tall (>1 m), highly-rugose coral colonies. Discrete, overlapping photographs could be taken instead of continuous video [36], or video could be taken while swimming a circular pattern around the coral at varying angles [65]. Critically, while disease monitoring via linear extension measurements may be able determine potential differences among colonies or treatments over time [16,27,66], linear extension does not accurately quantify tissue loss and may underestimate the impacts of disease lesions on coral colonies. Therefore, 3D approaches, such as the method presented here, should be employed to quantify lost coral surface area and to identify colonies for which intervention efforts may be successful.

Conclusions

SCTLD is particularly non-discriminant compared to other coral diseases relative to its host specificity and seasonal distribution. Given this study's comparisons between tissue loss and coral colony sizes, intervention efforts on smaller colonies with numerous active lesions may be less efficient. In instances where time, personnel, weather opportunities, or boating and diving resources are limited, the results of this study suggest that larger, longer-lived colonies should be prioritized when attempting to limit the impacts of SCTLD. Since SCTLD is a progressive, necrotic infection, the area of tissue loss, or proportion of tissue loss, may represent alternative,

more impactful metrics for quantifying the severity of infection as opposed to disease lesion area or percent affected tissue. Additional studies incorporating longer timescales, multiple species, and similar quantitative 3D metrics are warranted to confirm these patterns of disease progression across all susceptible species. Rapid, cost-effective, and accurate methods using 3D models for quantifying coral surface area are quite feasible so long as the appropriate redundancies and precautions are followed. It is recommended that managers and intervention specialists—particularly those focusing on SCTLD—adopt photogrammetric methods to ensure that data are more accurate and comparable across future studies.

Data availability

Model generation protocol, analysis scripts, and documentation are available on GitHub (https://github.com/icombs2017/analysisOf3dModels). Disease prevalence observations can be found in the S1 Dataset. Surface area measurements from the fate-tracked coral colonies can be found in the S2 Dataset. Surface area and error measurements for the three prism shapes can be found in S3 Dataset.

Acknowledgements

We thank J. Nelson and M. Roy for assistance with boating and diving operations; J. Beal, E. Shilling, and A. Sturm for assistance with data collection. K. Kerrigan, J. Walczak, B. Walker, and K. Neely provided valuable feedback on the study design.

References

1. Gardner TA, Côté IM, Gill JA, Grant A, Watkinson AR. Long-term region-wide declines in Caribbean corals. Science (80-). 2003;301: 958–960. doi:10.1126/science.1086050
2. Mumby PJ. The Impact of Exploiting Grazers (Scaridae) on the Dynamics of Caribbean Coral Reefs Author (s): Peter J . Mumby Published by : Wiley Stable URL : http://www.jstor.org/stable/40061693 Accessed : 05-04-2016 08 : 06 UTC Your use of the JSTOR archive ind. 2016;16: 747–769.
3. Richardson LL. Coral diseases: What is really known? Trends Ecol Evol. 1998;13: 438–443. doi:10.1016/S0169-5347(98)01460-8
4. Aronson RB, Precht WF. White-band disease and the changing face of Caribbean coral reefs. Hydrobiologia. 2001;460: 25–38. doi:10.1023/A:1013103928980
5. Patterson KL, Porter JW, Ritchie KB, Polson SW, Mueller E, Peters EC, et al. The etiology of white pox, a lethal disease of the Caribbean elkhorn coral, Acropora palmata. Proc Natl Acad Sci U S A. 2002;99: 8725–8730. doi:10.1073/pnas.092260099
6. Miller J, Muller E, Rogers C, Waara R, Atkinson A, Whelan KRT, et al. Coral disease following massive bleaching in 2005 causes 60% decline in coral cover on reefs in the US Virgin Islands. Coral Reefs. 2009;28: 925–937. doi:10.1007/s00338-009-0531-7
7. Nugues MM. Impact of a coral disease outbreak on coral communities in St Lucia. 2002;229: 61–71.
8. Miller MW, Williams DE. Coral disease outbreak at Navassa, a remote Caribbean island. Coral Reefs. 2007;26: 97–101. doi:10.1007/s00338-006-0165-y
9. Richardson LL, Goldberg WM, Carlton RG, Halas JC. Coral disease outbreak in the Florida Keys: Plague Type II. Rev Biol Trop. 1998;46: 187–198. Available:

http://www.amlc-carib.org/meetings/procs/1997AMLC_Proceedings/richardson.pdf

10. McClanahan TR, Muthiga NA. An ecological shift in a remote coral atoll of Belize over 25 years. Environ Conserv. 1998;25: 122–130. doi:10.1017/S0376892998000174

11. McManus JW, Polsenberg JF. Coral-algal phase shifts on coral reefs: Ecological and environmental aspects. Prog Oceanogr. 2004;60: 263–279. doi:10.1016/j.pocean.2004.02.014

12. Graham NAJ, Wilson SK, Jennings S, Polunin NVC, Robinson J, Bijoux JP, et al. Lag effects in the impacts of mass coral bleaching on coral reef fish, fisheries, and ecosystems. Conserv Biol. 2007;21: 1291–1300. doi:10.1111/j.1523-1739.2007.00754.x

13. Smith S V. Coral-reef area and the contributions of reefs to processes and resources of the world's oceans. Nature. 1978;273: 225–226. doi:10.1038/273225a0

14. Sheppard C, Dixon DJ, Gourlay M, Sheppard A, Payet R. Coral mortality increases wave energy reaching shores protected by reef flats: Examples from the Seychelles. Estuar Coast Shelf Sci. 2005;64: 223–234. doi:10.1016/j.ecss.2005.02.016

15. Yamamuro M, Kayanne H, Minagawao M. Carbon and nitrogen stable isotopes of primary producers in coral reef ecosystems. Limnol Oceanogr. 1995;40: 617–621. doi:10.4319/lo.1995.40.3.0617

16. NOAA. Stony Coral Tissue Loss Disease Case Definition. Florida Keys Natl Mar Sanctuary. Silver Spring, MD; 2018. Available: https://nmsfloridakeys.blob.core.windows.net/floridakeys-prod/media/docs/20181002-stony-coral-tissue-loss-disease-case-definition.pdf

17. Precht WF, Gintert BE, Robbart ML, Fura R, Van Woesik R. Unprecedented Disease-Related Coral Mortality in Southeastern Florida. Sci Rep. 2016;6: 31374.

doi:10.1038/srep31374

18. Cunning R, Silverstein RN, Barnes BB, Baker AC. Extensive coral mortality and critical habitat loss following dredging and their association with remotely-sensed sediment plumes. Mar Pollut Bull. 2019;145: 185–199. doi:10.1016/j.marpolbul.2019.05.027

19. Alvarez-Filip L, Estrada-Saldívar N, Pérez-Cervantes E, Molina-Hernández A, González-Barrios FJ. A rapid spread of the stony coral tissue loss disease outbreak in the Mexican Caribbean. PeerJ. 2019;2019. doi:10.7717/peerj.8069

20. Roth L, Kramer PR, Doyle E, O’Sullivan C. Caribbean SCTLD Dashboard. 2020 [cited 6 Apr 2020]. Available: www.agrra.org

21. Landsberg JH, Kiryu Y, Peters EC, Wilson PW, Perry N, Waters Y, et al. Stony Coral Tissue Loss Disease in Florida Is Associated With Disruption of Host–Zooxanthellae Physiology. Front Mar Sci. 2020;7: 1–24. doi:10.3389/fmars.2020.576013

22. Meyer JL, Castellanos-Gell J, Aeby GS, Häse CC, Ushijima B, Paul VJ. Microbial Community Shifts Associated With the Ongoing Stony Coral Tissue Loss Disease Outbreak on the Florida Reef Tract. Front Microbiol. 2019;10: 2244. doi:10.3389/fmicb.2019.02244

23. Rosales SM, Clark AS, Huebner LK, Ruzicka RR, Muller EM. Rhodobacterales and Rhizobiales Are Associated With Stony Coral Tissue Loss Disease and Its Suspected Sources of Transmission. Front Microbiol. 2020;11: 1–20. doi:10.3389/fmicb.2020.00681

24. Aeby GS, Ushijima B, Campbell JE, Jones S, Williams GJ, Meyer JL, et al. Pathogenesis of a Tissue Loss Disease Affecting Multiple Species of Corals Along the Florida Reef Tract. Front Mar Sci. 2019;6: 678. doi:10.3389/fmars.2019.00678

25. Neely K. Ex-Situ Disease Treatment Trials Completed in Fulfillment of PO B2B8A4 and

PO B30DB3 for. 2018.

26. Neely K, Hower E. FY 2018 In-Situ Disease Intervention. 2018. Available: https://floridadep.gov/sites/default/files/Large-Coral-Disease-Intervention-Summary-Report.pdf

27. Voss JD, Richardson LL. Nutrient enrichment enhances black band disease progression in corals. Coral Reefs. 2006;25: 569–576. doi:10.1007/s00338-006-0131-8

28. Dalton SJ, Smith SDA. Coral disease dynamics at a subtropical location, Solitary Islands Marine Park, eastern Australia. Coral Reefs. 2006;25: 37–45. doi:10.1007/s00338-005-0039-8

29. Courtney LA, Fisher WS, Raimondo S, Oliver LM, Davis WP. Estimating 3-dimensional colony surface area of field corals. J Exp Mar Bio Ecol. 2007;351: 234–242. doi:10.1016/j.jembe.2007.06.021

30. Westoby MJ, Brasington J, Glasser NF, Hambrey MJ, Reynolds JM. "Structure-from-Motion" photogrammetry: A low-cost, effective tool for geoscience applications. Geomorphology. 2012;179: 300–314. doi:10.1016/j.geomorph.2012.08.021

31. Harborne AR, Mumby PJ, Ferrari R. The effectiveness of different meso-scale rugosity metrics for predicting intra-habitat variation in coral-reef fish assemblages. Environ Biol Fishes. 2012;94: 431–442. doi:10.1007/s10641-011-9956-2

32. Burns JHR, Delparte D, Gates RD, Takabayashi M. Utilizing underwater three-dimensional modeling to enhance ecological and biological studies of coral reefs. Int Arch Photogramm Remote Sens Spat Inf Sci - ISPRS Arch. 2015;40: 61–66. doi:10.5194/isprsarchives-XL-5-W5-61-2015

33. Leon JX, Roelfsema CM, Saunders MI, Phinn SR. Measuring coral reef terrain roughness

using “Structure-from-Motion” close-range photogrammetry. Geomorphology. 2015;242: 21–28. doi:10.1016/j.geomorph.2015.01.030

34. Cocito S, Sgorbini S, Peirano A, Valle M. 3-D reconstruction of biological objects using underwater video technique and image processing. J Exp Mar Bio Ecol. 2003;297: 57–70. doi:10.1016/S0022-0981(03)00369-1

35. Gutiérrez-Heredia L, D’Helft C, Reynaud EG. Simple methods for interactive 3D modeling, measurements, and digital databases of coral skeletons. Limnol Oceanogr Methods. 2015;13: 178–193. doi:10.1002/lom3.10017

36. Ferrari R, Figueira WF, Pratchett MS, Boube T, Adam A, Kobelkowsky-Vidrio T, et al. 3D photogrammetry quantifies growth and external erosion of individual coral colonies and skeletons. Sci Rep. 2017;7: 1–9. doi:10.1038/s41598-017-16408-z

37. Lange ID, Perry CT. A quick, easy and non-invasive method to quantify coral growth rates using photogrammetry and 3D model comparisons. Methods Ecol Evol. 2020;11: 714–726. doi:10.1111/2041-210X.13388

38. Bythell J, Pan P, Lee J, J. B, P. P, J. L, et al. Three-dimensional morphometric measurements of reef corals using underwater photogrammetry techniques. Coral Reefs. 2001;20: 193–199. doi:10.1007/s003380100157

39. Lavy A, Eyal G, Neal B, Keren R, Loya Y, Ilan M. A quick, easy and non-intrusive method for underwater volume and surface area evaluation of benthic organisms by 3D computer modelling. Methods Ecol Evol. 2015;6: 521–531. doi:10.1111/2041-210X.12331

40. Young GC, Dey S, Rogers AD, Exton D. Correction: Cost and time-effective method for multi-scale measures of rugosity, fractal dimension, and vector dispersion from coral reef

3D models (PLoS ONE (2018)12: 4 (e0175341) DOI : 10.1371/journal.pone.0175341). Metze K, editor. PLoS One. 2018;13: 1–18. doi:10.1371/journal.pone.0201847

41. Walker BK. Southeast Florida reef-wide Post-Irma coral disease surveys. 2018. Available: https://floridadep.gov/sites/default/files/Southeast-Florida-Reef-Wide-Post-Irma-Coral-Disease-Surveys_0.pdf

42. R Core Team. A Language and Environment for Statistical Computing. R Foundation for Statistical Computing. Vienna, Austria; 2020. p. https://www.R-project.org. Available: http://www.r-project.org

43. Oksanen AJ, Blanchet FG, Kindt R, Legen- P, Minchin PR, Hara RBO, et al. Community Ecology Package. … Ecology Package …. 2012. p. 263. doi:10.4135/9781412971874.n145

44. Martinez Arbizu P. pairwiseAdonis: Pairwise multilevel comparison using adonis. R package version 0.4. 2020.

45. Gilliam DS, Walton CJ, Brinkhuis V, Ruzicka R, Colella M. Southeast Florida Coral Reef Evaluation and Monitoring Project 2014, Year 12. Florida DEP Report #RM085. 2015; 43. Available: https://floridadep.gov/sites/default/files/SECREMP_2014.pdf

46. Jones NP. Spatiotemporal change in the benthic community of southeast Florida. 2018; 1–60. Available: https://nsuworks.nova.edu/occ_stuetd

47. Walker BK, Brunelle A. Southeast Florida large (> 2 meter) diseased coral colony intervention summary report Florida Department of Environmental Protection Coral Reef Conservation Program Florida Fish and Wildlife Conservation Commission Southeast Florida large (> 2 meter). 2018.

48. Brown DC. Close-range camera calibration. Symp Close-Range Photogramm. 1971;37:

855–866. Available: https://www.asprs.org/wp-content/uploads/pers/1971journal/aug/1971_aug_855-866.pdf

49. Pohlert T. PMCMRplus: Calculate pairwise comparisons of mean rank sums. 2020. Available: https://cran.r-project.org/package=PMCMRplus

50. Pohlert T. The Pairwise Multiple Comparison of Mean Ranks Package (PMCMR). R package. 2014. p. 27. Available: http://cran.r-project.org/package=PMCMR

51. Walton CJ, Hayes NK, Gilliam DS. Impacts of a regional, multi-year, multi-species coral disease outbreak in Southeast Florida. Front Mar Sci. 2018;5: 323. doi:10.3389/fmars.2018.00323

52. Nathan AJ, Scobell A. How China sees America. Foreign Aff. 2012. doi:10.1017/CBO9781107415324.004

53. Selig ER, Drew Harvell C, Bruno JF, Willis BL, Page CA, Casey KS, et al. Analyzing the relationship between ocean temperature anomalies and coral disease outbreaks at broad spatial scales. 2011; 111–128. doi:10.1029/61ce07

54. Frias-Lopez J, Bonheyo GT, Jin Q, Fouke BW. Cyanobacteria associated with coral black band disease in Caribbean and Indo-Pacific reefs. Appl Environ Microbiol. 2003;69: 2409–2413. doi:10.1128/AEM.69.4.2409-2413.2003

55. Weil E, Cróquer A, Urreiztieta I. Yellow band disease compromises the reproductive output of the Caribbean reef-building coral Montastraea faveolata (Anthozoa, Scleractinia). Dis Aquat Organ. 2009;87: 45–55. doi:10.3354/dao02103

56. Beal J, Voss J, Edge S, Cohen L. Assessment of coral stressors on St. Lucie Reef: Florida’s northernmost coral reef. Final Rep State Wildl Grant USFWS T-91-1. 2012. Available:

http://fau.digital.flvc.org/islandora/object/fau%3A2737/datastream/OBJ/view/Assessment_of_coral_stressors_on_St__Lucie_reef__Florida___s_northernmost_coral_reef.pdf

57. Ben-Haim Y, Zicherman-Keren M, Rosenberg E. Temperature-regulated bleaching and lysis of the coral Pocillopora damicomis by the novel pathogen Vibrio coralliilyticus. Appl Environ Microbiol. 2003;69: 4236–4242. doi:10.1128/AEM.69.7.4236-4242.2003

58. Bruno JF, Selig ER, Casey KS, Page CA, Willis BL, Harvell CD, et al. Thermal stress and coral cover as drivers of coral disease outbreaks. Roberts C, editor. PLoS Biol. 2007;5: 1220–1227. doi:10.1371/journal.pbio.0050124

59. Voss JD, Richardson LL. Coral diseases near Lee Stocking Island, Bahamas: Patterns and potential drivers. Dis Aquat Organ. 2006;69: 33–40. doi:10.3354/dao069033

60. Zvuloni A, Artzy-Randrup Y, Katriel G, Loya Y, Stone L. Modeling the Impact of White-Plague Coral Disease in Climate Change Scenarios. PLoS Comput Biol. 2015;11: 1–22. doi:10.1371/journal.pcbi.1004151

61. Harvell DC, Jordán-Dahlgren E, Merkel S, Rosenberg E, Raymundo LJ, Smith GW, et al. Coral disease, environmental drivers, and the balance between coral and microbial associates. Oceanography. 2007;20: 172–195. doi:10.5670/oceanog.2007.91

62. Roberts KE, Hadfield JD, Sharma MD, Longdon B. Changes in temperature alter the potential outcomes of virus host shifts. PLoS Pathog. 2018;14: 1–16. doi:10.1371/journal.ppat.1007185

63. Meiling S, Muller EM, Smith TB, Brandt ME. 3D Photogrammetry Reveals Dynamics of Stony Coral Tissue Loss Disease (SCTLD) Lesion Progression Across a Thermal Stress Event. Front Mar Sci. 2020;7. doi:10.3389/fmars.2020.597643

64. Neely K. Florida Keys Coral Disease STrike Team: FY 2019/2020 Final Report. Miami,

FL; 2020.

65. Figueira W, Ferrari R, Weatherby E, Porter A, Hawes S, Byrne M. Accuracy and precision of habitat structural complexity metrics derived from underwater photogrammetry. Remote Sens. 2015;7: 16883–16900. doi:10.3390/rs71215859

66. Brandt ME, Smith TB, Correa AMS, Vega-Thurber R. Disturbance Driven Colony Fragmentation as a Driver of a Coral Disease Outbreak. Harder T, editor. PLoS One. 2013;8: 1–10. doi:10.1371/journal.pone.0057164

Supporting information

S1 Table. Kruskal-Wallis test comparing rate of tissue loss and site. Non-significant *p* values are listed as "ns".

S1 Fig. Panel of prism template and deployment for underwater 3D photogrammetry. A) Template used for five sides of prism, scaled to produce replicates of three standardized shapes: square (40.32 cm^2), rectangle (12.90 cm^2), and circle (45.60 cm^2) simulating surface area measurements on a coral colony. B) Deployment of prism and scaling frames with diver recording continuous video in a lawnmower-pattern at an approximate distance of 1 m. C) Overhead view of prism and scaling frames, with 10 cm banded tape for scaling. D) Deployment of prism and scaling frames on a reef environment at Lauderdale-by-the-Sea, FL.

S2 Fig. Panel of error measurements for each of the three prism shapes (square, rectangle, circle), calculated as the absolute difference between measured shape surface areas and template areas.

S1 Dataset. Dataset containing disease prevalence data from roving diver disease surveys.

S2 Dataset. Dataset containing tissue area measurements from rendered 3D models of fate-tracked *Montastraea cavernosa* colonies.

S3 Dataset. Dataset containing shape surface area and error measurements from prism deployment across three sites (pool, Lauderdale-by-the-Sea, St. Lucie Reef). Error was calculated as the absolute difference between measured surface areas and actual area of the template shapes, then standardized as a percentage relative to template area.

Fig 1

Click here to access/download;Figure;Fig1.tif

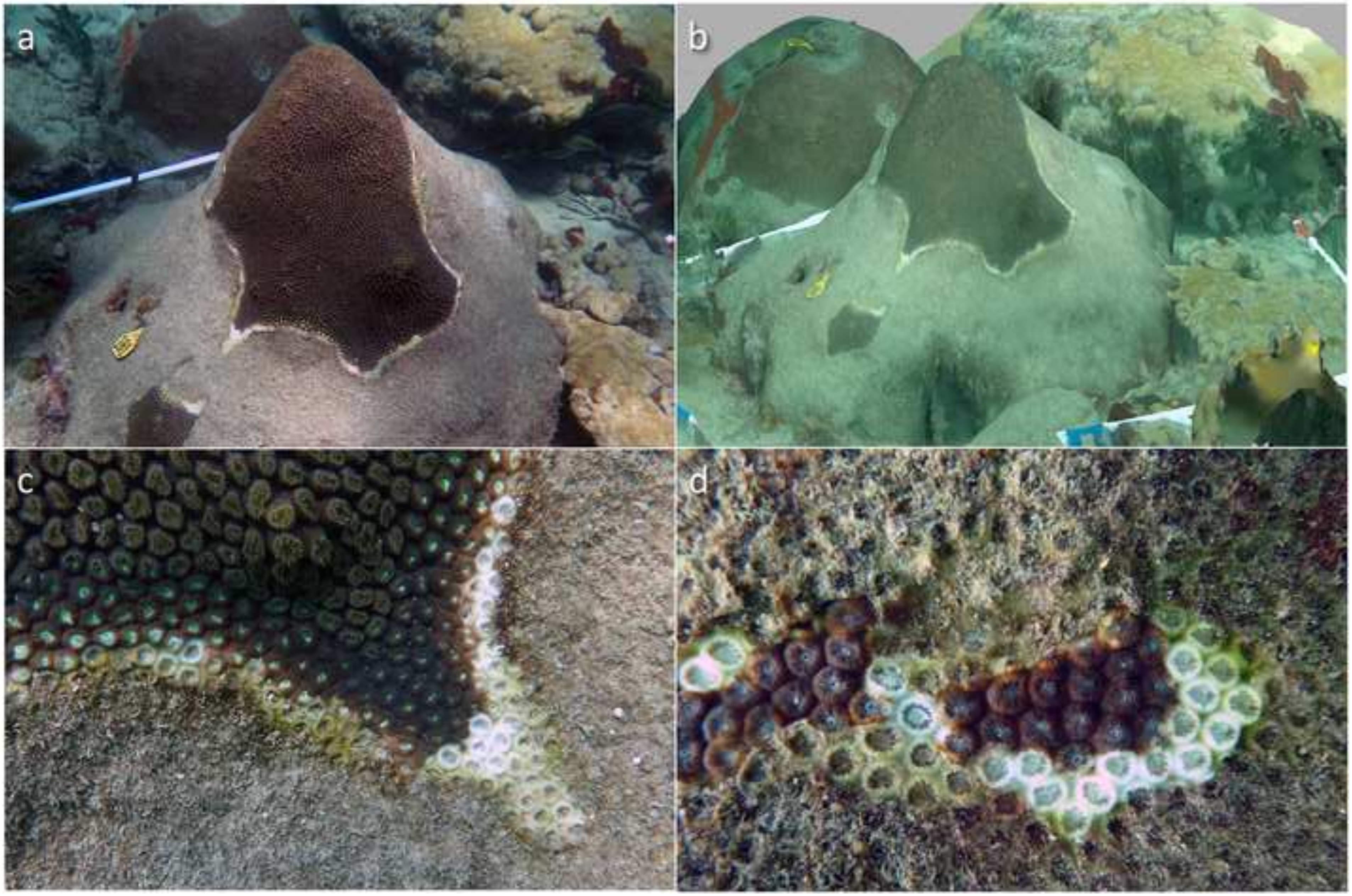

Fig 2

Click here to access/download;Figure;Fig2.tif

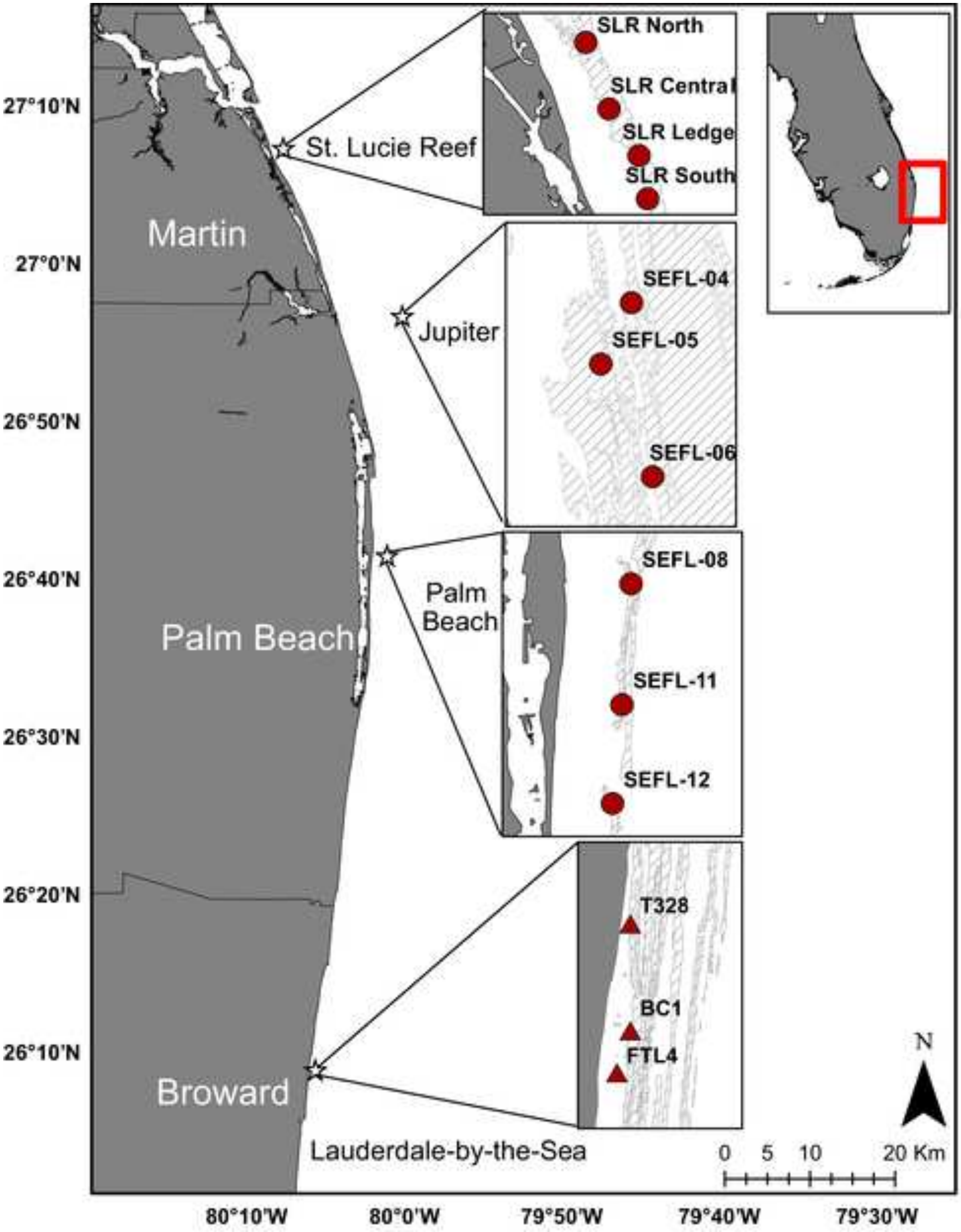

Fig 3

Click here to access/download;Figure;Fig3.tif

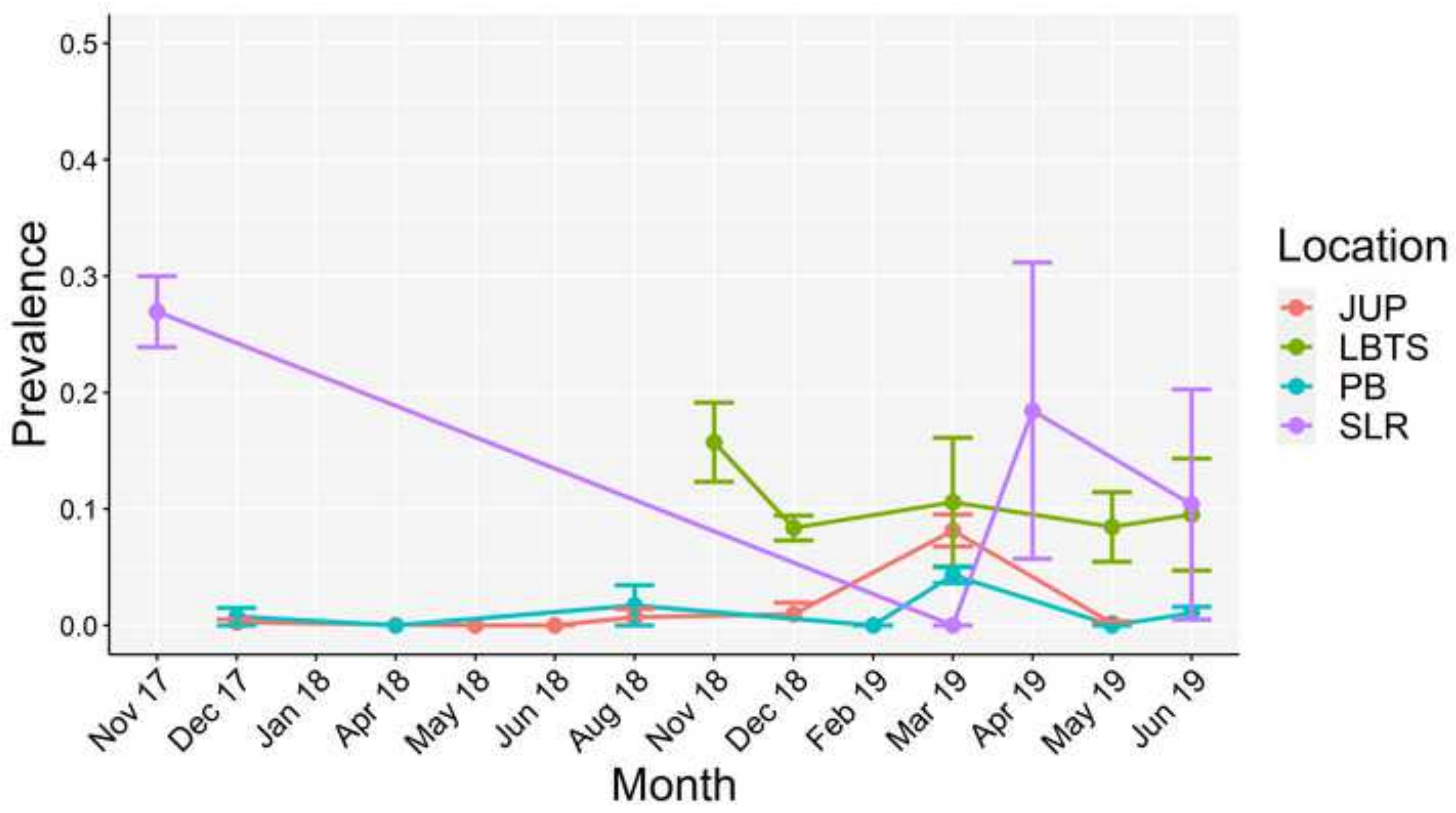

Fig 4

Click here to access/download;Figure;fig4.tif

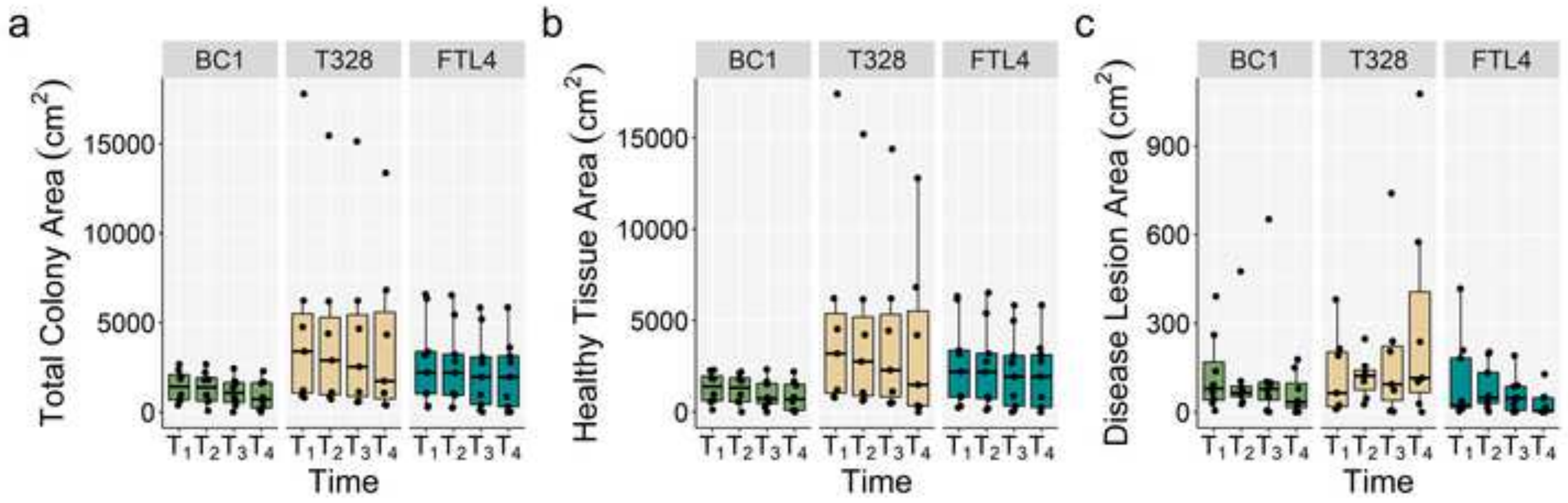

Fig 5

Click here to access/download;Figure;Fig5.tif

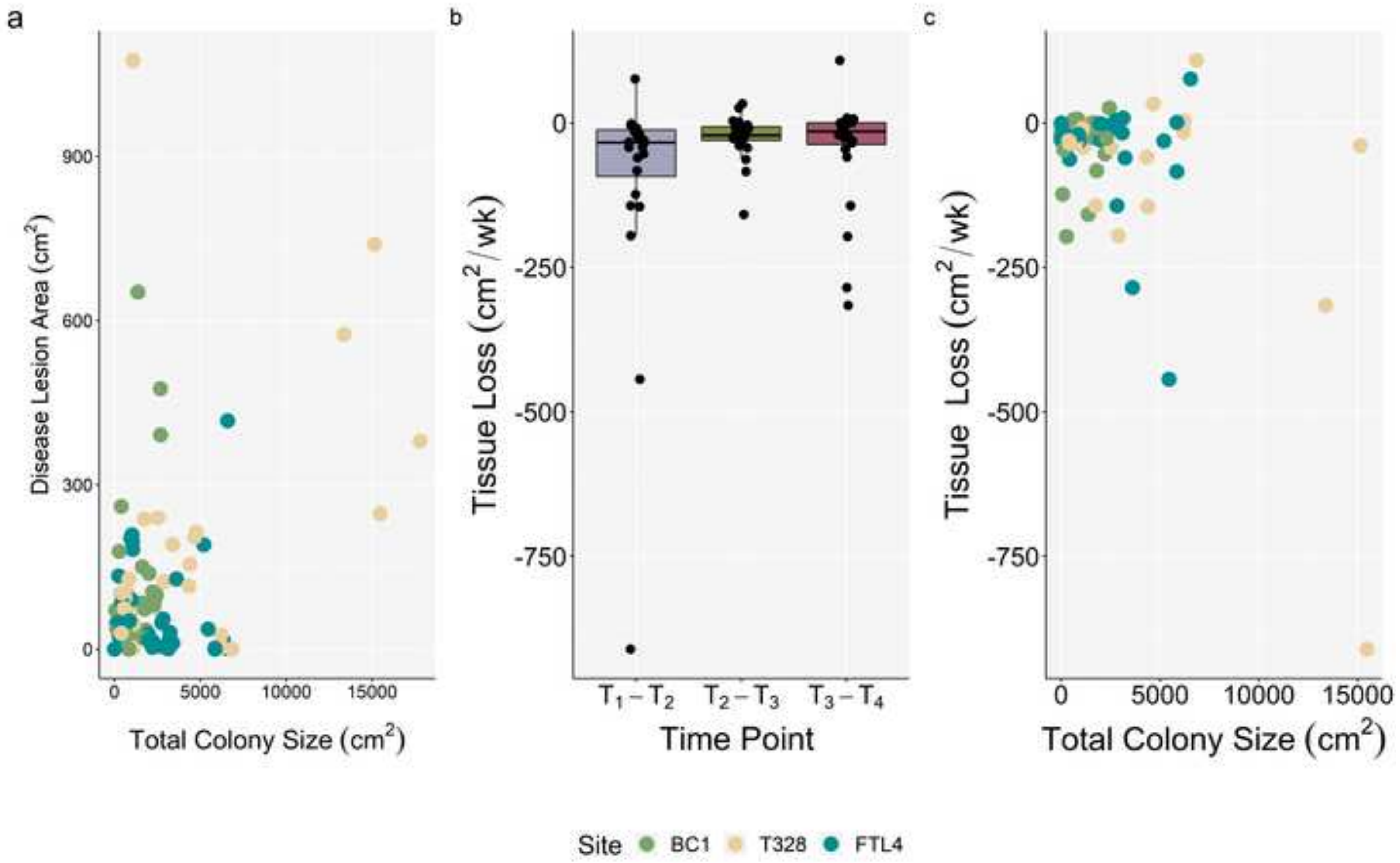

Fig 6

Click here to access/download;Figure;Fig6.tif

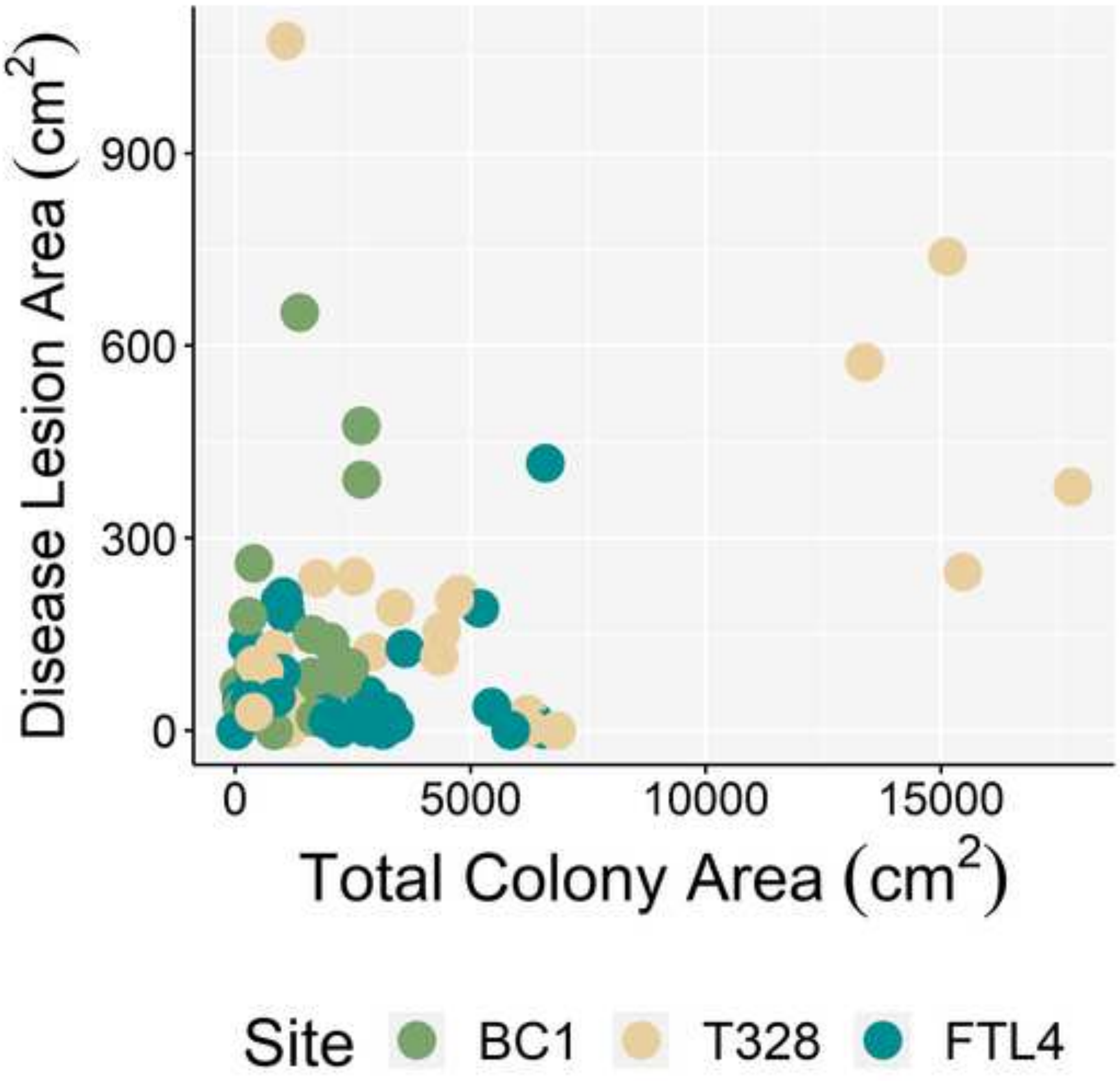

S1 Table

Click here to access/download
Supporting Information
S1_Table.docx

S1 Fig

Click here to access/download
Supporting Information
S1_Fig.tif

S2 Fig

Click here to access/download
Supporting Information
S2_Fig.tif

S1 Data set

Click here to access/download

Supporting Information

S1_Dataset.xlsx

S2 Data set

Click here to access/download
Supporting Information
S2_Dataset.xlsx

S3 Data set

Click here to access/download
Supporting Information
S3_Dataset.xlsx

Quantifying impacts of stony coral tissue loss disease on corals in Southeast Florida through surveys and 3D photogrammetry

Ian R Combs[1,2*], Michael S Studivan[1,3], Ryan J Eckert[1], Joshua D Voss[1]

[1] Department of Biological Sciences, Harbor Branch Oceanographic Institute, Florida Atlantic University, Fort Pierce, Florida, United States of America

[2] Current address: Elizabeth Moore International Center for Coral Reef Research & Restoration, Mote Marine Laboratory, Summerland Key, Florida, United States of America

[3] Current address: Cooperative Institute for Marine and Atmospheric Studies, University of Miami, Miami, Florida, United States of America

* combsi9892@gmail.com

Abstract

Since 2014, stony coral tissue loss disease (SCTLD) has contributed to substantial declines of reef-building corals in Florida. The emergence of this disease, with its ability to impact more than 20 scleractinian coral species, has generated a need for ~~both~~ widespread reef monitoring and the implementation of novel survey and disease mitigation strategies. This study paired SCTLD prevalence assessments with colony-level monitoring to help improve understanding of disease dynamics on both individual coral colonies and reef-wide scales. Benthic surveys were conducted throughout the northern Florida Reef Tract to monitor the presence/absence of disease, disease prevalence, and coral species affected by SCTLD. Observed SCTLD prevalence was lower in ~~Palm Beach~~Jupiter and Palm Beach than in ~~Broward~~ Lauderdale-by-the-Sea or ~~Martin~~ St. Lucie Reef~~Counties~~, but there were no significant changes in prevalence over time. To assess colony-level impacts of the disease, we optimized a low-cost, rapid 3D photogrammetry technique ~~in order~~ to fate-track infected *Montastraea cavernosa* coral colonies over four time points spanning over three months. Total colony area and healthy tissue area on fate-tracked colonies decreased significantly over time, but disease lesion area did not, and was not correlated with total colony ~~size~~area. These results suggest that SCTLD affects colonies regardless of size, therefore larger colonies, when present, should be prioritized for colony-level intervention efforts to maximize ~~success~~impact. Traditional coral surveys combined with 3D photogrammetry can provide greater insights into the ~~spatial/temporal~~ spatiotemporal dynamics and impacts of coral diseases on individual colonies and coral communities than surveys or visual estimates of disease progression alone.

Introduction

Coral cover in the Tropical Western Atlantic (TWA) has declined over the last four decades [1,2], and coral diseases have been identified as one major driver of widespread coral decline throughout the region [3]. In the 1990s, white band disease dramatically reduced coral cover of *Acropora cervicornis* and *Acropora palmata* by 95% in the TWA [4]. ~~In the~~At the same time, in the Florida Keys, white pox was responsible for up to a 70% reduction in *A. palmata* cover ~~in the late 1990s~~ [5]. Additionally, i~~Likewise, i~~ncreased disease prevalence following a coral bleaching event in 2005 caused a 60% decline in coral cover throughout the U.S. Virgin Islands [6].

Coral diseases, regardless of their host specificity, have contributed to the decline of many integral reef-building scleractinian species. While white pox, white band, and acute *Montipora* white syndrome are genus-specific, other diseases have a broad, even pan-oceanic host range [6–8]. For example, white plague type II affects 17 species of scleractinian coral across multiple genera [9]. Widespread decreases in coral cover from diseases result in an ecological shift from diverse, coral-dominated communities to more homogenous, algae-dominated communities [10,11]. Such shifts have the potential to create long-term changes in fish assemblages and fishery yields [12] as well as the loss of key ecosystem services such as fisheries habitat [13], coastal wave protection [14] and nutrient cycling [15] that can persist long after a coral disease event subsides.

Since 2014, the Florida Reef Tract (FRT) has experienced an ongoing outbreak of a newly-described coral disease responsible for widespread coral mortality throughout ~~the FRT and now other regions of~~ the TWA. Stony coral tissue loss disease (SCTLD) is characterized as a highly virulent disease that affects over 20 species of scleractinian corals in the TWA [16].

SCTLD first appeared in the summer of 2014 following the dredging of Government Cut ~~by the Army Corps of Engineers~~ in Miami-Dade County [17,18]. In subsequent years, reports of SCTLD infections have increased and spread from Miami-Dade County along the Florida Reef Tract (FRT) and into the wider TWA. To date, SCTLD has spread north to the northern terminus of the FRT in Martin County and south past the Marquesas Keys in Monroe County, with additional outbreaks observed in at least twelve territories throughout the TWA [19,20].

SCTLD manifests as lesions of necrotic tissue that spread across a colony, leaving behind denuded coral skeleton (Fig 1) [16]. SCTLD is histologically distinct from white plague, exhibiting a fast-acting, liquefactive necrosis after lesions ~~developing~~ develop deep within coral tissue and progress to the colony surface [21]. Across multiple host coral species, SCTLD-affected colonies demonstrate altered microbial communities relative to their apparently healthy counterparts [22–24]. While both microbial culture-based ~~efforts~~ and sequencing studies are ongoing, no pathogen for SCTLD has been identified to date. Various intervention methods have been proposed and trialed including pro-biotic treatments, physical interventions, and various topical treatments [25]. The most popular has been a combination of physical intervention by trenching a firebreak around the infected tissue and covering the infected tissue with a topical antibiotic application [26]. Regardless of the method, the various proposed techniques require individual ~~attention~~evaluation and continued monitoring to ~~ensure~~determine success.

Fig 1. SCTLD lesions on a colony of *Montastraea* ~~*cavernosa*~~ *cavernosa*

(a) Fate-tracked, SCTLD-infected *Montastraea cavernosa* with (b) rendered 3D model, (c) characteristic disease lesion, and (d) necrotic tissue.

Traditionally, coral disease monitoring has relied on a combination of benthic survey methods and individual colony monitoring, the latter often ~~requiring~~ based on estimations of disease area, *in situ* linear measurements, ~~or two-dimensional (2D) photographic assessments~~or planar photography [27,28]. However, these techniques may incorporate a substantial observer bias or uncertainties associated with ~~converting 2D to~~ 2D estimations of 3D surfaces~~However underwater estimates and even 2D photographs can incorporate diver bias and inaccuracies~~ [29]. Under water ~~three-dimensional (3D)~~ photogrammetry and 3D modeling of coral colonies is an emerging technique that offers the potential to enhance both the accuracy and speed of data collection~~ when assessing coral disease impacts~~. ~~Photogrammetry has been used in a number of marine applications including cetacean and elasmobranch identification and morphology [34,35], fish assemblage and habitat characterization [28,36], and growth measurements in coral [32,33]. 3D imaging approaches have principally relied on aerial and close-range digital photogrammetry, 3D scanners, and soft-copy (digital) photogrammetry [37–39]. However, these methods require known 3D locations and positions from which camera positions and orientation are derived [40]. Recently, structure-from-motion (SfM) photogrammetry has emerged as a powerful new approach~~ Structure-from-motion (SfM) photogrammetry ~~that~~ derives 3D structure from a series of overlapping images, similar to established stereoscopic methods. Rather than requiring known *a priori* positions, SfM orientation is resolved based on common features extracted from overlapping images [30]. Conveniently, this can be done using a moving sensor, or from still images generated by an individual with a camera. Photogrammetry has been used in a number of coral reef applications including habitat characterizations [31–33], surface and volume measurements [34,35], and growth measurements [36,37]. SfM photogrammetry was first implemented on coral colonies by Bythell et al. [38] which used 10–20 overlapping photographs

to generate 3D models. As technology has progressed and computational power has become~~ing~~ increasingly affordable, SfM photogrammetry techniques for the purpose of assessing various coral metrics have been refined and improved upon [34,37]. Changes in image capture and model reconstruction ~~has~~have ~~brought about increases~~improved ~~in~~measurement accuracy and precision, especially in regards to more established surface area methods such as the foil-wrapping method [29,34,37,39,40]. As such, SfM photogrammetry has emerged as a powerful and commonly~~-~~used tool for coral reef researchers.~~The emergence of SfM photogrammetry coupled with the decreasing costs of related software and image-capture equipment has made SfM photogrammetry an increasingly popular tool among biologists. On coral reefs, SfM photogrammetry has been used mainly for large scale habitat complexity, fractal dimension, and rugosity characterization [31,37], but few studies in coral reef systems have utilized SfM photogrammetry at the organismal level [34].~~

In this study, we adapted a photogrammetry technique~~s~~ described in Young et al. [40] to track disease progression in fate-tracked *Montastraea cavernosa* colonies in Southeast Florida ~~in order~~ to gain insight into the colony-level dynamics of SCTLD. ~~we optimized and implemented a 3D photogrammetry technique designed for rapid data collection and analysis to understand the colony-level dynamics of SCTLD on fate-tracked *Montastraea cavernosa* colonies in Southeast Florida.~~ The study was designed to provide insight into colony- and community-level dynamics of this poorly understood disease by combining traditional surveys with colony fate-tracking using ~~3D~~ SfM photogrammetry. The ultimate goal is ~~the~~to increase widespread application of this and similar techniques ~~new method~~ to improve the design, implementation, and success of coral disease intervention, mitigation, and management strategies.

Methods

Disease prevalence surveys

Four locations across the northern Florida Reef Tract (NFRT) were selected for disease prevalence surveys: St. Lucie Reef (SLR), Jupiter (JUP), Palm Beach (PB), and Lauderdale-by-the-Sea (LBTS, Fig 2).~~, these were areas of known, vulnerable coral communities with the goal of finding sufficient SCTLD incidence for subsequent colony fate-tracking analyses.~~ ~~St. Lucie Reef is located in Martin County, Jupiter and Palm Beach are both located in Palm Beach County, and Lauderdale-by-the-Sea is located in Broward County.~~ The work conducted at SLR was done so under the St. Lucie Inlet State Park Permit 06261715, issued by St. Lucie Inlet State Park and FWC SAL-17-1960-SCRP, issued by Florida Fish and Wildlife Conservation Commission. The work conducted at JUP, PB, and LBTS was conducted under FWC SAL-17-1960-SCRP, issued by Florida Fish and Wildlife Conservation Commission. Following Hurricane Irma in September 2017, a rapid-response damage and disease survey effort was ~~completed~~ conducted throughout Southeast Florida [41]. The ~~resulting~~ data from these initial surveys were used to select the sites for the present study based on occurrence of coral communities and prevalence of SCTLD~~inform decisions of which sites within the NFRT to target for continued monitoring and fate-tracking~~ (Fig 2).

Fig 2. Map of study locations throughout the Northern Florida Reef Tract Florida
Red circles indicate roving diver survey sites and red triangles indicate sites where both roving diver surveys and coral fate-tracking occurred (Florida boundary and benthic hard bottom shapefile source: Florida Fish and Wildlife Conservation Commission-Fish and Wildlife Research Institute).

Roving diver disease surveys were conducted approximately monthly from November 2017 to June 2019. ~~-~~These surveys were designed to assess the greatest reef area possible,

quantifying disease prevalence over an estimated range of 100–2000 m^2 per survey ~~based~~ dependent on ~~, conditions, principally~~ underwater visibility. SCUBA divers swam for 20 min and recorded the species and disease status of every living coral colony ≥ 10 cm in diameter. Paling, partial bleaching, and bleaching were also noted ~~within surveys~~. ~~From those data,~~ SCTLD abundance and prevalence ~~, species diversity, and species richness~~ were calculated from these data. Statistical tests were run in the R statistical environment [42]. Non-parametric tests were implemented for all analyses as d~~D~~atasets were non-normal and normal distributions could not be achieved through transformation~~; therefore, non-parametric tests were implemented for all analyses~~ ~~unless otherwise noted~~. Permutational analysis of variance (PERMANOVA; 9999 permutations) was used in the package *vegan* [43] to assess variation in disease prevalence among sites and survey times, with Bonferroni corrected pairwise comparisons using the package *pairwiseAdonis* [44].

Fate-tracking of SCTLD-affected *M. cavernosa*

~~Fate-Tracking of SCTLD affected *M. cavernosa*~~

Benthic survey data indicated that disease incidence was too low at sites in Jupiter and Palm Beach ~~sites~~, and that coral abundance was too low at sites in St. Lucie Reef, ~~for~~ to ~~perform~~ conduct statistically robust fate-tracking ~~study~~ studies in these locations. Consequently, only ~~T~~three fate-tracking sites ~~(T328, BC1, and FTL4)~~ were established in Lauderdale-by-the-Sea (T328, BC1, and FTL4) ~~where sufficient disease incidence and coral abundance were present~~. These three sites are ~12 km from the nearby Hillsboro Inlet, less than 500 m from shore, and have been previously used for benthic and coral ~~monitoring and surveys~~monitoring [45–47] within the NFRT (Fig 2). *Montastraea cavernosa* was selected for colony fate-tracking due to

the abundance of infected colonies within the study sites. This coral species is considered intermediately susceptible to SCTLD, with onset of tissue loss occurring weeks to months later than what has been reported for highly susceptible species (e.g. *Dendrogyra cylindrus, Meandrina meandrites, Colpophyllia natans*). Lesions on infected *M. cavernosa* generally progress slower ~~than in~~as compared to highly susceptible coral species, with total mortality occurring within months to years [16]. *Montastraea cavernosa* constituted ~~approximately~~ 1~~0~~1.6% of the reported cases of SCTLD in the NFRT ~~during post-Hurricane Irma disease and breakage assessments~~in late 2017 [41].

Twenty-four colonies of *M. cavernosa* affected with SCTLD were tagged with ~~individually~~ uniquely numbered cattle tags across the three sites on 24-August-2018 (T_1). Sites were revisited on ~~(T328, BC1, FTL4; Fig 2). Sites were visited four times in 2018: 24-Aug-2018 (T_1),~~ 11-Sep-2018 (T_2), 8-Nov-2018 (T_3), and 17-Dec-2018 (T_4)~~)~~. ~~*In situ* observations made for each colony included the presence/absence of disease, the number of disease lesions, and diver-based *in situ* estimates of percent mortality. Photographs were taken of each colony along with c~~Continuous video was taken for 3D model generation to ~~quantitatively measure~~quantify total colony surface area and disease lesion area for each time point.

Fate-tracked colonies were filmed using methods outlined in Young et al. [40], with the following modifications: Canon G16 cameras in Fantasea underwater housings were set on “Underwater mode,” 1080p and 60 frames per second (fps), and exposure was adjusted as needed based on ambient light conditions. One-meter, L-shaped PVC scale bars marked at 10 cm increments were placed at opposing right angles to frame the designated colony. A SCUBA diver maintained approximately 1 m ~~altitude~~ distance above the highest point of each coral colony and recorded continuous video while swimming repeated ~~linear, parallel, adjacent~~ passes in a

lawnmower pattern with the camera pointed directly downward. The number of adjacent passes varied depending on colony size, ~~with~~ allowing for 60–70% overlap ~~between passes to aid in downstream model generation~~. The camera was rotated 90° at the end of the first set of adjacent passes~~,~~ ~~then~~and ~~then~~ another set of passes was completed perpendicular to the first set. The two complete sets of passes for a single colony required between 1–3 min of dive time, depending on colony size. Each sampling event produced an average of 14 GB of .mp4 video files, or approximately 425 MB of video file size per coral colony.

Video processing and 3D model generation protocols are described in full in our GitHub repository (https://github.com/icombs2017/analysisOf3dModels/tree/master/3D%20Modeling/protocols~~https://github.com/icombs2017/analysisOf3dModels~~). In summary, the free software package, *FFmpeg* (www.ffmpeg.org), was used to extract still frame images from videos of the fate-tracked colonies at a rate of 3 fps. Still images were then imported into AgiSoft Metashape Standard Edition ~~-~~(Version 1.5.2, AgiSoft LLC) software, which uses a proprietary algorithm that incorporates SfM and Brown's lens distortion model to generate 3D models from 2D images [30,48]. Model generation in Metashape was conducted according to the manufacturer's protocol in four general steps: 1) camera alignment, 2) dense point cloud generation, 3) mesh generation, and 4) texture overlay. Models were rendered on a 2018 Apple MacBook Pro with a 2.9 GHz processor, 16GB of RAM and a Radeon Pro Vega 16 4GB graphics card. A single model took approximately 40 min to render depending on the number of still images generated. Generated models were then exported as an~~n~~ .obj file and imported into the software Rhinoceros 3D (Robert McNeel & Associates) for analysis; the mean model file size was 64 MB.

Models were scaled using the PVC scale bars, then total colony surface area and disease lesion surface area were measured~~ ~~ by hand tracing polygons around coral tissue~~with Rhinoceros 3D [37], where total colony area included both healthy and diseased coral tissue~~. SCTLD disease lesion area was ~~considered~~ defined as the stark white newly-dead coral tissue or~~ very~~ ~~newly-dead~~ skeleton with sloughing tissue that is ~~a~~ characteristic of ~~this~~the disease margin.~~,~~ B~~and b~~oth total colony area and disease lesion area were generated directly within Rhinoceros 3D, while healthy tissue area was calculated by subtracting disease lesion area from total colony area. Proportion of loss and ~~R~~rate of tissue loss per week ~~and change in disease tissue area per week~~ were calculated for each pair of time points. ~~Additionally, lesion count was derived from the number of polygons created when tracing the disease lesion area.~~ To identify significant effects of time on healthy area, diseased area, total area, the proportion of tissue loss and the rate of tissue loss~~, and change in disease tissue area~~, Friedman's rank sum tests were conducted using the package *PMCMRplus* [49]. Pairwise comparisons were made with Nemenyi tests in the package *PMCMR* [50]. Additionally, Spearman's rank correlation analyses were used to ~~compare~~ correlate diseased area and total colony area~~, lesion count and disease area, lesion count and total colony area,~~ and rate of tissue loss and total colony area.

3D model validation

Accuracy of model-generated surface area metrics was assessed by a validation experiment using a standardized mock coral colony. A square cupola prism with equal dimensions and surface features was constructed to represent a coral colony using ½" PVC and internal steel rebar to achieve negative buoyancy. The prism was constructed using a top square of 25.4 x 25.4 cm, a bottom square of 47.6 x 47.6 cm, and height of 30.5 cm (S1 Fig). Angled

sides were achieved using 45° PVC tees to better represent a mounding coral colony. Additionally, polygons of known surface areas (square: 40.32 cm^2; rectangle: 12.90 cm^2; circle: 45.60 cm^2) were designed using graphics editing software, printed to scale on waterproof paper, and affixed to the top and sloped sides of the weighted prism.

The prism was ~~3D modeled~~filmed in a pool and at ~~two reef sites~~the three coral fate-tracking sites in Lauderdale-by-the-Sea ~~described in the previous section~~ ~~(St. Lucie Reef and Lauderdale-by-the-Sea;~~(Fig 2) to ~~emulate variable water quality and benthic community parameters that may influence model generation~~quantify model error in pristine (pool) versus reef conditions (e.g. variable~~,~~ turbidity, currents, surge). ~~Nine~~ Eight ~~replicate~~ videos were ~~captured~~ recorded for~~of~~ the prism and boundary frames ~~in each of~~across the three reef ~~three locations~~sites (2–3 replicates per site), while six prism videos were captured in the pool ~~pool for comparability between pool and reef locations~~. The prism was filmed at a ~~pool~~ depth ~~was~~ of 2.75 m in the pool, 8.84 m at BC1, 5.18 m at T328, and 6.71 m at FTL4~~; , while~~ filming distance remained the same for all videos. ~~among sites and reef locations ranged from 3.96–10.05 m at St. Lucie Reef, and 5.18–8.84 m at Lauderdale-by-the-Sea.~~ To assess accuracy of surface area measurements, ~~Site locations were selected based on proximity to existing monitoring and fate-tracking efforts and represented two different bottom types characteristic of reef habitats in the NFRT (wormrock at St. Lucie Reef, and reef carbonate at Lauderdale-by-the-Sea). Additionally, benthic communities varied between the two reef locations from sparsely-populated scleractinian and turf algae communities, to gorgonian and scleractinian dominated communities, respectively.~~ 3D models were generated and analyzed using the same protocols described earlier, with polygons traced for each of the prism shapes based on the four corners for squares and rectangles, and the entire circumference for circles. Replicate surface area measurements of

shapes were made for each model according to face of the prism (top and four sides), and number of shapes on each face (four for square and rectangle, one for circle), for a total $n = 229$ measurements for square, $n = 232$ for rectangle, and $n = 59$ for circle. To assess vShape errorariation in shape surface area measurements both within and among models was, shape error was calculated as the absolute difference between measured shape surface areas and the corresponding template area. Differences in overall model areas among locations were assessed using a single-factor ANOVA. Surface area and eShape error measurements were found to be highly right-skewed, so therefore a square-root transformation was applied prior to statistical analyses. Non-parametric Kruskal-Wallis tests were run for each prism template shape (square, rectangle, circle) to determine whether surface area measurements differed by location. Pairwise comparisons were made using Dunn's tests for each of the prism shapes using the R package *FSA* [52]. Multivariate homogeneity of variance was first tested using the *betadisper* function of the package *vegan*, then a three-factor nestedsingle-factor PERMANOVA was conducted across location, model, and prism facesites using the *adonis* function of the package *vegan*. Pairwise PERMANOVA tested for significant differences among pairwise comparisons of each factor using the package *pairwiseAdonis*. To assess variation in shape surface area measurements both within and among models, shape error was calculated as the absolute difference between measured shape surface areas and the corresponding template area. The error dataset was then analyzed using the same statistical tests as described above, except that a subset dataset was created which did not include the template values. Lastly, correlation analyses were conducted to determine if overall model area was significantly related to each of the three shape's error measurements for all models. Correlation analyses utilized Spearman's rank correlation coefficients and were conducted for each of the three locations within each prism shape.

Additionally, a subset of four models from the colony fate-tracking dataset were extracted at three different frame rates (3–5 fps) across their respective time points to determine if frame rate affected model accuracy. A ~~non-parametric~~ Kruskal-Wallis test was used to determine if model size differed across the different extraction frequencies.

Results

Disease prevalence surveys

SCTLD prevalence varied significantly across ~~county~~ location (~~PERMANOVA; $F_{2, 42}$ = 12.24, p < 0.001,~~ Table 1), but not through time~~,~~ (Table 1, Fig 3). Pairwise comparisons indicated that disease prevalence was lower in JUP and PB than in SLR and LBTS (~~Palm Beach County was lower than both Martin and Broward counties (pairwise PERMANOVA; Martin: F = 14.87, p < 0.002, Broward: F = 53.61, p < 0.001;~~ Table 1, Fig 3). In LBTS, disease prevalence stayed ~~quite~~relatively constant (10.9%) while in JUP and PB prevalence was consistently low, but peaked in March 2019 with SCTLD prevalence at 8.1% and 4.3%, respectively.~~Relatively lower levels of disease prevalence were observed in Palm Beach County from December 2017 – December 2018, with increasing disease prevalence at nearly all sites in March 2019.~~ Prevalence d~~D~~ata from SLR ~~wa~~was highly varied due to generally low coral cover observed during the survey period. ~~Disease monitoring did not begin in Broward County until November 2018; however, an increase in disease prevalence was also observed in March 2019 (Fig 3).~~ ~~Additionally~~For example, the highest reported disease prevalence of 43% at site SLR North ~~in Martin County~~ was ~~affected by low sample siz~~due to low coral cover; only 7 living corals were observed, 3 of which were diseased. The most abundant species throughout our surveys were *M~~ontastrae.~~a cavernosa* (63%, *n* = 3241), *Porites astreoides* (17%, *n* = 885), *Siderastrea siderea*

Formatted: Font: Italic

Formatted: Font: Italic

(4%, *n* = 199), *Stephanocoenia intersepta* (2.3%, *n* = 118), and *Agaricia agaricites* (2.1%, *n* = 108). Species abundance varied among the four locations (Kruskal-Wallis, *H* = 10.308, *p* = 0.016) with species abundance varying at SLR relative to the other three locations (Dunn's Test, all *p* < 0.05). All locations had *M.~~ontastraea~~ cavernosa* as the most abundant species, except for SLR which had *P~~orites~~. astreoides* as the most abundant species (65%, *n* = 304). The species with the highest disease prevalence with more than 10 observations were *Pseudodiploria clivosa* (36%, *n* = 47), ~~*Agaricia*~~ *A. agaricites* (10%, *n* = 108), *Dichocoenia stokesii* (9%, *n* =23), *Orbicella faveolata* (6%, *n* = 49) and ~~*Montastraea*~~ *M. cavernosa* (5%, *n* = 3241).

Table 1. Results from univariate-permutational analysis of variance (PERMANOVA) comparing SCTLD prevalence from the roving diver surveys across location and time. Pairwise comparisons from results of the univariate PERMANOVA across all ~~three~~ four ~~counties~~ locations: ~~(Martin~~St. Lucie Reef (SLR), Jupiter (JUP), Palm Beach (PB), and ~~Broward County~~Lauderdale-by-the-Sea (LBTS)~~)~~.

Test	Comparison	df	Psuedo-*F*	*p*-value*
PERMANOVA	Location	3	7.44	< 0.001
	Date	12	1.23	ns
	Location:Date	7	0.52	ns
Pairwise	LBTS – SLR		0.11	ns
	LBTS – JUP		26.76	< 0.001
	LBTS – PB		35.91	< 0.001
	SLR – JUP		7.12	0.0492
	SLR – PB		7.88	0.045
	JUP – PB		0.63	ns

*Non-significant *p* values listed as "ns".

Formatted: Font: Italic
Formatted: Font: Italic
Formatted: Font: Italic
Formatted: Font: Italic
Formatted: Font: Italic
Formatted: Font: Italic

Fig 3. Mean SCTLD prevalence across all ~~three~~ four locations ~~counties~~ (~~Broward~~St. Lucie Reef, ~~Martin~~Jupiter, ~~and~~ Palm Beach ~~Counties~~and Lauderdale-by-the-Sea). Points represent means; bars represent standard error.

Fate-tracking of SCTLD-affected *M. cavernosa*

~~Fate-Tracking of SCTLD affected *M. cavernosa*~~

Friedman's rank sum tests indicated a significant decrease in total colony area (m^2) and healthy tissue area over time (~~total: $df_{3,94}$, Friedman $\chi^2 = 48.56$, $p < 0.001$; healthy: $df_{3,94}$, Friedman $\chi^2 = 41.29$, $p < 0.001$;~~ Table 2, Fig 4). Disease lesion area significantly varied through time, however pairwise comparisons revealed that one time point comparison (T_2—T_4) was driving the variation (Table 2). ~~The number of disease lesions per colony differed by site (Kruskal-Wallis, H = 17.08 p < 0.001, S1 Table), with T328 exhibiting more disease lesions per colony (9.57 ± 1.07) than both BC1 (4.31 ± 0.51) and FTL4 (4.65 ± 0.87; Dunn's test, both $p < 0.05$; S1 Table). Correlation analyses between surface area and lesion metrics identified no significant correlation between colony size and disease lesion area (Spearman's rank correlation $r_s = 0.15$, $p = 0.154$, Fig a in S3 Fig). There was, however, a positive correlation between colony lesion number and disease lesion area (Spearman's rank correlation, $r = 0.67$, $p < 0.001$, Fig c in S2 Fig), but no correlation between total colony size and the number of disease lesions (Spearman's rank correlation, $r_s = 0.18$, $p = 0.076$, Fig b in S2 Fig).~~

Table 2. Results of Friedman's rank sum tests comparing total colony area, disease tissue area and healthy tissue area across time, with pairwise comparisons made using Nemenyi tests.

**Test**	**Comparison**	**Test Statistic**	***p* value***
Total Colony ~~Size~~Area	Friedman’s	48.55	< 0.001
	$T_1 – T_2$	4.27	ns
	$T_1 – T_3$	7.59	< 0.001
	$T_1 – T_4$	9.01	< 0.001
	$T_2 – T_3$	3.32	ns
	$T_2 – T_4$	4.74	0.026
	$T_3 – T_4$	1.42	ns
Healthy Tissue Area	Friedman’s	41.29	< 0.001
	$T_1 – T_2$	4.27	0.026
	$T_1 – T_3$	7.59	< 0.001
	$T_1 – T_4$	9.01	< 0.001
	$T_2 – T_3$	3.32	ns
	$T_2 – T_4$	4.74	ns
	$T_3 – T_4$	1.42	ns
Disease Lesion Area	Friedman’s	14.02	0.003
	$T_1 – T_2$	4.27	ns
	$T_1 – T_3$	7.59	ns
	$T_1 – T_4$	9.01	ns
	$T_2 – T_3$	3.32	ns
	$T_2 – T_4$	4.74	0.012
	$T_3 – T_4$	1.42	ns

T_1, 24-Aug-2018; T_2, 11-Sep-2018; T_3, 8-Nov-2018; T_4, 17-Dec-2018.

*Non-significant *p*- values listed as “ns”.

Fig 4. Comparison of tissue areas through time. Mean tissue areas through time (a) mean total colony area (cm^2) (b) mean healthy tissue area (cm^2), and (c) mean disease lesion area ~~each~~ across all sites and through time for fate-tracked *M. cavernosa* colonies (*n* = 24). T_1, 24-Aug-2018; T_2, 11-Sep-2018; T_3, 8-Nov-2018; T_4, 17-Dec-2018.

A Friedman's rank sum test revealed significant differences between rates of tissue loss through time ($df_{2,71}$, Friedman $\chi^2 = 6.25$, $p = 0.044$). The rate of change in disease area did not significantly differ over time (Friedman's rank sum test, $df_{2,71}$, Friedman $\chi^2 = 2.58$, $p = 0.275$).

Average tissue loss between T_1–T_2 was -98.17 ± 40.74 cm^2 wk^{-1}, -24.99 ± 7.70 cm^2 wk^{-1} between T_2–T_3, and -45.10 ± 19.64 cm^2 wk^{-1} between T_3–T_4 (Fig S54a). A Friedman's rank sum test revealed significant differences between rates of tissue loss through time ($df_{2,71}$, Friedman $\chi^2 = 6.25$, $p = 0.044$). Rate of tissue loss was not significantly different among sites between any timepoint (S12 Table). On average, the proportion of tissue lost was 37.1 ± 7.2% over the course of this study, with three colonies reachingexperiencing completefull mortality. Correlation analyses identifiedThere was no significant correlation between total colony area and disease lesion area (Spearman's rank correlation $r_s = 0.15$, $p = 0.154$, Fig 6), or between rates of tissue loss and total colony area (Spearman's rank correlation $r_s = -0.046$, $p = 0.704$, Fig 5b)), and the rate of tissue loss was never correlated with colony size between any timepoint (S3 Table, Fig b in S4 Fig).

Formatted: Line spacing: Double

Fig 5. Comparison of rates of tissue loss. (a) Mean change in tissue area on fate-tracked *M. cavernosa* colonies and (b) Spearman's rank correlation between rate of tissue loss ($cm^2 wk^{-1}$) and total colony area (cm^2).

Formatted: Font: Not Bold

Formatted: Font: Not Bold, Superscript

Formatted: Font: Not Bold

Fig 6. Correlations between total colony area and disease lesion area. **S2 Fig. Plot of mean change in tissue area on fate-tracked *M. cavernosa* colonies from pairwise time comparisons (T_1–T_2, T_2–T_3, T_3–T_4).** Spearman's rank correlation between disease lesion area (cm^2) and total colony area (cm^2) for fate-tracked *M. cavernosa* colonies ($n = 24$).

Formatted: Line spacing: Double

Formatted: Font: Italic

Formatted: Font: Bold

3D model validation

~~3D model accuracy assessment~~

~~Overall model area differed significantly among locations, with Lauderdale-by-the-Sea and St. Lucie Reef models being larger than those generated in the pool ANOVA: $F_{2,18}$ = 18.21, p < 0.001). Kruskal-Wallis tests determined that only rectangle areas varied among locations (S4 Table; S4 Fig; p < 0.001), which was driven by pairwise differences between pool models and both Lauderdale-by-the-Sea and St. Lucie Reef locations (all p < 0.001).~~ Assessment of multivariate variance identified heterogeneous variance in shape surface area error among ~~locations~~sites~~, with the highest variance attributed to the location with the lowest sample size (St. Lucie Reef)~~ due primarily to lower variance at site FTL4. ~~The~~ A ~~three-factor~~ PERMANOVA ~~identified~~ determined that shape error ~~surface area~~ did not vary ~~across~~ across the pool and three coral fate-tracking ~~locations or face, but that model was a significant factor explaining 27.71% of the observed variance~~ sites in Lauderdale-by-the-Sea (~~S5 Table;~~ $F_{316,279309}$ = ~~8.09~~0.733, p = 0.~~043~~584). The mean shape error across all sites was 1.70 ± 0.09 cm^2 (pool: 1.37 ± 0.10 cm^2; BC1: 1.97 ± 0.23 cm^2; T328: 2.38 ± 0.30 cm^2; FTL4: 1.82 ± 0.18 cm^2), which corresponded to an error rate of 6.13 ± 0.27% (pool: 4.87 ± 0.32%; BC1: 7.02 ± 0.67%; T328: 8.72 ± 0.92%; FTL4: 6.87 ± 0.57%).~~Pairwise PERMANOVA results for shape area can be found in S5 Table.~~

~~Statistical analyses of shape errors, on the other hand, showed that location was a significant factor for all three shapes (S4 Table; S4 Fig; square: p < 0.001, rectangle: p < 0.001, circle: p = 0.0063). Results of the pairwise comparisons among locations can be found in S4 Table. Multivariate variance was heterogeneous among locations as with the shape area dataset.~~

PERMANOVA found all main model and interaction terms to be significant factors (S5 Table; PERMANOVA; location: $F_{16,309} = 29.59$, $p < 0.001$; model: $F_{16,309} = 8.42$, $p < 0.001$; face: $F_{16,309} = 4.01$, $p = 0.0067$), with main terms explaining 39.08% of the model variance. Pairwise PERMANOVA results for shape error can be found in S5 Table. Since there was a significant difference in overall model area among locations, nonparametric correlation analyses were run to determine whether shape error increased with increasing model area. For each of the three prism shapes, only St. Lucie Reef models showed significant correlations (S4 Fig; square: $r_s = 0.53$, $p < 0.001$; rectangle: $r_s = 0.58$, $p < 0.001$; circle: $r_s = 0.64$, $p = 0.0056$), where shape error was higher with larger models.

To assess the potential effects of different frame rates used during model generation on colony-scale surface area measurements, a single-factor Kruskal-Wallis test indicated that frame rate had no significant impact on total colony area (Kruskal-Wallis, H = 0.12, $p = 0.94$). This finding confirmedsuggests that higher frame rates can be used from the initial video can be used to rectify improve poorly constructed models without affecting downstream spatial analyses. To keep processing time as low as possible, however, stills were extracted at 3fps unless otherwise necessary.

Discussion

Disease prevalence surveys

SCTLD is a unique coral disease due to itsthe spatial geographic extent, the number of coral species affected, and rapid rates of disease progression and spread [16,17,51,52]. It has spreadSCTLD has been reported across over ~400 km alongof the FRT, and now has been

Formatted: English (United States)

Formatted: English (United States)

observed in at least 12 other territories throughout the TWA [20].~~;~~ Other coral diseases, such as white syndrome, have been observed over broader spatial extents (~1500 km) in the Great Barrier Reef [53]. Likewise, black band disease has been observed on coral reefs across multiple oceans [54]. ~~,~~ T~~t~~he ~~full~~ geographic extent of SCTLD ~~is likely not fully realized~~continues to expand with~~as~~ new observations ~~are being reported~~ throughout the Caribbean [19,20]. SCTLD has been reported as far west as Belize and as far southeast as Martinique which are 1300 and 2500 km respectively from the northern terminus of the NFRT at St. Lucie Reef, FL [20]. The ~~global~~overall mean disease prevalence for the NFRT observed in this study was 6%, which is lower than recorded in ~~previous surveys after~~post Hurricane Irma surveys in 2017 [41] and ~~in~~ previous SCTLD surveys in SE Florida~~efforts~~ [51]. Sites in Jupiter and Palm Beach had relatively low disease prevalence (1.7% and 1.0%) ~~which is~~ consistent with background disease levels (1–3%) within the TWA [55]. The highest values observed in this study were between 21~~% -~~–43% at SLR, but no sites reached ~~some of~~ the highest reported disease prevalence values of 60% observed near Miami~~as reported by Precht et al.~~ [17]. The lower prevalence values reported in this study ~~versus the latter study~~ may be ~~in part~~ due in part to differences in species composition between the NFRT and southern regions of the FRT. The most ~~highly~~ abundant and susceptible species ~~in previous studies~~ such as *M~~eandrina~~. meandrites*, *D.~~ichocoenia~~ stokesi*, and *Pseudodiploria strigosa* [17,51] were comparatively absent within our NFRT surveys.~~-~~ However, ~~likely due to relative rarity and~~ previous SCTLD impacts before our monitoring sites were established may also have affected the relative abundance of susceptible species [51,56].

~~SCTLD is a unique coral disease due to its extent, number of species affected, and rapid progression [16,17,49,50]. It has spread over 400 km along the FRT, and now has been observed in at least 12 other territories throughout the TWA [20]. In contrast, one of the most widespread~~

outbreaks of acute *Monitpora* white syndrome to affect the Hawaiian archipelago only spanned ~7.5 km throughout Kāne'ohe Bay [51]. Other diseases, such as the species-generalist white syndrome, have been observed over broader spatial extents (~1500 km) in the Great Barrier Reef [52], yet the full extent of SCTLD is likely not fully realized as new observations are being reported throughout the Caribbean [19,20]. It is also important to note that throughout the NFRT, SCTLD prevalence among the four monitoring sites was higher than typically observed background disease prevalence values (~1–3%) for the TWA [53]. Many sites had no observed disease at discrete time points, but disease prevalence observed in this study was as high as 45% at some sites. This is in conjunction with previously-reported prevalence values of more than 60% disease prevalence among other sites throughout the FRT [17], suggesting that SCTLD can be uniquely well-distributed yet locally infectious compared to other coral diseases. The lower prevalence values reported in this versus the latter study may be in part due to differences in species composition between the NFRT and southern regions of the FRT. The most highly abundant and susceptible species in previous studies [17,49] were comparatively absent within our NFRT surveys likely due to relative rarity and previous SCTLD impacts before our monitoring sites were established [49,54].

Disease prevalence significantly varied across site, which is consistent with survey data generated from post-Hurricane Irma disease assessments [39] and likely due to highly variable coral abundances across the monitoring locations. Sites with the highest disease prevalence in Martin County were characterized by relatively low overall coral abundance and species richness as compared to sites in Palm Beach and Broward Counties to the south. The global mean disease prevalence for the NFRT observed in this study was 6%, which is similar to prevalence after Hurricane Irma (Walker 2018) and in previous SCTLD survey efforts [49].

We observed an increase in disease prevalence during the spring of 2018 which was unexpected, as prevalence for other described coral diseases often increases during the summer months as water temperatures increase [57–60]. SCTLD prevalence does not appear to have a positive correlation with temperature [17,51] as has been observed for other coral diseases [58,61,62], but potential environmental cofactors that may drive SCTLD prevalence need to be examined further.

Formatted: English (United States)

Fate-t~~T~~racking of SCTLD~~-~~ a~~A~~ffected ~~-~~*M. cavernosa*

At the colony level, disease progression was highly variable across fate-tracked *M. cavernosa* colonies near Lauderdale-by-the-Sea. As expected, total colony ~~size~~ area and healthy tissue area decreased significantly over time, demonstrating the impact SCTLD can have on living coral cover over just a few months. Over the course of ~~this study~~fate-tracking period (115 days), colonies lost ~~o~~an average ~~of~~ 37.1 ± 7.24% of their tissue surface area. In contrast, Aeby et al. [24] noted subacute tissue loss in *M. cavernosa* was 34 ± 8.7% over 1 year~~, however,~~ using a semi-quantitative method ~~was used to calculate~~to estimate tissue~~ loss as they found the morphological complexity of the colonies did not allow for a photographic assessment~~. Additionally, using a similar 3D photogrammetry method, Meiling et al. [63] saw proportional losses of 2.0 ± 0.11% day^{-1} of six *M. cavernosa* colonies in the US Virgin Islands. ~~DIn their study, hHowever, din the study by Meiling et al. [63] due tothat occurred during their the observation period. observation period.~~

Disease lesion area did not correlate with total colony ~~size~~ area, suggesting that larger colonies do not exhibit a higher proportion of diseased tissue ~~as~~ compared to smaller colonies. As SCTLD progresses on coral colonies, loss of healthy tissue appears to be a more important

Formatted: Indent: First line: 0.5"

indicator of disease virulence, as opposed to change in disease lesion area. Importantly, ~~however,~~ rates of tissue loss did not correlate with total colony ~~size~~area. Therefore, larger corals may have a more favorable time horizon for potential intervention actions that could prevent total colony mortality. Conversely, smaller infected colonies may succumb more quickly to SCTLD. Considering the level of effort~~-~~ required for SCTLD interventions [64], prioritizing interventions on larger colonies with sufficient areas of uninfected tissue remaining may result in more successful disease mitigation~~ a great preservation of overall coral cover.~~. ~~Moreover, since smaller infected colonies are more likely to succumb to SCTLD, it may be appropriate to cull affected colonies, removing them from the environment to prevent further SCTLD transmission. Targeting relatively larger coral colony size classes and conducting intervention during early stages of infection when possible may both improve the likelihood of successfully preserving coral cover.~~

3D model generation as a fate-tracking method

This study tested a relatively inexpensive and ~~accurate~~ rapid 3D model generation ~~pipeline~~ methodology described in Young et al. [40] ~~(2017)~~ as a means to ~~fate-track individual coral colonies~~track disease progression in entire coral colonies -without substantial increases in expense, time, or computational requirements compared to observational and photographic techniques. ~~Using simple computational methods, s~~Surface areas of complex coral morphologies were calculated to generate ~~more accurate~~ data ~~than previously established methods, such as two-dimensional surface area estimation using photo processing software [66] or *in situ* linear extension measurements [25,67,68]~~on live versus dead coral tissue over time. Additionally, this 3D modeling technique was validated through the quantification of model error using a mock

coral colony deployed in various underwater environmentswith standardized surface shapes. While Sshape area error (i.e. variation between measured surface areas and template shapes) did not vary across locations or position on the prism, there was significant variation among iterations of models within locations (and with respect to shape error), emphasizing the need for sufficient replication for both statistical robustness and model troubleshootinga pool and the three coral fate-tracking sites in Lauderdale-by-the-Sea (S42 Fig). There was 2.17 ± 0.11 cm^2 of error in measurements of shapes with known surface area affixed to the sloping sides and top of the prism, which is relatively minor at a colony-level scale as the measured error represents <0.1% colony size on average.Global shape error across all sites was 1.70 ± 0.09 cm^2, which corresponded to an error rate of 6.13 -± 0.27%. This is likely an inflated estimate of model error, as the prism shapes used for accuracy assessments were printed on waterproof paper that could move with currents, rather than fixed in place as are scleractinian corals.

In this study colony size did not affect model accuracy, however, overall model size may be affected if water quality parameters or oceanographic conditions are a limiting factor to 3D model quality (i.e. in nearshore reef environments; S2 Fig). There were some instances, however, where model generation was poor due to anomalies in the Metashape algorithm, inconsistent underwater filming, or most common, reduced water qualityhigh turbidity, all of which may impact surface area measurements and therefore model error. Normally Typically, this these issues could be rectified by extracting still images from the video at an increased frame rate to ensure higher overlap among images, or by collecting replicate videos for each target. Notably, extracting at a higher fps improved poor models, but did not disproportionately affect accuracy of modelssurface area measurements from models that were sufficiently capturedconstructed well using images extracted at 3 three frames per minute fps.

Due to Metashape’s proprietary algorithm, further adjustments within the model generation process to rectify poor models are limited. Stable, high-resolution image collection is therefore integral to successful downstream model generation. Alternatives to the lawn-mower video path used in this study may be more effective but also more time-intensive, particularly for tall (>1 m), highly-rugose coral colonies. Discrete, overlapping photographs could be taken instead of continuous video [36], or video could be taken while swimming a circular pattern around the coral at varying angles [65]. ~~While the technique presented here was optimized for individual coral colonies, stereoscopic camera setups, with one wide lens and one zoom lens, may be more appropriate for reef-scale photogrammetry [66].~~ Critically, while disease monitoring via linear extension measurements may be able determine potential differences among colonies or treatments over time [16,27,66], linear extension does not accurately quantify tissue loss and may underestimate the impacts of disease lesions on coral colonies. Therefore, 3D approaches, such as the method presented here, should be employed to ~~accurately~~ quantify lost coral surface area and to identify colonies for which intervention efforts may be successful.

Formatted: Line spacing: Double

Conclusions

SCTLD is particularly non-discriminant compared to other coral diseases ~~with regard~~relative to its host specificity and seasonal distribution. Given this study’s ~~observations~~ comparisons ~~relative to~~between tissue loss and coral colony sizes, intervention efforts~~s~~ on smaller colonies with numerous active lesions may be less ~~in~~efficient ~~and ultimately~~

unsuccessful. Instead, In instances where time, personnel, weather opportunities, or boating and diving resources are limitedif, the results of this study suggest that- larger, longer-lived colonies should be prioritized when are present at a targeted monitoring site, attemptings to mitigate limit the impacts of SCTLD. at the colony level should consider 1) culling small colonies to prevent disease transmission and/or 2) shifting intervention focus toward the larger, longer-lived colonies with more tissue remaining. Targeted and prioritized interventions may be particularly important in instances where time, personnel, weather opportunities, or boating and diving resources are limited. Similarly, sSince SCTLD is a progressive, necrotic infection, the area of tissue loss, or proportion of tissue loss, may represent an alternative, more impactful metrics forto quantifying the severity of infection and disease progression, and therefore may be more informative thanas opposed to disease lesion area or percent of affected tissue. However, aAdditional studies incorporating longer timescales, multiple species, and similar quantitative 3D metrics are warranted to confirm these patterns of disease progression across all susceptible species. Rapid, cost-effective, and accurate methods using 3D models for quantifying coral surface area are quite feasible so long as the appropriate redundancies and precautions are followed. It is recommended that managers and intervention specialists—particularly those focusing on SCTLD—adopt photogrammetric methods to ensure that data is are more accurate and comparable across future studies.

Data aAvailability

Model generation protocol, analysis scripts, and documentation -are available on GitHub (https://github.com/icombs2017/analysisOf3dModels). Disease prevalence observations can be found in the S1 Dataset. Surface area measurements from the fate-tracked coral colonies can be

found in the S2 Dataset. Surface area and error measurements for the three prism shapes can be found in S3 Dataset.

Acknowledgements

We thank J. Nelson and M. Roy for assistance with boating and diving operations; J. Beal, E. Shilling, and A. Sturm for assistance with data collection. K. Kerrigan, J. Walczak, B. Walker, and K. Neely provided valuable feedback on the study design.

References

1. Gardner TA, Côté IM, Gill JA, Grant A, Watkinson AR. Long-term region-wide declines in Caribbean corals. Science (80-). 2003;301: 958–960. doi:10.1126/science.1086050
2. Mumby PJ. The Impact of Exploiting Grazers (Scaridae) on the Dynamics of Caribbean Coral Reefs Author (s): Peter J . Mumby Published by : Wiley Stable URL : http://www.jstor.org/stable/40061693 Accessed : 05-04-2016 08 : 06 UTC Your use of the JSTOR archive ind. 2016;16: 747–769.
3. Richardson LL. Coral diseases: What is really known? Trends Ecol Evol. 1998;13: 438–443. doi:10.1016/S0169-5347(98)01460-8
4. Aronson RB, Precht WF. White-band disease and the changing face of Caribbean coral reefs. Hydrobiologia. 2001;460: 25–38. doi:10.1023/A:1013103928980
5. Patterson KL, Porter JW, Ritchie KB, Polson SW, Mueller E, Peters EC, et al. The etiology of white pox, a lethal disease of the Caribbean elkhorn coral, Acropora palmata. Proc Natl Acad Sci U S A. 2002;99: 8725–8730. doi:10.1073/pnas.092260099
6. Miller J, Muller E, Rogers C, Waara R, Atkinson A, Whelan KRT, et al. Coral disease following massive bleaching in 2005 causes 60% decline in coral cover on reefs in the US Virgin Islands. Coral Reefs. 2009;28: 925–937. doi:10.1007/s00338-009-0531-7
7. Nugues MM. Impact of a coral disease outbreak on coral communities in St Lucia. 2002;229: 61–71.
8. Miller MW, Williams DE. Coral disease outbreak at Navassa, a remote Caribbean island. Coral Reefs. 2007;26: 97–101. doi:10.1007/s00338-006-0165-y
9. Richardson LL, Goldberg WM, Carlton RG, Halas JC. Coral disease outbreak in the Florida Keys: Plague Type II. Rev Biol Trop. 1998;46: 187–198. Available:

Formatted: French (France)

Field Code Changed

http://www.amlc-carib.org/meetings/procs/1997AMLC_Proceedings/richardson.pdf

10. McClanahan TR, Muthiga NA. An ecological shift in a remote coral atoll of Belize over 25 years. Environ Conserv. 1998;25: 122–130. doi:10.1017/S0376892998000174

11. McManus JW, Polsenberg JF. Coral-algal phase shifts on coral reefs: Ecological and environmental aspects. Prog Oceanogr. 2004;60: 263–279. doi:10.1016/j.pocean.2004.02.014

12. Graham NAJ, Wilson SK, Jennings S, Polunin NVC, Robinson J, Bijoux JP, et al. Lag effects in the impacts of mass coral bleaching on coral reef fish, fisheries, and ecosystems. Conserv Biol. 2007;21: 1291–1300. doi:10.1111/j.1523-1739.2007.00754.x

13. Smith S V. Coral-reef area and the contributions of reefs to processes and resources of the world's oceans. Nature. 1978;273: 225–226. doi:10.1038/273225a0

14. Sheppard C, Dixon DJ, Gourlay M, Sheppard A, Payet R. Coral mortality increases wave energy reaching shores protected by reef flats: Examples from the Seychelles. Estuar Coast Shelf Sci. 2005;64: 223–234. doi:10.1016/j.ecss.2005.02.016

15. Yamamuro M, Kayanne H, Minagawao M. Carbon and nitrogen stable isotopes of primary producers in coral reef ecosystems. Limnol Oceanogr. 1995;40: 617–621. doi:10.4319/lo.1995.40.3.0617

16. NOAA. Stony Coral Tissue Loss Disease Case Definition. Florida Keys Natl Mar Sanctuary. Silver Spring, MD; 2018. Available: https://nmsfloridakeys.blob.core.windows.net/floridakeys-prod/media/docs/20181002-stony-coral-tissue-loss-disease-case-definition.pdf

17. Precht WF, Gintert BE, Robbart ML, Fura R, Van Woesik R. Unprecedented Disease-Related Coral Mortality in Southeastern Florida. Sci Rep. 2016;6: 31374.

doi:10.1038/srep31374
18. Cunning R, Silverstein RN, Barnes BB, Baker AC. Extensive coral mortality and critical habitat loss following dredging and their association with remotely-sensed sediment plumes. Mar Pollut Bull. 2019;145: 185–199. doi:10.1016/j.marpolbul.2019.05.027
19. Alvarez-Filip L, Estrada-Saldívar N, Pérez-Cervantes E, Molina-Hernández A, González-Barrios FJ. A rapid spread of the stony coral tissue loss disease outbreak in the Mexican Caribbean. PeerJ. 2019;2019. doi:10.7717/peerj.8069
20. Roth L, Kramer PR, Doyle E, O'Sullivan C. Caribbean SCTLD Dashboard. 2020 [cited 6 Apr 2020]. Available: www.agrra.org
21. Landsberg JH, Kiryu Y, Peters EC, Wilson PW, Perry N, Waters Y, et al. Stony Coral Tissue Loss Disease in Florida Is Associated With Disruption of Host–Zooxanthellae Physiology. Front Mar Sci. 2020;7: 1–24. doi:10.3389/fmars.2020.576013
22. Meyer JL, Castellanos-Gell J, Aeby GS, Häse CC, Ushijima B, Paul VJ. Microbial Community Shifts Associated With the Ongoing Stony Coral Tissue Loss Disease Outbreak on the Florida Reef Tract. Front Microbiol. 2019;10: 2244. doi:10.3389/fmicb.2019.02244
23. Rosales SM, Clark AS, Huebner LK, Ruzicka RR, Muller EM. Rhodobacterales and Rhizobiales Are Associated With Stony Coral Tissue Loss Disease and Its Suspected Sources of Transmission. Front Microbiol. 2020;11: 1–20. doi:10.3389/fmicb.2020.00681
24. Aeby GS, Ushijima B, Campbell JE, Jones S, Williams GJ, Meyer JL, et al. Pathogenesis of a Tissue Loss Disease Affecting Multiple Species of Corals Along the Florida Reef Tract. Front Mar Sci. 2019;6: 678. doi:10.3389/fmars.2019.00678
25. Neely K. Ex-Situ Disease Treatment Trials Completed in Fulfillment of PO B2B8A4 and

PO B30DB3 for. 2018.

26. Neely K, Hower E. FY 2018 In-Situ Disease Intervention. 2018. Available: https://floridadep.gov/sites/default/files/Large-Coral-Disease-Intervention-Summary-Report.pdf

27. Voss JD, Richardson LL. Nutrient enrichment enhances black band disease progression in corals. Coral Reefs. 2006;25: 569–576. doi:10.1007/s00338-006-0131-8

28. Dalton SJ, Smith SDA. Coral disease dynamics at a subtropical location, Solitary Islands Marine Park, eastern Australia. Coral Reefs. 2006;25: 37–45. doi:10.1007/s00338-005-0039-8

29. Courtney LA, Fisher WS, Raimondo S, Oliver LM, Davis WP. Estimating 3-dimensional colony surface area of field corals. J Exp Mar Bio Ecol. 2007;351: 234–242. doi:10.1016/j.jembe.2007.06.021

30. Westoby MJ, Brasington J, Glasser NF, Hambrey MJ, Reynolds JM. "Structure-from-Motion" photogrammetry: A low-cost, effective tool for geoscience applications. Geomorphology. 2012;179: 300–314. doi:10.1016/j.geomorph.2012.08.021

31. Harborne AR, Mumby PJ, Ferrari R. The effectiveness of different meso-scale rugosity metrics for predicting intra-habitat variation in coral-reef fish assemblages. Environ Biol Fishes. 2012;94: 431–442. doi:10.1007/s10641-011-9956-2

32. Burns JHR, Delparte D, Gates RD, Takabayashi M. Utilizing underwater three-dimensional modeling to enhance ecological and biological studies of coral reefs. Int Arch Photogramm Remote Sens Spat Inf Sci - ISPRS Arch. 2015;40: 61–66. doi:10.5194/isprsarchives-XL-5-W5-61-2015

33. Leon JX, Roelfsema CM, Saunders MI, Phinn SR. Measuring coral reef terrain roughness

using “Structure-from-Motion” close-range photogrammetry. Geomorphology. 2015;242: 21–28. doi:10.1016/j.geomorph.2015.01.030

34. Cocito S, Sgorbini S, Peirano A, Valle M. 3-D reconstruction of biological objects using underwater video technique and image processing. J Exp Mar Bio Ecol. 2003;297: 57–70. doi:10.1016/S0022-0981(03)00369-1

35. Gutiérrez-Heredia L, D’Helft C, Reynaud EG. Simple methods for interactive 3D modeling, measurements, and digital databases of coral skeletons. Limnol Oceanogr Methods. 2015;13: 178–193. doi:10.1002/lom3.10017

36. Ferrari R, Figueira WF, Pratchett MS, Boube T, Adam A, Kobelkowsky-Vidrio T, et al. 3D photogrammetry quantifies growth and external erosion of individual coral colonies and skeletons. Sci Rep. 2017;7: 1–9. doi:10.1038/s41598-017-16408-z

37. Lange ID, Perry CT. A quick, easy and non-invasive method to quantify coral growth rates using photogrammetry and 3D model comparisons. Methods Ecol Evol. 2020;11: 714–726. doi:10.1111/2041-210X.13388

38. Bythell J, Pan P, Lee J, J. B, P. P, J. L, et al. Three-dimensional morphometric measurements of reef corals using underwater photogrammetry techniques. Coral Reefs. 2001;20: 193–199. doi:10.1007/s003380100157

39. Lavy A, Eyal G, Neal B, Keren R, Loya Y, Ilan M. A quick, easy and non-intrusive method for underwater volume and surface area evaluation of benthic organisms by 3D computer modelling. Methods Ecol Evol. 2015;6: 521–531. doi:10.1111/2041-210X.12331

40. Young GC, Dey S, Rogers AD, Exton D. Correction: Cost and time-effective method for multi-scale measures of rugosity, fractal dimension, and vector dispersion from coral reef

3D models (PLoS ONE (2018)12: 4 (e0175341) DOI : 10.1371/journal.pone.0175341). Metze K, editor. PLoS One. 2018;13: 1–18. doi:10.1371/journal.pone.0201847

41. Walker BK. Southeast Florida reef-wide Post-Irma coral disease surveys. 2018. Available: https://floridadep.gov/sites/default/files/Southeast-Florida-Reef-Wide-Post-Irma-Coral-Disease-Surveys_0.pdf

42. R Core Team. A Language and Environment for Statistical Computing. R Foundation for Statistical Computing. Vienna, Austria; 2020. p. https://www.R-project.org. Available: http://www.r-project.org

43. Oksanen AJ, Blanchet FG, Kindt R, Legen- P, Minchin PR, Hara RBO, et al. Community Ecology Package. … Ecology Package …. 2012. p. 263. doi:10.4135/9781412971874.n145

44. Martinez Arbizu P. pairwiseAdonis: Pairwise multilevel comparison using adonis. R package version 0.4. 2020.

45. Gilliam DS, Walton CJ, Brinkhuis V, Ruzicka R, Colella M. Southeast Florida Coral Reef Evaluation and Monitoring Project 2014, Year 12. Florida DEP Report #RM085. 2015; 43. Available: https://floridadep.gov/sites/default/files/SECREMP_2014.pdf

46. Jones NP. Spatiotemporal change in the benthic community of southeast Florida. 2018; 1–60. Available: https://nsuworks.nova.edu/occ_stuetd

47. Walker BK, Brunelle A. Southeast Florida large (> 2 meter) diseased coral colony intervention summary report Florida Department of Environmental Protection Coral Reef Conservation Program Florida Fish and Wildlife Conservation Commission Southeast Florida large (> 2 meter). 2018.

48. Brown DC. Close-range camera calibration. Symp Close-Range Photogramm. 1971;37:

855–866. Available: https://www.asprs.org/wp-content/uploads/pers/1971journal/aug/1971_aug_855-866.pdf

49. Pohlert T. PMCMRplus: Calculate pairwise comparisons of mean rank sums. 2020. Available: https://cran.r-project.org/package=PMCMRplus

50. Pohlert T. The Pairwise Multiple Comparison of Mean Ranks Package (PMCMR). R package. 2014. p. 27. Available: http://cran.r-project.org/package=PMCMR

51. Walton CJ, Hayes NK, Gilliam DS. Impacts of a regional, multi-year, multi-species coral disease outbreak in Southeast Florida. Front Mar Sci. 2018;5: 323. doi:10.3389/fmars.2018.00323

52. Nathan AJ, Scobell A. How China sees America. Foreign Aff. 2012. doi:10.1017/CBO9781107415324.004

53. Selig ER, Drew Harvell C, Bruno JF, Willis BL, Page CA, Casey KS, et al. Analyzing the relationship between ocean temperature anomalies and coral disease outbreaks at broad spatial scales. 2011; 111–128. doi:10.1029/61ce07

54. Frias-Lopez J, Bonheyo GT, Jin Q, Fouke BW. Cyanobacteria associated with coral black band disease in Caribbean and Indo-Pacific reefs. Appl Environ Microbiol. 2003;69: 2409–2413. doi:10.1128/AEM.69.4.2409-2413.2003

55. Weil E, Cróquer A, Urreiztieta I. Yellow band disease compromises the reproductive output of the Caribbean reef-building coral Montastraea faveolata (Anthozoa, Scleractinia). Dis Aquat Organ. 2009;87: 45–55. doi:10.3354/dao02103

56. Beal J, Voss J, Edge S, Cohen L. Assessment of coral stressors on St. Lucie Reef: Florida’s northernmost coral reef. Final Rep State Wildl Grant USFWS T-91-1. 2012. Available:

http://fau.digital.flvc.org/islandora/object/fau%3A2737/datastream/OBJ/view/Assessment_of_coral_stressors_on_St__Lucie_reef__Florida___s_northernmost_coral_reef.pdf

57. Ben-Haim Y, Zicherman-Keren M, Rosenberg E. Temperature-regulated bleaching and lysis of the coral Pocillopora damicomis by the novel pathogen Vibrio coralliilyticus. Appl Environ Microbiol. 2003;69: 4236–4242. doi:10.1128/AEM.69.7.4236-4242.2003

58. Bruno JF, Selig ER, Casey KS, Page CA, Willis BL, Harvell CD, et al. Thermal stress and coral cover as drivers of coral disease outbreaks. Roberts C, editor. PLoS Biol. 2007;5: 1220–1227. doi:10.1371/journal.pbio.0050124

59. Voss JD, Richardson LL. Coral diseases near Lee Stocking Island, Bahamas: Patterns and potential drivers. Dis Aquat Organ. 2006;69: 33–40. doi:10.3354/dao069033

60. Zvuloni A, Artzy-Randrup Y, Katriel G, Loya Y, Stone L. Modeling the Impact of White-Plague Coral Disease in Climate Change Scenarios. PLoS Comput Biol. 2015;11: 1–22. doi:10.1371/journal.pcbi.1004151

61. Harvell DC, Jordán-Dahlgren E, Merkel S, Rosenberg E, Raymundo LJ, Smith GW, et al. Coral disease, environmental drivers, and the balance between coral and microbial associates. Oceanography. 2007;20: 172–195. doi:10.5670/oceanog.2007.91

62. Roberts KE, Hadfield JD, Sharma MD, Longdon B. Changes in temperature alter the potential outcomes of virus host shifts. PLoS Pathog. 2018;14: 1–16. doi:10.1371/journal.ppat.1007185

63. Meiling S, Muller EM, Smith TB, Brandt ME. 3D Photogrammetry Reveals Dynamics of Stony Coral Tissue Loss Disease (SCTLD) Lesion Progression Across a Thermal Stress Event. Front Mar Sci. 2020;7. doi:10.3389/fmars.2020.597643

64. Neely K. Florida Keys Coral Disease STrike Team: FY 2019/2020 Final Report. Miami,

FL; 2020.

65. Figueira W, Ferrari R, Weatherby E, Porter A, Hawes S, Byrne M. Accuracy and precision of habitat structural complexity metrics derived from underwater photogrammetry. Remote Sens. 2015;7: 16883–16900. doi:10.3390/rs71215859

66. Brandt ME, Smith TB, Correa AMS, Vega-Thurber R. Disturbance Driven Colony Fragmentation as a Driver of a Coral Disease Outbreak. Harder T, editor. PLoS One. 2013;8: 1–10. doi:10.1371/journal.pone.0057164

Supporting i~~I~~nformation

~~**S1 Table. Statistical outputs for single-factor Kruskal-Wallis and pairwise Dunn's tests for all three sites: BC1, T328, and FTL4. Non-significant *p* values listed as "ns".**~~

S1~~2~~ Table. Kruskal-Wallis test comparing rate of tissue loss and site. Non-significant *p* values are listed as "ns".

~~**S3 Table. Spearman's rank correlation comparing rates of tissue loss with total colony size. Non-significant *p* values area listed as "ns".**~~
~~**S4 Table. Statistical outputs for single-factor Kruskal-Wallis and pairwise Dunn's tests for each prism shape (square, rectangle, circle) conducted for surface area and error datasets. Non-significant *p* values represented as ns.**~~

~~**S5 Table. Statistical outputs for multivariate main and pairwise PERMANOVAs across prism shapes (square, rectangle, circle) conducted for surface area and error datasets. Non-significant *p* values represented as ns, asterisks denote significant pairwise comparisons where main model terms were not significant, and NAs indicate where no pairwise comparisons were conducted.**~~

S1 Fig. Panel of prism template and deployment for underwater 3D photogrammetry. A) Template used for five sides of prism, scaled to produce replicates of three standardized shapes: square (40.32 cm^2), rectangle (12.90 cm^2), and circle (45.60 cm^2) simulating surface area measurements on a coral colony. B) Deployment of prism and scaling frames with diver recording continuous video in a lawnmower-pattern at an approximate ~~altitude~~ distance of 1 m. C) Overhead view of prism and scaling frames, with 10 cm banded tape for scaling. D) Deployment of prism and scaling frames on a reef environment at Lauderdale-by-the-Sea, FL.

~~**S2 Fig. Plot of mean change in tissue area on fate-tracked *M. cavernosa* colonies from pairwise time comparisons (T_1–T_2, T_2–T_3, T_3–T_4).**~~

~~**S3 Fig. Spearman's rank correlations between (a) disease lesion area and total colony area, (b) number of disease lesions and total colony area, and (c) number of disease lesions and disease lesion area. Significant correlation shown as trendline with 95% CI for (c).**~~

S2~~4~~ Fig. Panel of ~~surface area and~~ error measurements for each of the three prism shapes (square, rectangle, circle~~; columns~~), ~~. For shape surface areas (top row), the black lines indicate the template shape areas. Shape errors (middle row) were~~ calculated as the absolute difference between measured shape surface areas and template areas. ~~Overall *p* values represent results of single-factor Kruskal-Wallis tests comparing shape measurements across sites, and different letters denote significant pairwise comparisons between sites as generated by Dunn's tests. Shape errors were also correlated to overall model area (bottom row) using correlation analyses, with Spearman's rho (r_s) and *p* values given for each site.~~

S1 Dataset. Dataset containing disease prevalence data from roving diver disease surveys.

S2 Dataset. Dataset containing tissue area measurements from rendered 3D models of fate-tracked *Montastraea cavernosa* colonies.

S3 Dataset. Dataset containing ~~model area,~~ shape surface area and~~, and shape~~ error measurements from prism deployment across three sites (pool, Lauderdale-by-the-Sea, St. Lucie Reef). Error was calculated as the absolute difference between measured surface areas and actual area of the template shapes, then standardized as a percentage relative to template area.

Editor:

We note that Figure 2 in your submission contain map images which may be copyrighted. All PLOS content is published under the Creative Commons Attribution License (CC BY 4.0), which means that the manuscript, images, and Supporting Information files will be freely available online, and any third party is permitted to access, download, copy, distribute, and use these materials in any way, even commercially, with proper attribution. For these reasons, we cannot publish previously copyrighted maps or satellite images created using proprietary data, such as Google software (Google Maps, Street View, and Earth). For more information, see our copyright guidelines: http://journals.plos.org/plosone/s/licenses-and-copyright.

-Figure 2 in this manuscript is original; it was made using ArcGIS and open source shapefiles referenced in the figure caption. The figure caption has been updated to reflect the changes suggested by the PLOS ONE editorial board. (Florida boundary and benthic hard bottom shapefile source: Florida Fish and Wildlife Conservation Commission-Fish and Wildlife Research Institute). The email suggesting these changes is pasted below:

PONE-D-20-25422R1

Quantifying impacts of stony coral tissue loss disease on corals in Southeast Florida through surveys and 3D photogrammetry

PLOS ONE

Dear Mr. Combs,

Thank you for enquiring further regarding the copyright request. Based on the information you provided, the shapefiles/map data appear to be in the Public Domain and therefore may be included in your submission figures.

While you are not required to provide attribution for data in the Public Domain, the dataset Metadata (https://www.arcgis.com/sharing/rest/content/items/baeae4a8572b411c825101d099d648cc/info/metadata/metadata.xml?format=default&output=html) do does state: "Acknowledgement of the FWC-FWRI (Florida Fish and Wildlife Conservation Commission-Fish and Wildlife Research Institute) as the data source would be appreciated in any products developed from these data, and such acknowledgment as is standard for citation and legal practices for data source is expected by users of this data. Please cite the original metadata when using portions of the

record to create a similar record of slightly altered data, such as reprojection. If any data are modified or adjusted, please share the edited information with FWC."

Therefore, we ask that you do reference the two datasets in the caption for Fig 2 in your manuscript, and upload the revised manuscript to the Editorial Manager submission system. If any further questions about this arise, please don't hesitate to reach out.

Regards,

Lilla Petho

PLOS ONE

Reviewer 1 in particular notes that there is a significant literature on photogrammetry techniques on coral reefs, and further attention to this would improve both the introduction and discussion, rather than relying on citations to other taxa. They also require increased clarity in the methods regarding validation of the accuracy of the model, including what site-specific community parameters would impact the 3D model generation. Reviewer 2 also brings up a salient point here, which is that there was no direct comparison to 2D methods, so claims of increased accuracy are not demonstrated. They do recommend that a relatively rapid post-hoc statistical comparison with 2D screen grabs could be considered, or more cautious language. I would suggest the former to increase the rigor of your study, but either would be sufficient. Reviewer 1 also questions the clarity of statistical analysis, and believes that further investigation into why shape area and error varies with location is either required, or explained in the text.

-We took only non-scaled reference photographs and thus cannot conduct a 2D comparison with our 3D data, as such we chose to use more cautious language throughout the manuscript.

Both reviewer 1 and I are curious as to why the grouping of disease prevalence is at the county level rather than the location/site scale, particularly since you do not discuss different management, water quality or land use differences between counties (See Fig 3). By working at the location scale, this would allow comparison of more similar replicates, and would also be more accessible to a person not familiar with the geography of Florida. If you feel that county is an important part of the puzzle that makes it a more appropriate unit of replicate than location (more than just indicating a north - south gradient for example), it would be useful to explain this decision.

-We agree with the editor and reviewers comments and reanalyzed the survey data to group at the Location level.

Both reviewers identify the recommendation of 'culling' as unjustified, or presented with too high a level of certainty, particularly since the effort, efficacy and description of what other interventions are available are not given (if there is only one colony in an area infected, but it is large, why not remove that if it leads to a better outcome than other interventions?). Without evidence presented here, or citations to small colonies dying more quickly or being disease vectors, this management recommendation goes far beyond the work. Indeed, in Fig S2 It could be argued that there is some evidence that some larger colonies might lose tissue at a faster rate than some small colonies. In the discussion you also state that "disease lesion area did not correlate with total colony size suggesting that larger colonies do not exhibit a higher proportion of diseased tissue" - this could imply they show the same proportional loss or lower. I strongly suggest you present more data on proportional loss of colony tissue (not just absolute loss) if this is an argument you wish to make. Even so, suggesting culling small colonies as a management measure would still be extreme and would require a stronger argument with references to small colonies as vectors or disease reservoirs. The argument also begs the question - what is a small colony? Nor are what interventions that might be possible shy of culling the entire colony described.

-We removed the suggestion of culling from the manuscript.

Finally, there is a significant amount of relevant context and data in the SI. It would be good to see some of the tables and figures into the main text, particularly concerning the disease incidence, colony size and disease spread data.

-We revised our results section to remove unnecessary metrics and focused on the more compelling outcomes from this study. We also included additional figures focusing on correlation between colony size and disease area as well as rate of tissue loss.

Reviewer 1:

Reviewer #1: Reviewer comments:

1) from L88: I think applications on cetacean and elasmobranch research are way out of the scope of this work. Better concentrate on referencing all the exciting research on coral reefs.

- We have removed the unnecessary references to cetacean and elasmobranch research and greatly increased our literature review concerning photogrammetry on corals and coral reefs. We appreciate the suggested journal articles.

2) Also, in this section, I don’t think you need to cite aerial and scanner methods to prove your

point, and the two studies you cite for coral growth did not use photogrammetry at all. Use Ferreira et al. 2017 and Lange & Perry 2020 if you want to keep this sentence. Alternatively you could explain photogrammetry and SfM first and then give examples of studies on reefs. Also see annotations in the PDF.

- Reference to aerial and scanner methods have been excluded and the focus was shifted to in-water photogrammetry methods, we also corrected the two citations for Ferreira et al. 2017 and Lange & Perry 2020.

3) L. 104: optimized from what basis? You have not developed the photogrammetry technique, but a new application for it. Maybe say: "In this study we used photogrammetry techniques as described in [Young et al.] in order to develop a new application, i.e to track SCTLD disease progression in M. cavernosa colonies." or something similar?

-We revised the wording of this to better represent our work (L109).

Methods:

1) see annotations in PDF to improve clarity of the text.

-The PDF annotations were applied, they were very helpful and the authors thank the reviewer for their efforts to improve the clarity and writing throughout this manuscript.

2) L181: state version of Agisoft Metashape that you used as some versions seem to have issues and it will help for repeatability.

-This has been included (L181).

3) L. 215: I do not understand why site-specific "community parameters" (what is this anyways?) or even water quality should affect 3D model generation. This would mean that the method is not accurate and should not be used.

- Our intention was to compare surface area error from models constructed in varying environments (pristine pool versus nearshore coral reef with low visibility and high wave action). To make the model accuracy validation section more clear and applicable to the fate-tracking results, we have focused on comparisons of shape error between the pool models and models made at the fate-tracking sites in Lauderdale-by-the-Sea. We have rephrased this section to be more explicit starting at L214.

Related to this it does not make sense to compare the area of shapes among locations to evaluate the 3D models. So I am wondering why you did not just photograph the mock colony at the sites where you did the fate-tracking in order to calculate the accuracy of your methodology.

- Models with the mock coral colony were constructed at the same fate-tracking sites in the original version of the manuscript, but were not represented as separate sites in the original analysis. To improve the comparability of the model accuracy analyses to the fate-tracking portion, we have focused on the fate-tracking sites in Lauderdale-by-the-Sea only. Likewise, we have removed the analyses of overall shape areas and focused on the shape error associated with each model.

Concerning this there is two parameters you want to check:

A) accuracy, meaning how close to reality is your 3D model. Test this by comparing measured shape areas on your mock corals to known areas and calculate the mean error. (This is potentially how you got your 2.17 cm2?) If you notice that the error is bigger at a more turbid reef site, then you could conclude that the visibility affects the accuracy of model building/measurement. But direct comparison of shape areas among sites does not make sense.

B) precision, meaning how good is the reproducibility of your measurements. For this you should measure the surface of the same shape, or better the same coral colony, several times, including all the hand tracing around colonies etc. Then calculate the error, which will show you if you introduce considerable variability using your workflow.

You should calculate the coefficient of variation (error/average) in order to compare your errors to other studies doing surface area measurements. You cannot compare the error from small shapes (2 cm2) directly to the surface area of the colony.

- We have made substantial efforts in the data analysis and writing to make this section of the manuscript clearer and more applicable to the coral fate-tracking portion. First, we focused on comparison of model error across the pool and the three coral fate-tracking sites in Lauderdale-by-the-Sea. The statistical analyses were revised to a comparison of shape error (i.e. the difference between 3D modeled shape areas and the template) among sites (L214-222, L225-230, L308-3111). Replicate measurements of all the prism shapes were made within the original analyses, but this was not clear in the original version. The prism had five faces with the template shapes (top and four sides), each of which had multiple shapes (four squares and rectangles, one circle per face) (L222-225). Shape error is also represented in two ways, as 1) the absolute difference between measured shape areas and template area, and 2) percent error relative to template area (similar to previous studies, including those you suggested in the original review). The former is used for the statistical tests and for visual assessment of shape error in S4 Fig, and the latter is reported in the text (L311-314). These calculations result in mean errors of 1.70 sq. cm and 6.13%, respectively. The coefficient of variation was not calculated for these analyses, as this is typically used for comparisons of replicate 3D models or measurements. Each of the measurements in our prism dataset are statistically independent, and we feel provide for a robust assessment of model accuracy.

L 240: I do not understand why these correlations were done and what they would tell us about the accuracy of the method.

- This analysis has been removed, and the section has been rewritten according to the revised analysis of accuracy across sites.

Results:

1) I am wondering why did you choose to compare counties instead of locations as shown on the map? The latter would have the advantage that replications are more similar.

-The disease prevalence survey analyses were shifted from county level to location level as shown on the map.

2) L 255-260: I don't think the figure supports these statements. I would say something along the line of “in Broward, disease prevalence stayed quite constant at around 10%, while in Palm Beach prevalence was usually very low, but peaked in March 2019 when ... of colonies were affected. Disease prevalence at Martin was very variable due to low numbers of live coral colonies...”

-This section has been edited to improve clarity and better reflect our results and figures (L238-255).

3) L 273: Has it been taken into account that colonies were measured repeatedly? Considering that the areas of your colonies are very different you might either have do more fancy statistics using repeated measures GLM/GAM (maybe using colony ID as fixed factor and site and time as explaining variables? sorry, not an expert myself) or you might have to calculate “loss of area” or “loss % of area” in order to make meaningful comparisons over time.

I know you calculated rate of change in healthy and diseased tissue, which I think might make more sense than comparing actual area. In general it is getting a bit confusing looking at so many parameters. I would suggest to think carefully which parameters are most useful in showing what you are interested in and rather use fewer but explain better what they mean.

E.g., I think the most interesting results and the best order in the section L277-309 would be (add numbers and statistics): “The rate of tissue loss did not differ among sites but was variable over time, with highest loss in the first observation interval. The diseased area however did stay constant over time, indicating that the lesion moves with the infected tissue.” Then add the correlations you think are useful. Not all are I think.

-The Friedman’s test and subsequent post hoc analysis, the Nemenyi test, are appropriate since our data is not normally distributed; it is a non-parametric equivalent to a repeated measures analysis of variance. The section has been reduced in the number of metrics and tests run to increase overall clarity and focus on important, compelling results. (L289-297). Proportion of loss data was added, and the only correlation that was retained, as the authors thought this was the most compelling, was the correlation between disease lesion area and total colony size.

4) L287 and 288: S2 Fig should be S3 Fig?

- These were removed.

5) L 312-L334: This whole section does not make sense to me. I do not understand why the areas of shapes should be different depending on location. This definitely does not increase trust in the method! It might in part be a relic of your different sample sizes and tests you do. Why do you run ANOVA then Kruskal Wallis and then 3-way PERMANOVA? I fail to understand what any of these significances mean and it is not explained in the results or discussion sections.

- In the revised version of the manuscript, we have streamlined our assessments of model accuracy according to previous suggestions. We hope that the new sections present a better description of the analyses that were conducted. Also, following careful review of the statistical tests and 3D models, we identified a few outliers in the prism dataset that were the result of holes in the mesh leading to inaccurate surface area measurements, which appeared to be driving the significant differences presented in the original manuscript.

I would suggest to think carefully about what parameters actually tell you something about the accuracy/precision of the analysis (see comments above) and rewrite this section completely after the improved analysis.

- Thank you for your constructive comments, we hope that the revised sections address your concerns and suggestions.

Discussion:

This section could be improved by being very clear how the results compare to other studies and what can be said about the implications. See annotations in the PDF

1) L353: It is not clear what you are trying to say here. Also please make clear what you mean with L356-357. Next section can be shortened as suggested in PDF.

- This section, along with the section below, were combined and rewritten to improve clarity (L324-350).

2) L363: This paragraph seems to be repeating discussions from the previous paragraph. Maybe you could combine those better without repeating yourself?

I think the order of these two paragraphs could be improved. E.g. “In the FRT, disease prevalence is typically higher (...%) than elsewhere in the TWA (~1-3%) [57]. Sites in Palm Beach in the present study showed relatively low background disease (6%), similar to prevalence across the NFRT after Hurricane Irma in Sept 2017 (6%???) (Walker 2018, 53). Highest values

observed in this study were 20-45% at sites in Martin, but did not reach levels of up to 60% as reported in [17], likely due to low abundance of susceptible species after ongoing SCTLD impacts [53,58]"

-The following section was changed using your suggestions to increase clarity (L332-338).

3) L384: I thought Fig S2 showed that it does NOT correlate? Otherwise your next sentence does not make sense

- This line was edited to correct the typo, the sentence should have read "rates of tissue loss did not correlate" as stated in the reviewer comment.

4) I think the suggestion to cull small colonies is a bit drastic and not supported by your study. If there is other research supporting this approach please cite here.

- The suggestion of culling has been removed from the manuscript

5) L384-393 could be condensed down to 1-2 sentences saying management should target larger colonies.

-This section was revised to omit culling and express the need to target larger colonies.

6) From L 403: This whole sections would have to be revised after analysis of accuracy/precision.

- Following the revised analysis, we have rewritten this section to emphasize the error rate in the prism measurements (L379-382), the potential causes (flexible shape templates and water movement/poor visibility; L382), and suggestions for improving model construction and underwater filming/photography (L384-391).

7) L406: It is not possible to compare the 2.17 cm2 shape error directly to the much larger colonies. Actually, this error seems very high considering that the shape on your mock coral is not very big?! Does that relate to about 10%? Calculate CV and compare to other studies. Also, if you have an error of about 10% you might want to check if the difference of total area/healthy area between time points would still be significant.

- Based on previous studies, we have opted to calculate error rate (%) for shape measurements relative to the corresponding template area. As a result, the global error rate is ~6%.

8) L409: you did not test the effect of colony size on model accuracy. Also, it does not make sense that water quality (do you mean turbidity/visibility?) affects the size of colonies. You are probably trying to say that lower visibility could result in lower model quality affecting measurements of surface area? Be precise in your wording.

- We have revised this section to be more specific regarding surface area measurements and potential effects of low visibility on model construction and error (L389).

Conclusions and general:

1) Different kinds of interventions should be mentioned in the introduction or discussion if it is the main discussion point in the conclusion.

-There has been a wide variety of intervention methods proposed, and refinement and evaluation is still ongoing, so we did not go into detail on the specific methods, but did include them in the introduction (L77-82).

2) L435: Why would interventions be unsuccessful in small colonies? They should work the same, just preserve less total area, right? So I understand prioritizing big ones, but in sites were prevalence is low why not treat small ones too.

-The reference to interventions being unsuccessful was removed and clarification was added to imply that larger colonies should be prioritized if present, but smaller colonies should not be ignored all together.

3) I think the discussion and conclusion sections will benefit from a second round of review after the improved model evaluation. Try to be very clear what the novelty of this work is (new application of photogrammetry method to accurately quantify tissue loss/disease progression over time) and what can be concluded from it (improve survey protocols, evaluate success of intervention methods... I don't think culling of half the colonies is a good outcome here).

-The discussion and conclusions have been revised to focus more on the novelty of this work, comparable photogrammetry and SCTLD studies, and improving the management and intervention suggestions throughout.

4) If you want people to take up this method, make the workflow easier to access. I know it is all in the GitHub repository, which is awesome, but I did not easily find the step-by-step guide and what you actually did to measure the areas. Maybe you could prepare a one-pager which is easy to print, stating the steps of image acquisition, model building and analysis to go into Suppl Methods. Or put a link in the methods which leads straight to the guide instead of the whole repository.

- A reference to the protocol within our GitHub repository was added to the methods section. (L178)

5) Also, it would be great if you could make the models (as .obj?) available in the repository.

-Unfortunately, the models are too large to be housed on the GitHub repository, they will be available upon request.

Reviewer 2:

General comments
- One of the major findings of this study is that the 3D model method presented is "more accurate data than previously established methods such as two-dimensional surface area estimation (ln 398-399)." I thought the assessment of the accuracy of the 3D method presented here was excellent and rigorous, but there was not a estimate of the disease lesions or the calibration templates/mock coral made from the same two-dimensional photographs to make a robust and unbiased statistical comparison of the two techniques. While I agree that this 3D model pipeline is achievable, it does still require ~40 min per colony of rendering, plus fairly expensive software and hardware for modelling and storage of large video files compared with the much simpler 2D method, so a quantitative comparison of the two methods would strengthen the argument and add justification for using the 3D model method presented here.

-We took 2D reference photographs in order to double check possible abnormalities in the model (eg: if that is a Christmas tree worm or a small disease lesion) they are not scaled and therefore cannot be analyzed and used in a comparison of the two techniques. However, we have incorporated references to previous studies that have compared 3D photogrammetry to 2D and physical methods (tin foil method) that have shown 3D photogrammetry to be a more robust method.

- The inclusion of more data from the disease surveys in the main text could be useful, and would balance the disease ecology with the methodological aspects of the study a bit better. Including which species were (and were not) affected at each site, and how disease prevalence changed over time by taxa within each site would be a nice addition to the main text. Line 365-7 mentions the "low abundance and species richness in Marin County sites compared with PB and Broward Counties", but what is the density and species richness within each site?

-We feel that adding broader ecological surveys of these reefs outside of SCTLD prevalence is outside the scope of this study. We included more species abundance data, mainly to highlight the lack of 'highly susceptible' species within our surveys is possibly due to SCTLD already and how different the coral communities are at SLR relative to the rest of our sites.

- The manuscript is relatively sparse wrt figures and tables, with the vast majority of the information presented as supplementary. I would suggest including a few of these in the main text, particularly Fig S2 and possibly S4, again to balance out the ecology/methodology aspects of the manuscript.

We included additional figures focusing on correlation between colony size and disease area as well as rate of tissue loss.

Several Specific comments
- Line 193 – it is quite tricky to determine whether the stark white area of the lesion is still alive or newly dead, especially in an image/video. Would it be more appropriate to define the lesion area as the 'stark white coral tissue or very newly dead white skeletal area'? If not, how was this distinction made? It is very difficult to tell in Figure 1, but the white lesion areas look like there is no live tissue to me.

- We revised this to improve clarity (L193)

- Line 282 – consider rounding the estimates of # of lesions per colony to the nearst whole number as that makes the most conceptual sense (or at least to the 10th which is probably a more appropriate significant digit)

-These were removed from the analysis .

- Line 360 – consider stating what the most susceptible species are.

-This has been included (L340)

- Line 384 – was the correlation between rates of tissue loss and total colony size positive or negative? Please specify.

-This was a typo, it should have read "did not" correlate, it has been adjusted (L361)

- Line 388 – typo – suggest 'greater preservation'

- This section has been rewritten

- Line 389 – is there justification for the statement that smaller colonies are more likely to succumb to SCTLD? It may take less time to succumb due to the rate of tissue loss, but is the likelihood of dying from the disease actually higher? Has much recovery been observed? I would suggest editing to "smaller infected colonies may succumb more quickly to SCTLD"

-This section was heavily revised as suggested and the statement regarding small colonies has been omitted.

- Line 406 – perhaps you can expand on what you mean by 'sufficient replication'? Does this mean replicate videos of each colony to render multiple models per colony?

-This section has been heavily revised and this line has been removed.

- Line 413 – suggest ‘most commonly’

-This section has been heavily revised and this line has been removed.

- Line 434 – suggest ‘regarding’ rather than ‘relative to’

-This has been changed as suggested (L406)

- I wonder if it is worth commenting on how this model approach may work for other coral growth morphologies. Mcavernosa is a fairly simple mounding shape, but encrusting, foliose or branching corals may be much more difficult to accurately model?

- I have strong reservations about the mitigation technique of culling small colonies that is suggested in line 438 (and 389, see comment above). I understand that they may die more quickly than larger colonies and could act as sources of the disease. But the data presented here do not indicate that they are more susceptible (are there size-frequency distributions of healthy vs/ diseased corals in a population?), and the small colonies are often offspring of the few remaining colonies that have survived multiple selection events already and may be the best hope of the recovery of resilient communities, especially if there is any evidence that colonies can recover from this disease. Thus I much prefer the second option of focusing intervention resources on larger colonies and would urge caution when considering culling of smaller colonies.

-The suggestion of culling has been removed throughout the manuscript.

- Fig 1. Typo in fig legend – add ‘a’ before ‘colony’

-This has been changed as suggested.
