# Supplementary material for: Quantifying impacts of stony coral tissue loss disease on corals in Southeast Florida through surveys and 3D photogrammetry
Source: PLoS One. 2021 Jun 25;16(6):e0252593. doi: 10.1371/journal.pone.0252593 (PMC8232449; doi:10.1371/journal.pone.0252593)
Supplement: Attachment — Submitted filename: responseToReviewersV2.docx [file pone.0252593.s010.docx]

Response to reviewers:

Reviewer #1: Reviewer 1 Round 2

The authors did a good job addressing my questions and suggestions in their revised version of the manuscript and improved the photogrammetry method section and discussion section significantly. Well done!

Please find below a couple of very minor suggestions I noted down while reading over the manuscript, otherwise I am happy to recommend publication as is.

- **Thank you very much for taking the time to review this manuscript again, and for providing additional edits to improve readability. We have addressed your comments in the line-by-line responses below.**

Check Figures 5 and 6 and the captions and references in the text. Figure 5 includes the disease lesion area plot (but note different font size) but this is not represented in the figure caption and text references and Figure 6 depicts the same plot.

- **Thank you for catching this, this was the result of a small error in the R code used to generate figures. The correct figures have now been included.**

L173: hovered approximately 1 m above

- **Changed as suggested**

L176: overlap of filmed surface area

- **Changed as suggested**

L183: (link does not work)

- **Link has been fixed**

L249: site SLR North in April 2019

- **Changed as suggested**

L253: What do you mean with species abundance? Overall colony abundance? or number of species? Also, was this parameter higher or lower at SLR (says varying in L254)

- **Reworded to signify as colony abundance and lower abundance at SLR relative to all other sites.**

L289: tissue area (get rid of s)

- **Changed as suggested**

L313: This first sentence seems out of place. Maybe better integrates in method section as the reason to use PERMANOVA. Then start with “Absolute and relative shape error did not vary across the pool and ...”

- **Changed as suggested**

L316: Absolute shape error

- **Changed as suggested**

L318: corresponded to a relative shape error of

- **Changed as suggested**

L322: on measured colony surface area

- **Changed as suggested**

L332-334: consider to move this sentence behind L337 starting with “Other ... observed over similar spatial scales” (as you note similar distance to Belize/Martinique)

- **Changed as suggested**

L342: no site (get rid of s)

- **Changed as suggested**

L343: near Miami in ...(year)

- **Changed as suggested**

L346: comparatively sparse (or absent)

- **Changed as suggested**

L360: on a coral colony level over just ... over the course of this study

- **Changed as suggested**

L361: why “in contrast”?

- **This has been removed to avoid confusion.**

L374: sufficient area (get rid of s)

- **Changed as suggested**

L379: progression on a colony-level

- **Changed as suggested**

381: I would not consider this a complex morphology. Would rephrase to “Healthy and diseased surface area of 24 coral colonies were quantified and compared over time”

- **Changed as suggested**

385: did not vary across different depth and turbidity conditions (S2 Fig.)

- **Changed as suggested**

L386: Average absolute shape error

- **Changed as suggested**

L387: to a relative error of ...

- **Changed as suggested**

L389: rather than representing solid surfaces.

- **Changed as suggested**

L404: may be able to determine

- **Changed as suggested**

L406: the progression of disease

- **Changed as suggested**

L411: what does non-discriminant mean? Do you mean “Compared to other coral diseases, impacts of SCTLD are not yet well described concerning host-specificity and spatio-temporal distribution”? Is that true? Lots of studies out on SCTLD

- **This statement has been rewritten to emphasize that SCTLD has broad taxonomic and temporal impacts relative to other coral diseases.**

L412-416: would suggest to shorten and rephrase, e.g.: “Based on the results from this study, larger colonies should be prioritized for SCTLD mitigation measures.”

- **Changed as suggested**

L417: remove alternative

- **Changed as suggested**

L419-420:” ... longer timescales and multiple species are desirable to confirm observed patterns ... “ (3D mentioned in next sentence)

- **Changed as suggested**

L422: not sure what redundancies and precautions you refer to. I suggest “... are a valuable approach for colony fate-tracking if high resolution imagery can be obtained.“

- **Changed as suggested**

L425: more accurate than what?

- **Added language to clarify**

Reviewer #2: The manuscript by Combs et al. reports the results of spatial and temporal field surveys of SCTLD in Florida and, using the survey results to inform site selection, undertook fate-tracking of individual Montastrea cavernosa colonies at the most impacted sites. The fate-tracking applied a structure-from-motion 3D photogrammetry technique to quantify rates of tissue loss and changes to lesion area and lesion number through time. The authors then used those data to improve our understanding of disease dynamics and make management recommendations.

The revised manuscript has addressed all the major concerns I raised in my previous review, including removing mention of culling, removing statements of direct comparisons with 2D methods that were not justified by the data, reanalysing the data at the location level, and including some of the supplementary data in the main text. Overall, I think the manuscript has been clarified and streamlined will be a useful addition to the literature on this important topic. I only have a few outstanding comments that warrant a minor review, most notably about the intervention recommendation, and have added some edits and comments in an annotated PDF, which I hope the authors will find useful.

- **Thank you very much for taking the time to review this manuscript again, and for providing additional edits to improve readability. We have addressed your comments in the line-by-line responses below.**

• References: Some key references on disease ecology and SCTLD are missing, including Muller et al. 2020 Spatial Epidemiology of the Stony-Coral-Tissue-Loss Disease in Florida. Front Mar Sci 7:163; Meiling et al. (2020) and stony coral tissue loss disease (SCTLD) lesion progression slows in association with thermal stress. Frontiers in Marine Science, 7, 1128. More references are added in the annotated PDF file, and I urge the authors to review some of the key disease literature for the Caribbean in the discussion of ecological drivers including temperature, local pressures, etc.

- **Thank you for providing additional references to strengthen our conclusions. The Meiling et al. (2020) study was referenced in the first revision, but a glitch with our reference manager software caused a replacement of the Muller et al. (2020) study. We have added it back in, and have included your additional suggestions.**

• Intervention recommendation: Line 36, Line 374, Line 415: I think it is worth adding a caveat/qualifier here about what the end goal of the intervention is. If the goal is to minimise immediate loss of coral cover, then yes, perhaps it is best to prioritise the largest colonies. But, that doesn’t take into account other on-going processes. Firstly, one could argue that the smaller colonies represent younger and more locally adapted/stress tolerant genotypes, and applying intervention efforts across all colonies in a small high-value (heat tolerant)/well connected (larval source)/high-flow (i.e. pathogen source) area might be better than selecting only larger individuals in a broader area using the same amount of time/resources. Secondly, the disease appears to follow contagious transmission dynamics (Muller et al 2020), so if infected small colonies are left untreated in an area around larger colonies that are treated, it may remain a pathogen source to re-infect the larger colonies; I understand this argument led to your original suggestion of culling but that also doesn’t factor in other processes. I would urge you to add a qualifier as such and soften the language because the statement “should be prioritised” is quite strong when these other factors aren’t considered.

- **We have modified these statements to modify the language and to include caveats where relevant. Interventions have been proposed as a way to slow down immediate loss of coral cover by aiding in colony survival, but the groups leading these efforts recognize that intervention is not a sustainable approach over the long-term response to the disease outbreak. We have indicated this in the introduction and discussion at L81 and L375, respectively. Additionally, we have added a caveat in the conclusions at L413 stating that intervention efforts on larger colonies should be prioritized when resources are limited.**

• Methods: Lines 142-147: The sentences “Statistical tests were run in the R statistical environment” and “non-parametric tests were implemented for all analyses unless otherwise noted”… seemed out of place and premature, because it isn’t clear what tests were run and why? I suggest moving line 146 up to start the section with “to assess variation in disease prevalence among sites and survey times”, and then you can explain the methodological details.

- **Changed as suggested**

• Figures: It appears that Figures 5a and 6 are the same? Also, Figure 5b could be faceted by site to support the argument that tissue loss didn’t differ among sites.

- **Thank you for catching this, this was the result of a small error in the R code used to generate figures. The correct figures have now been included, and Figure 5b has been faceted by site.**
